# Integration of Hybridization Strategies in Pyridine–Urea Scaffolds for Novel Anticancer Agents: Design, Synthesis, and Mechanistic Insights

**DOI:** 10.3390/molecules28134952

**Published:** 2023-06-23

**Authors:** Sreenivasulu Godesi, Hossam Nada, Joohan Lee, Joon-Hee Kang, Soo-Youl Kim, Yongseok Choi, Kyeong Lee

**Affiliations:** 1 BK21 FOUR Team and Integrated Research Institute for Drug Development, College of Pharmacy, Dongguk University-Seoul, Goyang 10326, Republic of Korea; sreenu.godesi@dongguk.edu (S.G.); hossam_hammouda@dgu.ac.kr (H.N.); ljh6948@dgu.ac.kr (J.L.); 2 Pharmaceutical Chemistry Department, Faculty of Pharmacy, Badr University in Cairo, Cairo 11829, Egypt; 3 Graduate School of Cancer Science and Policy, National Cancer Center, Goyang 10408, Republic of Korea; wnsl2820@gmail.com; 4 Division of Cancer Biology, Research Institute, National Cancer Center, Goyang 10408, Republic of Korea; kimsooyoul@gmail.com; 5 College of Biosciences and Biotechnology, Korea University, Seoul 02841, Republic of Korea; ychoi@korea.ac.kr

**Keywords:** hybridization strategy, pyridine–urea, anticancer agents, molecular docking, molecular dynamics, MM–GBSA

## Abstract

Annually, millions of new cancer cases are reported, leading to millions of deaths worldwide. Among the newly reported cases, breast and colon cancers prevail as the most frequently detected variations. To effectively counteract this rapid increase, the development of innovative therapies is crucial. Small molecules possessing pyridine and urea moieties have been reported in many of the currently available anticancer agents, especially VEGFR2 inhibitors. With this in mind, a rational design approach was employed to create hybrid small molecules combining urea and pyridine. These synthesized compounds underwent in vitro testing against breast and colon cancer cell lines, revealing potent submicromolar anticancer activity. Compound **8a**, specifically, exhibited an impressive GI_50_ value of 0.06 μM against the MCF7 cancer cell line, while compound **8h** displayed the highest cytotoxic activity against the HCT116 cell line, with a GI_50_ of 0.33 ± 0.042 μM. Notably, compounds **8a**, **8h**, and **8i** demonstrated excellent safety profiles when tested on normal cells. Molecular docking, dynamic studies, and free energy calculations were employed to validate the affinity of these compounds as VEGFR2 inhibitors.

## 1. Introduction

Cancer is a serious public health concern around the world, and it is the second leading cause of mortality after cardiovascular diseases [1]. Cancer patients suffer from high mortality rates due to late diagnosis and delayed medical treatment [2]. According to recent estimates, there were 10.9 million newly reported cancer diagnoses, resulting in 6.7 million deaths, and 24.6 million people were still living with cancer 3 years after their diagnosis. Breast cancer and colorectal cancer were two of the most commonly diagnosed cancers [3,4]. While the overexpression of various proteins has been linked to the development and growth of cancer, VEGFR2 has emerged as a key therapeutic target due to its role in the formation of new blood vessels as well as the regulation of endothelial differentiation of colon cancer cells. Moreover, VEGFR2 inhibition has been found to hamper breast cancer cell proliferation via enhanced mitochondrial biogenesis.

Despite the availability of approved therapy for VEGFR2, there is a significant rise in multidrug resistance to the current drugs, necessitating the development of novel therapeutic options [5,6]. In order to accelerate drug discovery, several techniques have emerged, among which hybridization is one of the most frequently used. Molecular hybridization is a promising strategy that involves the combination of two or more pharmacophoric moieties in a single molecular architecture with improved activity and affinity with reference to their parent molecules [7]. It has been employed in the design of anticancer drugs by linking one anticancer drug with another anticancer drug or a carrier molecule strategically. This results in increased selectivity, fewer side effects, reduction in drug resistance, better patient compliance, and more biological potency [8,9]. 

**Figure 1:**
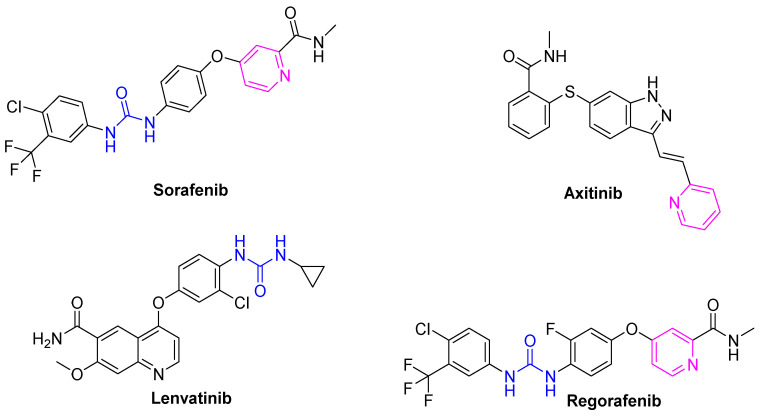
Chemical structures of FDA-approved VEGFR-2 inhibitors.

Most of the drugs that have been developed to combat cancer and its spread possess heterocycles in their backbone with non-fused pyridines emerging as one of the main building blocks for the development of anticancer drugs (Figure 1) [10,11,12]. Consequently, many of the drugs developed to combat cancer share the common feature of heterocyclics in their backbone, with non-fused pyridines playing a prominent role [13,14]. This class of heterocycles is known to possess diverse biological activities, particularly anticancer activities.

Several pyridine-based small molecule VEGFR2 inhibitors have been approved as anticancer drugs, such as sorafenib, axitinib, and regorafenib (Figure 1) [13,15,16]. These compounds also share the presence of a urea or amide linker (urido), with the urea moiety being an essential pharmacophoric feature in several anticancer drugs such as sorafenib and regorafenib [17]. With consideration of these pharmacophoric features, a hybridization approach was adopted in this study, utilizing pyridine–urea as the foundational core for the development of novel hybrid compounds.

Quinazolines are versatile heterocyclic compounds that have shown significant promise in anticancer research due to their unique chemical structure and potent cytotoxic activity against various cancer types [18,19]. Several compounds derived from quinazoline have gained approval for the treatment of various conditions, including benign prostatic hyperplasia, posttraumatic stress disorder, as well as lung and pancreatic cancers {Jafari, 2016 #115}. Based on the successful incorporation of the quinazoline ring in previous studies, as well as the compelling evidence supporting its efficacy, the present investigation chose to incorporate the quinazoline ring as the “head” moiety in the new scaffold attached to the pyridine ring [20].

**Figure 2:**
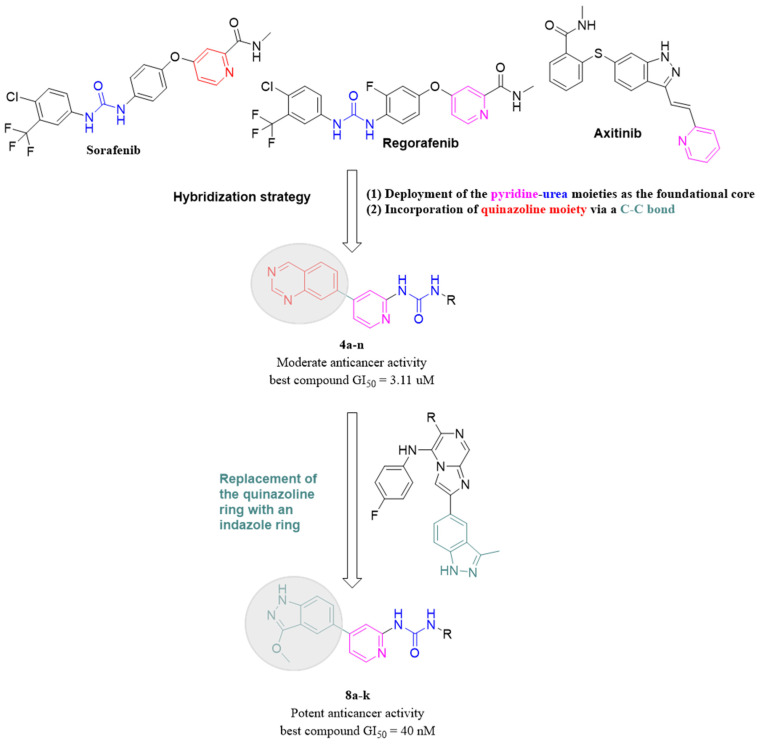
The design and optimization strategy employed to yield the target compounds.

While various aliphatic, cyclic, and aromatic substitutions were used as the “tail” moiety (Figure 2). Unfortunately, the quinazoline-based hybrids did not exhibit satisfactory anticancer activity against breast and colon cancer cell lines, likely due to the bulky nature of the quinazoline ring. Predicting that the bulky nature of the quinazoline ring was the cause of the unsatisfactory anticancer activity, the quinazoline moiety was replaced with a less bulky one. Inspired by a recent study that reported the successful replacement of a benzene ring with an indazole to produce new anticancer derivatives and given that the indazole and quinazoline moieties contain the same number of nitrogen atoms, the quinazoline ring was replaced with an indazole one in the new hybrids (Figure 2) [21]. This substitution led to a significant increase in cytotoxic activity against both breast and colon cancer cell lines.

The synthesized compounds were evaluated for their cytotoxic activity against HCT-116 and MCF-7 cancer cell lines, a method that has been employed to assess the cytotoxic activity of various VEGFR2 inhibitors [22,23]. Furthermore, the most active compounds were subjected to molecular docking and molecular dynamics (MD) studies as well as free energy calculations to ensure their binding affinity toward VEGFR2.

## 2. Results and Discussions

### 2.1. Chemistry

**Scheme 1:**
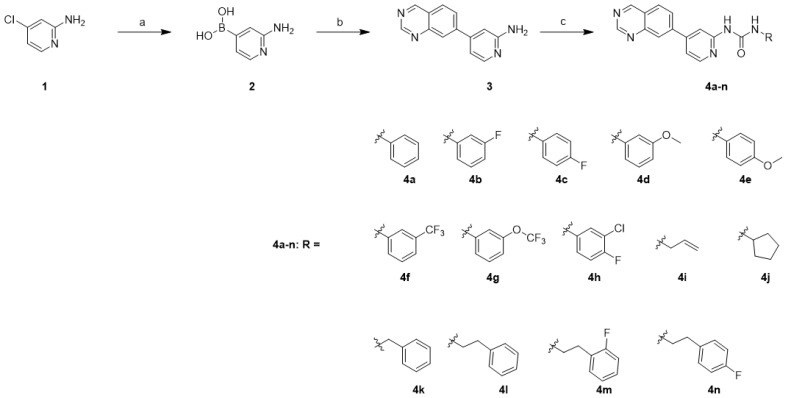
Synthesis of quinazoline derivatives **4a**–**n*^a^*.** Reagents and conditions: (**a**) bis(pinacolato)diboron, Sphos, Pd_2_(dba)_3_, potassium acetate, 1,4-dioxane, 100 °C, 12 h, quant.; (**b**) 6-bromoquinazoline, Pd(dppf)Cl_2_∙DCM, Cs_2_CO_3_, 1,4-dioxane/H_2_O (9/1), 110 °C, 12 h, 12%; (**c**) RNCO, DIPEA, THF, 60 °C, 12 h, 18–68%.

The synthesis of compounds **4a**–**n** was carried out as illustrated in Scheme 1. The palladium-catalyzed coupling reaction of bis(pinacolato)diboron with commercially available 4-chloropyridin-2-amine (**1**) in the presence of potassium acetate and SPhos afforded boronic acid **2**. The synthesis of boronic acid was afforded by following the reported procedure [24,25]. The installation of quinazoline was achieved using the Suzuki cross-coupling reaction of boronic acid **2** with 6-bromoquinazoline in the presence of Pd(dppf)Cl_2_.DCM to afford the amine **3** [26]. Finally, the target compounds **4a**–**n** were synthesized by treating the precursor amine **3** with the corresponding isocyanate in the presence of *N*,*N*-diisopropylethylamine (DIPEA).

3-Methoxy-1*H*-indazole derivatives **8a**–**k** were synthesized using a similar approach with a slightly modified reaction order and procedure in the first and last steps, as shown.

**Scheme 2:**
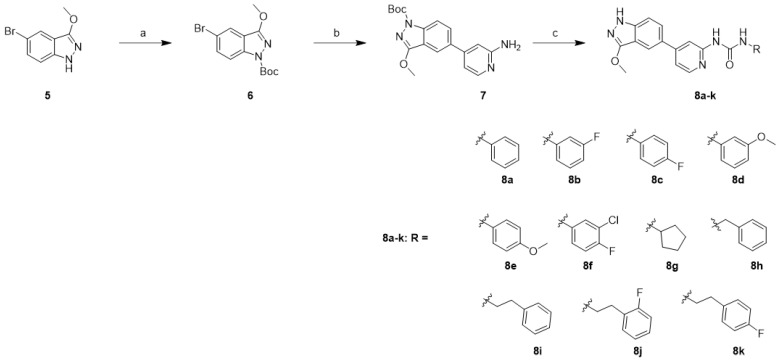
Synthesis of 3-methoxy-1*H*-indazole derivatives **8a**–**k:** reagents and conditions: (**a**) (Boc)_2_O, Et_3_N, THF, rt, 18 h, 96%; (**b**) (2-aminopyridin-4-yl)boronic acid (**2**), Xphos-pd-G2, K_2_CO_3_, 1,4-dioxane/H_2_O (9/1), 110 °C, 2 h, 30%; (**c**) (i) RNCO, DIPEA, THF, 60 °C, 12 h; (ii) 4 N HCl in 1,4-dioxane, MeOH, rt, 2 h, 14–40%.

In Scheme 2, the synthesis started with the protection of 5-bromo-3-methoxy-1*H*-indazole (**5**) with Boc anhydride in the presence of triethylamine (Et_3_N) to afford **6**. Unfortunately, the Suzuki coupling methodology employed in Scheme 1 failed to afford compound **7**. Accordingly, Xphos-pd-G2 and K_2_CO_3_ was used. The Boc-protected indazole **6** was converted to **7** using a Suzuki cross-coupling reaction with (2-aminopyridin-4-yl)boronic acid (**2**). The amine in **7** was treated using the appropriate isocyanate in the presence of *N*,*N*-diisopropylethylamine (DIPEA) followed by removal of the Boc-protecting group with 4 N HCl, affording compounds **8a**–**k**.

### 2.2. Biological Evaluation

To evaluate the anticancer activity of the synthesized compounds, the inhibitory effect of the synthesized compounds on cell proliferation in the form of GI_50_ was measured and compared to the positive control drug, irinotecan. Irinotecan (Supporting Information, Figure S68) was chosen as the standard drug, as it is a widely used FDA-approved chemotherapy drug for treating various types of cancer, including colorectal cancer, small cell lung cancer, pancreatic cancer, and gastric cancer, among others. For this study, the HCT116 and MCF7 cancer cell lines were selected due to their well-established nature and extensive use in evaluating various breast and colon cancer candidates. Furthermore, it was discovered that VEGFR-2 plays a critical role in cell survival and regulates endothelial differentiation in both MCF-7 breast cancer cells and HCT-116 human colorectal carcinoma cells [27,28,29,30].

The results showed that irinotecan exhibited GI_50_ values of 0.35 ± 0.069 and 0.62 ± 0.003 μM against the MCF7 and HCT116 cancer cell lines, respectively. Generally, the quinazoline-based compounds (**4a**–**n**) exhibited lower cytotoxic activity compared to the 3-methoxy-1*H*-indazole compounds (**8a**–**k**). Among the synthesized quinazoline-based compounds, only compounds **4i**, **4k**, and **4l** showed a strong inhibitory effect (Table 1) on the breast cancer cell line (MCF7) with GI_50_ values of 3.35 ± 0.101, 3.03 ± 0.061, and 9.00 ± 0.347 μM, respectively. However, these three compounds did not exhibit any potent effect on the colon cancer cell line, HCT116, indicating that the quinazoline-based compounds are more specific to breast cancer.

On the other hand, with the exception of 3-fluoro-substituted derivative (compound **8b**), the unsubstituted 3-methoxy-1*H*-indazole derivatives, namely compounds **8a**, **8h**, and **8i**, displayed the most potent activity against both MCF7 and HCT116 cancer cell lines. Compound **8a** exhibited GI_50_ values of 0.06 ± 0.014 μM and 1.19 ± 0.035 μM against MCF7 and HCT116 cell lines, respectively. This result means that compound **8a** displayed 6-fold potency when compared to the positive control drug Irinotecan against the MCF7 cancer cell line. Compounds **8h** and **8i** exhibited GI_50_ values of 0.23 ± 0.015 μM and 0.16 ± 0.013 μM against the MCF7 cancer cell lines (Table 1). Furthermore, compound **8h** showed a GI_50_ value of 0.33 ± 0.042 μM against HCT116 cell line, which is twice the potency displayed by the FDA-approved drug irinotecan against the HCT116 cancer cell line (GI_50_ = 0.62 ± 0.003). Overall, the 3-methoxy-1*H*-indazole compounds demonstrated potent cytotoxic activity against both MCF7 and HCT116 cell lines, particularly the unsubstituted derivatives **8a**, **8h**, and **8i**. These findings suggest that 3-methoxy-1*H*-indazole compounds could be promising candidates for the development of novel anticancer drugs targeting breast and colon cancers.

**Figure 3:**
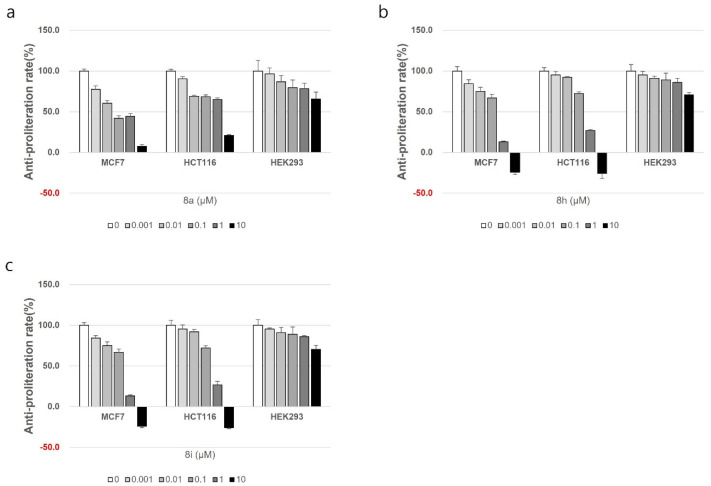
The cytotoxic effect of compounds **8a** (**a**), **8h** (**b**), and **8i** (**c**) on the normal cell line HEK293.

In order to assess the safety profile of the synthesized compounds, the compounds with the best cytotoxic activity (compounds were **8a**, **8h**, and **8i**) were tested against the normal cell line, HEK293. In HEK293 cells, it was confirmed that the compounds exhibited lower cell growth inhibitory effects than the anticancer effects shown in MCF and HCT116, where the cell growth rate did not decrease by less than 50% compared to the control group as illustrated in Figure 3.

### 2.3. Molecular Docking

Molecular docking plays a significant role in computer-aided drug design and has diverse applications. One such application involves predicting the binding modes between a ligand and its target, enabling the ranking of compounds based on their docking scores and establishing a relationship between these scores and potential activity [32]. The visualization of interactions obtained from docking studies can further assist in improving the affinity characteristics of the ligands under investigation [33]. Consequently, a thorough molecular docking analysis was conducted to assess the binding affinity of the best cytotoxic compounds, compounds **8a**, **8h**, and **8i**, towards VEGFR2.

**Figure 4:**
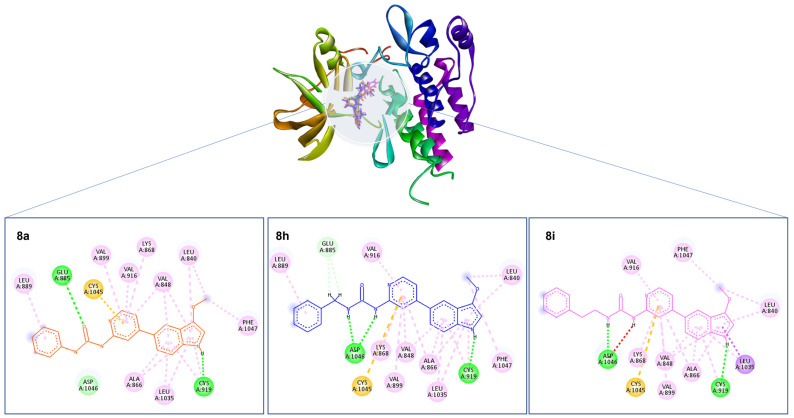
Depiction of the 3D binding orientation of compounds **8a** (orange), **8h** (blue), and **8i** (magenta) overlayed within the binding cavity of VEGFR2 and their corresponding 2D interactions established with the binding cavity’s amino acid residues. Favorable interactions represented as dashed lines: green—hydrogen bonds; yellow—π–sulfur; dark pink—π–π stacking interactions; light pink—hydrophobic interactions; turquoise—halogen interaction.

The active site of VEGFR-2 features straightforward architecture consisting of a front pocket and a back pocket. The front pocket, responsible for ATP binding, is influenced by two critical residues, namely Glu917 and Cys919. On the other hand, the back pocket, which is hydrophobic in nature, includes Glu885 and Asp1046. Glu885 is positioned along the αC helix, while Asp1046 forms an integral component of the triad [34]. The compounds displayed a similar binding pattern withing the binding cavity highlighted by occupying the same position within the binding site. Moreover, all three compounds established two hydrogen bonds with Glu885 and Cys919 amino acid residues of the binding cavity. Moreover, the compounds shared a common π–sulfur interaction with Cys1045 amino acid residues (Figure 4). Interestingly, in the crystallized ligand, Tivozanib established three hydrogen bonds with Glu885, Cys919, and Asp1046, which further validates the ability of the tested compounds as VEGFR2 inhibitors.

### 2.4. MD Simulations

The synthesized compounds exhibited potent cytotoxic activity and demonstrated favorable interactions as revealed by molecular docking studies. However, it is essential to acknowledge that this method solely considers the flexibility of ligand conformations while keeping the protein structure rigid. Moreover, MD simulation plays a critical role in assessing theoretical accuracy, necessitating significant computational resources. Various theoretical methods are employed during MD analysis to select conformations from simulations, enabling the identification of relevant molecular conformations and aiding in the interpretation of simulation data [35,36]. To evaluate the stability of the binding poses and assess the dynamics of the protein conformation, molecular dynamics (MD) simulations were conducted for 100 ns on protein–ligand complexes involving compounds **8a**, **8h**, and **8i**.

The stability of the protein–ligand complexes and the reliability of the simulation protocol were evaluated by calculating the root mean square deviation (RMSD). The RMSD values provide a quantitative measure of the structural similarity between the initial and final states of the protein–ligand complexes, enabling an assessment of the stability of these complexes throughout the simulation [37]. Moreover, by comparing the simulation results with the unbound state of the protein and a known complex, the validity of the simulation protocol can be assessed [38]. This approach offers a more accurate understanding of the binding interactions and the stability of the complexes, accounting for the dynamic behavior of the protein and the ligand in solution.

After initial fluctuations in the first 50 ns, the complexes of compounds **8a**, **8h**, and **8i** displayed a similar stability profile with average RMSD of 2.1 Å, which is well below the accepted range of 3 Å, indicating the formation of stable complexes with VEGFR2. Moreover, the ligands in all three complexes showed RMSD of less than 1 Å (raw data file), indicating stability throughout the simulation and a proper fit inside the binding cavity (Supporting Information, Figure S70).

The affinity of a molecule for a binding site is greatly influenced by its capacity to establish and sustain hydrogen bonds with residues within the binding site [39]. To further evaluate the stability of ligand binding modes, an examination of the intermolecular hydrogen bonds formed within the protein–ligand complexes was conducted. Hydrogen bond analysis revealed that only compound **8a** was able to maintain a hydrogen bond with Cys919 amino acid residue of the hinge area, which is reported to be a crucial hydrogen bond formation with the adenine ring of ATP in the front pocket. The hydrogen bond between compound **8a** and Cys919 was maintained for more than 50% of the simulation time, indicating its stability. Meanwhile, all three compounds were able to establish stable hydrogen bonds with Glu885 (located in the αC-helix) and Asp1046 (which is a part of the DFG motif) indicating a shared binding mode of action against the hydrophobic back pocket, which is formed by the DFG-out conformation (Supporting Information, Figure S71) [40,41,42,43]. Compound **8h** maintained the hydrogen bonds with Glu885 and Asp1046 for around 60% of the simulation time. Meanwhile, compound **8i** was able to maintain the same hydrogen bonds for almost 100% of the simulation time, indicating greater stability. The ability to consistently maintain stable hydrogen bonds throughout the simulations confirms that the compounds share a common binding mode. Additionally, it highlights the crucial role played by these particular amino acid residues in facilitating the binding of inhibitors to VEGFR2. This finding aligns with previous reports that have also emphasized the essentiality of these amino acids in the binding process [44,45].

### 2.5. MM–GBSA

The MM–GBSA (molecular mechanics–generalized Born surface area) method is a robust and widely utilized approach for predicting binding free energy (ΔGBind) after conducting simulations, considering protein flexibility, entropy, and polarizability [46]. It effectively identifies ligands that exhibit efficient binding to receptors and plays a vital role in biomolecular research for comprehending molecular activities [47]. To validate the compounds identified using docking and molecular dynamics simulations, calculations of MM–GBSA binding free energy were employed. At intervals of 10 frames from frame 0 to 1001, post-simulation MM–GBSA was computed, resulting in a total of 100 conformations for each simulated complex. The MM–GBSA binding energy statistics provided in Table 2 indicate the average cumulative contributions of coulombic, hydrogen bonding, solvation energy, and lipophilic interactions, all of which exert a significant influence on ΔGBind.

Out of the three complexes under investigation, it was observed that the complex formed by compound **8i** exhibited the highest binding free energy (ΔGBind) with a value of −59.55 kcal/mol. This superior binding affinity can be attributed to its notable ability to establish both hydrogen bonds and lipophilic interactions, surpassing the complexes formed by the other two compounds. Notably, throughout the entire duration of the 100% MD simulation, compound **8i** consistently maintained its hydrogen bonds, as depicted in Figure S71 as shown in Supporting Information. This persistence in forming and preserving hydrogen bonds further reinforces the prediction that compound **8i** exhibits the most potent activity towards VEGFR2. Moreover, compound **8i**, which exhibited the highest binding free energy, was the most potent compound against the MCF-7 cell line, indicating the presence of a correlation between the free binding energy and the cytotoxicity of the compounds.

### 2.6. In Silico Pharmacokinetic Study

In the development of active therapeutic agents, pharmacokinetic properties such as absorption, metabolism, excretion, and toxicity (ADMET) play a critical role. It is important to note that a successful antagonistic interaction of inhibitors with a receptor protein or enzyme does not guarantee their suitability as drugs [48]. The presence of poor ADME characteristics and unfavorable toxicology are common reasons for the failure of drug candidates in clinical experiments [49]. Consequently, conducting ADME analysis is vital in the process of drug development. The freely accessible web server SwissADME was utilized to predict the pharmacokinetic properties of the most active compounds (compounds **8a**, **8h**, and **8i**). This machine learning platform employs graph-based signatures encoded as distance/pharmacophore patterns to predict small-molecule pharmacokinetic properties [50].

According to the calculations, all three compounds (Table 3) were found to be water-soluble and demonstrated high gastrointestinal (GI) absorption. Additionally, they were found to comply with Lipinski’s rule, suggesting their potential for oral activity. Furthermore, the predictions indicated that none of the three compounds were able to penetrate the blood–brain barrier (BBB), indicating a low likelihood of causing central nervous system (CNS) side effects.

## 3. Conclusions

In this study, rational drug design was employed to design a novel series of pyridine–urea hybrid compounds. Using a stepwise optimization process, a group of 3-methoxy-1*H*-indazoles that possesses potent anticancer activity was identified. These novel compounds demonstrated significant efficacy against both breast (MCF7) and colon (HCT116) cancer cell lines. Among the synthesized compounds, compounds **8a**, **8h**, and **8i** exhibited the highest cytotoxicity against both MCF7 and HCT116 cell lines. Compound **8a** demonstrated exceptional potency against MCF7 cells, with a GI_50_ value of 0.06 ± 0.014 μM. This GI_50_ value indicates around sixfold greater potency compared to the positive control drug irinotecan. Furthermore, when tested on normal cells, compounds **8a**, **8h**, and **8i** exhibited low cytotoxicity, indicating a promising safety profile. In silico investigation of the compounds predicted their action as VEGFR2 inhibitors and explained their mechanism of action. This information provides valuable insights into their mode of action and further supports their potential as anticancer agents.

## 4. Methodology

### 4.1. Chemistry

General Procedures: The commercial chemicals, reagents, isocyanates, and solvents used were purchased from Sigma-Aldrich (Seoul, Republic of Korea), TCI (Seoul, Republic of Korea), Alfa Aesar (Seoul, Republic of Korea), and U CHEM (Seoul, Republic of Korea). All reactions were carried out in oven-dried glassware under a nitrogen atmosphere. Reactions were monitored using analytical thin-layer chromatography on 0.25 mm silica plates (E. Merck; silica gel 60 F254). Flash column chromatography was performed on silica gel 60 (230–400 mesh Kieselgel 60) using a Biotage system. The mass spectra were recorded using high-resolution mass spectrometry (HRMS) (electron ionization MS) on a Waters G2 QTOF mass spectrometer. The chemical shifts were reported in parts per million (ppm) and the coupling constants in hertz (Hz) (proton magnetic resonance spectra were recorded using a Varian 400 MHz spectrometer and carbon magnetic resonance spectra were recorded using a Varian 100 MHz spectrometer (Varian Medical Systems, Inc., Palo Alto, CA, USA)). RP-HPLC data analysis of the final products was performed using a Waters Corp. HPLC system equipped with an ultraviolet (UV) detector set at 254 nm and a YMC C18 (HS12S05-1564WT) column (5 μM, 12 nm) that was 4.6 mm × 150 mm in size was also employed. The RP-HPLC mobile phases used were (A) water containing 0.05% TFA and (B) acetonitrile. The purity of compounds was assessed using a gradient of 5% to 100% of acetonitrile in 40 min (method A) or a gradient of 5% to 60% of acetonitrile in 40 min (method B) with a flow rate of 1.0 mL/min. Melting points were measured using a Thermo Scientific 9200 melting point apparatus. For all biologically tested compounds, the purity was >95%. The spectral data for all synthesized compounds can be found in the Supplementary Materials.

(2-Aminopyridin-4-yl)boronic acid (**2**)

To a solution of 4-chloropyridin-2-amine (**1**, which was purchased and used directly) (10.0 g, 77.7 mmol) in 1,4-dioxane (200 mL), bis(pinacolato)diboron (23.7 g, 93.3 mmol) and potassium acetate (12.9 g, 132.2 mmol) were added. The mixture was degassed using nitrogen for 5 min, followed by the addition of Pd_2_(dba)_3_ (0.71 g, 0.77 mmol) and SPhos (1.27 g, 3.11 mmol). The resulting reaction mixture was stirred at 100 °C for 3 h in a sealed tube. After cooling to room temperature, the mixture was diluted using EtOAc (150 mL). The solid precipitate was filtered through a Celite pad. The filtrate was evaporated, and the obtained crude residue was precipitated with diethyl ether to afford (2-aminopyridin-4-yl)boronic acid as a white solid (18.0 g, quant.) and used in the next reaction step without further purification. ^1^H NMR (400 MHz, DMSO-*d*_6_): *δ* 7.88 (d, *J* = 4.8 Hz, 1H), 6.73 (s, 1H), 6.61 (d, *J* = 4.9 Hz, 1H), 5.88 (s, 2H), 3.55 (s, 2H); HRMS (ESI): [M + H]^+^ calcd for C_5_H_8_BN_2_O_2_ 139.0679, found 139.0679.

4-(Quinazolin-7-yl)pyridin-2-amine (**3**)

To a degassed solution of 6-bromoquinazoline (2.00 g, 9.57 mmol), (2-aminopyridin-4-yl)boronic acid (**2**) (1.32 g, 9.57 mmol), and K_2_CO_3_ (3.97 g, 28.7 mmol) in 1,4-dioxane/H_2_O (70 mL, 9/1), Pd(dppf)Cl2∙DCM (0.78 g, 0.96 mmol) was added. The resulting mixture was stirred at 100 °C for 2 h in a sealed tube. After cooling to room temperature, the mixture was diluted with water (40 mL) and extracted with ethyl acetate (3 × 50 mL). The combined organic layers were dried over anhydrous sodium sulfate, filtered, and evaporated. The crude residue was purified using silica gel column chromatography (5–10% MeOH in DCM) to afford 4-(quinazolin-7-yl)pyridin-2-amine (**3**) as a brown solid (0.25 g, 12%). ^1^H NMR (400 MHz, DMSO-*d*_6_): *δ* 9.65 (s, 1H), 9.34 (s, 1H), 8.27 (d, *J* = 8.6 Hz, 1H), 8.21 (s, 1H), 8.04 (dd, *J* = 12.3, 3.6 Hz, 2H), 6.97 (d, *J* = 4.2 Hz, 1H), 6.88 (s, 1H), 6.12 (s, 2H); ^13^C NMR (101 MHz, Chloroform-*d*) *δ* 160.0, 155.9, 149.2, 148.63 131.3, 131.2, 128.3, 127.9, 127.0, 126.4, 124.7, 112.8, 106.6. HRMS (ESI): [M + H]^+^ calcd for C_13_H_11_N_4_ 223.0984, found 223.0983.

*tert*-Butyl 5-bromo-3-methoxy-1H-indazole-1-carboxylate (**6**)

To a solution of 5-bromo-3-methoxy-1*H*-indazole (**5**) (2.40 g, 10.5 mmol) in THF (25 mL), triethylamine (4.42 mL, 31.6 mmol) and Boc-anhydride (4.61 g, 21.1 mmol) were added at room temperature. The reaction mixture was stirred at room temperature for 18 h. After cooling to room temperature, the mixture was diluted using water (40 mL) and extracted using ethyl acetate (3 × 50 mL). The combined organic layers were dried over anhydrous sodium sulfate, filtered, and evaporated. The crude residue was purified using silica gel column chromatography (10% EtOAc in hexanes) to afford *tert*-butyl 5-bromo-3-methoxy-1*H*-indazole-1-carboxylate (**6**) as an off-white solid (3.30 g, 96%). ^1^H NMR (400 MHz, Chloroform-*d*): *δ* 7.85 (s, 1H), 7.80 (d, *J* = 1.7 Hz, 1H), 7.59 (dd, *J* = 8.9, 1.7 Hz, 1H), 4.17 (s, 3H), 1.70 (s, 9H); ^13^C NMR (101 MHz, DMSO-*d*_6_) *δ* 158.3, 148.6, 139.8, 133.3, 122.5, 118.1, 116.7, 115.9, 84.6, 57.0, 28.1. HRMS (ESI): [M + H]^+^ calcd for C_13_H_16_BrN_2_O_3_ 327.0344, mass was not observed.

*tert*-Butyl 5-(2-aminopyridin-4-yl)-3-methoxy-1H-indazole-1-carboxylate (**7**)

To a degassed solution of *tert*-butyl 5-bromo-3-methoxy-1*H*-indazole-1-carboxylate (**6**) (3.40 g, 10.3 mmol), (2-aminopyridin-4-yl)boronic acid (1.71 g, 12.46 mmol), and K_2_CO_3_ (4.30 g, 31.1 mmol) in 1,4-dioxane/H_2_O (70 mL, 9/1), Xphos-Pd-G2 (0.818 g, 1.03 mmol) was added. The resulting mixture was stirred at 100 °C for 2 h in a sealed tube. After cooling to room temperature, the mixture was diluted using water (40 mL) and extracted using ethyl acetate (3 × 50 mL). The combined organic layers were dried over anhydrous sodium sulfate, filtered, and evaporated. The crude residue was purified using silica gel column chromatography (5–10% MeOH in DCM) to afford *tert*-butyl 5-(2-aminopyridin-4-yl)-3-methoxy-1*H*-indazole-1-carboxylate (**7**) as an off-white solid (1.05 g, 30%). ^1^H NMR (400 MHz, DMSO-*d*_6_): *δ* 8.07 (d, *J* = 9.1 Hz, 1H), 7.98 (d, *J* = 5.4 Hz, 1H), 7.91 (d, *J* = 8.1 Hz, 2H), 6.87 (d, *J* = 5.4 Hz, 1H), 6.78 (s, 1H), 6.00 (s, 2H), 4.11 (s, 3H), 1.65 (s, 9H); ^13^C NMR (101 MHz, DMSO-*d*_6_) *δ* 160.8, 159.6, 149.0, 147.6, 134.3, 129.2, 117.7, 117.1, 115.5, 110.5, 105.5, 84.4, 73.9, 57.0, 28.2, 25.3; HRMS (ESI): [M + H]^+^ calcd for C_18_H_21_N_4_O_3_ 341.1614, found 341.1613.

General Procedure for the Conversion of Amine to Urea (Method A).

To a solution of amine (0.44 mmol) and appropriate isocyanate (0.53 mmol) in THF (10 mL) at room temperature, DIPEA (1.34 mmol) was added. The resulting reaction mixture was heated at 70 °C for 12 h. After cooling to room temperature, the excess of solvent was evaporated. The crude residue was purified using silica gel column chromatography (0–5% MeOH in DCM).

1-Phenyl-3-(4-(quinazolin-7-yl)pyridin-2-yl)urea (**4a**)

This compound was prepared from 4-(quinazolin-7-yl)pyridin-2-amine (**3**) and phenyl isocyanate according to the general procedure for the conversion of amine to urea (method A) as an off-white solid (34.2 mg, 45%). Mp: 332–335 °C. ^1^H NMR (400 MHz, DMSO-*d*_6_): *δ* 10.28 (s, 1H), 9.68 (s, 1H), 9.54 (s, 1H), 9.36 (s, 1H), 8.43 (d, *J* = 5.3 Hz, 1H), 8.36–8.28 (m, 2H), 8.09 (dd, *J* = 8.6, 1.4 Hz, 1H), 8.03 (s, 1H), 7.59–7.48 (m, 3H), 7.30 (t, *J* = 7.8 Hz, 2H), 7.01 (t, *J* = 7.3 Hz, 1H); ^13^C NMR (101 MHz, DMSO-*d*_6_): *δ* 161.1, 156.2, 154.1, 152.6, 149.9, 148.6, 148.4, 143.7, 139.4, 129.5, 129.3, 127.2, 126.0, 125.0, 123.0, 119.2, 116.5, 110.3; HRMS (ESI): [M + H]^+^ calcd for C_20_H_16_N_5_O 342.1355, mass was not observed; RP-HPLC purity ≥ 94.5%, *t*_R_ = 13.2 min (method A).

1-(3-Fluorophenyl)-3-(4-(quinazolin-7-yl)pyridin-2-yl)urea (**4b**)

This compound was prepared from 4-(quinazolin-7-yl)pyridin-2-amine (**3**) and 3-fluorophenyl isocyanate according to the general procedure for the conversion of amine to urea (method A) as an off-white solid (16.3 mg, 20%). Mp: 322–325 °C. ^1^H NMR (400 MHz, DMSO-*d*_6_): *δ* 10.53 (s, 1H), 9.68 (d, *J* = 13.7 Hz, 2H), 9.39 (s, 1H), 8.46 (s, 1H), 8.34 (s, 1H), 8.08 (d, *J* = 32.0 Hz, 2H), 7.59 (s, 1H), 7.34 (s, 1H), 7.22 (s, 1H), 6.85 (s, 1H), 6.69 (s, 1H), 5.32 (s, 1H); ^13^C NMR (101 MHz, DMSO-*d*_6_): *δ* 161.8, 161.1, 156.2, 148.5, 147.0, 146.2, 145.6, 144.6, 143.6, 130.0, 129.4, 127.2, 126.1, 125.0, 122.2, 116.7, 110.0, 106.1, 99.8, 95.4; ^19^F NMR (376 MHz, Chloroform-*d*): *δ* −110.7; HRMS (ESI): [M + H]^+^ calcd for C_20_H_15_FN_5_O 360.1261, mass was not observed; RP-HPLC purity ≥ 96.5%, *t*_R_ = 15.1 min (method A).

1-(4-Fluorophenyl)-3-(4-(quinazolin-7-yl)pyridin-2-yl)urea (**4c**)

This compound was prepared from 4-(quinazolin-7-yl)pyridin-2-amine (**3**) and 4-fluorophenyl isocyanate according to the general procedure for the conversion of amine to urea (method A) as an off-white solid (34.4 mg, 43%). Mp: 310–313 °C. ^1^H NMR (400 MHz, DMSO-*d*_6_): *δ* 10.37 (s, 1H), 9.71 (s, 1H), 9.58 (s, 1H), 9.39 (s, 1H), 8.45 (d, *J* = 5.3 Hz, 1H), 8.12 (dd, *J* = 8.4, 1.8 Hz, 1H), 8.03 (s, 1H), 7.62–7.51 (m, 3H), 7.17 (t, *J* = 8.7 Hz, 2H); ^13^C NMR (101 MHz, DMSO-*d*_6_): *δ* 161.2, 156.2, 154.1, 152.7, 149.9, 148.5, 148.4, 143.7, 135.7, 129.5, 127.2, 126.0, 125.0, 121.1, 116.5, 115.9, 115.7, 110.3; HRMS (ESI): [M + H]^+^ calcd for C_20_H_15_FN_5_O 360.1261, mass was not observed; RP-HPLC purity ≥ 96.2%, *t*_R_ = 13.4 min (method A).

1-(3-Methoxyphenyl)-3-(4-(quinazolin-7-yl)pyridin-2-yl)urea (**4d**)

This compound was prepared from 4-(quinazolin-7-yl)pyridin-2-amine (**3**) and 3-methoxyphenyl isocyanate according to the general procedure for the conversion of amine to urea (method A) as an off-white solid (41.3 mg, 49%). Mp: 263–266 °C. ^1^H NMR (400 MHz, DMSO-*d*_6_): *δ* 10.27 (s, 1H), 9.68 (s, 1H), 9.53 (s, 1H), 9.36 (s, 1H), 8.42 (d, *J* = 5.3 Hz, 1H), 8.35–8.29 (m, 2H), 8.09 (dd, *J* = 8.5, 1.8 Hz, 1H), 8.02 (s, 1H), 7.54 (dd, *J* = 5.4, 1.7 Hz, 1H), 7.24 (t, *J* = 2.2 Hz, 1H), 7.19 (t, *J* = 8.1 Hz, 1H), 7.00 (d, *J* = 8.5 Hz, 1H), 6.59 (dd, *J* = 8.2, 2.4 Hz, 1H), 3.72 (s, 3H); ^13^C NMR (101 MHz, DMSO-*d*_6_): *δ* 161.1, 160.2, 156.2, 154.1, 152.5, 149.9, 148.6, 148.4, 143.7, 140.6, 130.1, 129.4, 127.2, 126.0, 125.0, 116.5, 111.5, 110.2, 108.4, 105.0, 55.4; HRMS (ESI): [M + H]^+^ calcd for C_21_H_18_N_5_O_2_ 372.1460, mass was not observed; RP-HPLC purity ≥ 99.7%, *t*_R_ = 13.8 min (method A).

1-(4-Methoxyphenyl)-3-(4-(quinazolin-7-yl)pyridin-2-yl)urea (**4e**)

This compound was prepared from 4-(quinazolin-7-yl)pyridin-2-amine (**3**) and 4-methoxyphenyl isocyanate according to the general procedure for the conversion of amine to urea (method A) as an off-white solid (46.1 mg, 55%). Mp: 283–286 °C. ^1^H NMR (400 MHz, DMSO-*d*_6_): *δ* 10.17 (s, 1H), 9.68 (s, 1H), 9.50 (s, 1H), 9.36 (s, 1H), 8.41 (d, *J* = 5.4 Hz, 1H), 8.34–8.29 (m, 2H), 8.08 (d, *J* = 8.8 Hz, 1H), 7.98 (s, 1H), 7.52 (d, *J* = 5.8 Hz, 1H), 7.45–7.40 (m, 2H), 6.88 (d, *J* = 8.8 Hz, 2H), 3.70 (s, 3H); ^13^C NMR (101 MHz, DMSO-*d*_6_): *δ* 161.1, 156.2, 155.4, 154.3, 152.7, 149.9, 148.5, 148.3, 143.7, 132.3, 129.4, 127.1, 126.0, 125.0, 121.0, 116.3, 114.5, 110.2, 55.6; HRMS (ESI): [M + H]^+^ calcd for C_21_H_18_N_5_O_2_ 372.1460, mass was not observed; RP-HPLC purity ≥ 95.4%, *t*_R_ = 12.7 min (method A).

1-(4-(Quinazolin-7-yl)pyridin-2-yl)-3-(3-(trifluoromethyl)phenyl)urea (**4f**)

This compound was prepared from 4-(quinazolin-7-yl)pyridin-2-amine (**3**) and 3-(trifluoromethyl)phenyl isocyanate according to the general procedure for the conversion of amine to urea (method A) as an off-white solid (33.4 mg, 36%). Mp: 328–331 °C. ^1^H NMR (400 MHz, DMSO-*d*_6_): *δ* 9.72 (s, 1H), 9.40 (s, 1H), 8.58 (s, 1H), 8.54 (d, *J* = 7.6 Hz, 1H), 8.38–8.30 (m, 3H), 7.83 (d, *J* = 9.2 Hz, 2H), 7.80–7.73 (m, 3H), 7.71 (d, *J* = 8.0 Hz, 1H), 7.61 (dd, *J* = 7.6, 2.0 Hz, 1H); ^13^C NMR (101 MHz, DMSO-*d*_6_): *δ* 161.3, 156.4, 154.9, 153.1, 151.1, 149.8, 148.7, 140.7, 137.0, 133.3, 130.8, 130.3, 129.5, 127.4, 127.0, 125.8, 125.6, 122.9, 120.9, 112.4, 110.0; ^19^F NMR (376 MHz, Chloroform-*d*): *δ* −62.6; HRMS (ESI): [M + H]^+^ calcd for C_21_H_15_F_3_N_5_O 410.1229, mass was not observed; RP-HPLC purity ≥ 95.3%, *t*_R_ = 17.6 min (method A).

1-(4-(Quinazolin-7-yl)pyridin-2-yl)-3-(3-(trifluoromethoxy)phenyl)urea (**4g**)

This compound was prepared from 4-(quinazolin-7-yl)pyridin-2-amine (**3**) and 3-(trifluoromethoxy)phenyl isocyanate according to the general procedure for the conversion of amine to urea (method A) as an off-white solid (17.4 mg, 18%). Mp: 271–274 °C. ^1^H NMR (400 MHz, Chloroform-*d*): *δ* 12.00 (s, 1H), 9.50 (s, 1H), 9.42 (s, 1H), 8.44 (d, *J* = 5.4 Hz, 1H), 8.32 (s, 1H), 8.21 (s, 1H), 8.10 (d, *J* = 8.4 Hz, 1H), 7.93 (dd, *J* = 8.4, 1.7 Hz, 1H), 7.68 (s, 1H), 7.52 (d, *J* = 8.1 Hz, 1H), 7.38 (d, *J* = 8.2 Hz, 1H), 7.35 (dd, *J* = 5.4, 1.6 Hz, 1H), 7.17 (s, 1H), 6.97 (d, *J* = 8.3 Hz, 1H); ^13^C NMR (101 MHz, Chloroform-*d*): *δ* 160.2, 156.1, 153.3, 149.9, 147.0, 143.2, 139.9, 129.9, 128.3, 127.0, 126.6, 125.0, 122.6, 118.2, 116.2, 112.9, 110.1, 108.6, 29.7; ^19^F NMR (376 MHz, Chloroform-*d*): *δ* −57.6; HRMS (ESI): [M + H]^+^ calcd for C_21_H_15_F_3_N_5_O_2_ 426.1178, mass was not observed; RP-HPLC purity ≥ 95.4%, *t*_R_ = 26.6 min (method A).

1-(3-Chloro-4-fluorophenyl)-3-(4-(quinazolin-7-yl)pyridin-2-yl)urea (**4h**)

This compound was prepared from 4-(quinazolin-7-yl)pyridin-2-amine (**3**) and 3-chloro-4-fluorophenyl isocyanate according to the general procedure for the conversion of amine to urea (method A) as an off-white solid (44.4 mg, 50%). Mp: 318–321 °C. ^1^H NMR (400 MHz, DMSO-*d*_6_): *δ* 10.55 (s, 1H), 9.67 (d, *J* = 6.6 Hz, 2H), 9.36 (s, 1H), 8.43 (d, *J* = 5.4 Hz, 1H), 8.35–8.28 (m, 2H), 8.09 (dd, *J* = 8.5, 1.7 Hz, 1H), 7.98 (s, 1H), 7.90 (dd, *J* = 7.0, 2.2 Hz, 1H), 7.56 (d, *J* = 5.2 Hz, 1H), 7.37 (q, *J* = 9.3 Hz, 2H); ^13^C NMR (101 MHz, DMSO-*d*_6_): *δ* 161.1, 156.2, 153.9, 152.6, 149.9, 148.5, 143.6, 136.6, 136.6, 130.0, 129.5, 127.1, 126.1, 125.0, 120.6, 119.6, 117.5, 117.3, 116.6, 110.3; RP-HPLC purity ≥ 96.6%, *t*_R_ = 16.9 min (method A).

1-Allyl-3-(4-(quinazolin-7-yl)pyridin-2-yl)urea (**4i**)

This compound was prepared from 4-(quinazolin-7-yl)pyridin-2-amine (**3**) and allyl isocyanate according to the general procedure for the conversion of amine to urea (method A) as an off-white solid (26.4 mg, 38%). Mp: 190–193 °C. ^1^H NMR (400 MHz, DMSO-*d*_6_): *δ* 9.70 (s, 1H), 9.41 (d, *J* = 21.1 Hz, 2H), 8.34 (dd, *J* = 18.9, 9.5 Hz, 3H), 8.20 (s, 1H), 8.09 (d, *J* = 8.5 Hz, 1H), 7.90 (s, 1H), 7.49 (d, *J* = 5.1 Hz, 1H), 5.95–5.89 (m, 1H), 5.21 (d, *J* = 17.2 Hz, 1H), 5.11 (d, *J* = 10.4 Hz, 1H), 3.86 (s, 2H); ^13^C NMR (101 MHz, Chloroform-*d*): *δ* 160.1, 156.0, 156.0, 154.2, 150.2, 149.1, 147.0, 143.7, 135.0, 128.1, 126.8, 126.8, 124.9, 115.4, 115.3, 110.1, 42.3; HRMS (ESI): [M + H]^+^ calcd for C_17_H_16_N_5_O 306.1355, found 306.1357; RP-HPLC purity ≥ 99.7%, *t*_R_ = 11.4 min (method A).

1-Cyclopentyl-3-(4-(quinazolin-7-yl)pyridin-2-yl)urea (**4j**)

This compound was prepared from 4-(quinazolin-7-yl)pyridin-2-amine (**3**) and cyclopentyl isocyanate according to the general procedure for the conversion of amine to urea (method A) as an off-white solid (51.1 mg, 68%). Mp: 223–226 °C. ^1^H NMR (400 MHz, DMSO-*d*_6_): *δ* 9.69 (s, 1H), 9.37 (s, 1H), 9.19 (d, *J* = 4.1 Hz, 1H), 8.32 (d, *J* = 8.8 Hz, 2H), 8.27 (s, 1H), 8.07 (d, *J* = 8.3 Hz, 1H), 7.94 (s, 2H), 7.45 (d, *J* = 4.3 Hz, 1H), 4.04–3.98 (m, 1H), 1.91–1.84 (m, 2H), 1.68–1.54 (m, 4H), 1.45–1.38 (m, 2H); ^13^C NMR (101 MHz, DMSO-*d*_6_): *δ* 161.1, 156.2, 154.8, 154.6, 149.9, 148.4, 148.0, 143.8, 129.3, 127.1, 125.9, 125.0, 115.6, 109.8, 51.3, 33.3, 23.6; HRMS (ESI): [M + H]^+^ calcd for C_19_H_20_N_5_O 334.1668, found 334.1664; RP-HPLC purity ≥ 99.9%, *t*_R_ = 14.8 min (method B).

1-Benzyl-3-(4-(quinazolin-7-yl)pyridin-2-yl)urea (**4k**)

This compound was prepared from 4-(quinazolin-7-yl)pyridin-2-amine (**3**) and benzyl isocyanate according to the general procedure for the conversion of amine to urea (method A) as an off-white solid (42.9 mg, 54%). Mp: 221–224 °C. ^1^H NMR (400 MHz, DMSO-*d*_6_); *δ* 9.67 (s, 1H), 9.44 (s, 1H), 9.35 (s, 1H), 8.46 (s, 1H), 8.34–8.29 (m, 2H), 8.27 (s, 1H), 8.06 (dd, *J* = 8.4, 1.7 Hz, 1H), 7.90 (s, 1H), 7.45 (dd, *J* = 5.4, 1.7 Hz, 1H), 7.31 (d, *J* = 6.5 Hz, 4H), 7.25–7.21 (m, 1H), 4.40 (d, *J* = 5.9 Hz, 2H); ^13^C NMR (101 MHz, DMSO-*d*_6_): *δ* 161.1, 156.2, 155.3, 154.7, 149.9, 148.4, 148.2, 143.8, 140.3, 129.4, 128.8, 127.5, 127.2, 127.1, 125.9, 125.0, 115.8, 110.0, 43.1; HRMS (ESI): [M + H]^+^ calcd for C_21_H_18_N_5_O 356.1511, found 356.1511; RP-HPLC purity ≥ 99.7%, *t*_R_ = 16.1 min (method B).

1-Phenethyl-3-(4-(quinazolin-7-yl)pyridin-2-yl)urea (**4l**)

This compound was prepared from 4-(quinazolin-7-yl)pyridin-2-amine (**3**) and phenethyl isocyanate according to the general procedure for the conversion of amine to urea (method A) as an off-white solid (34.8 mg, 42%). Mp: 260–263 °C. ^1^H NMR (400 MHz, DMSO-*d*_6_): *δ* 9.69 (s, 1H), 9.38 (d, *J* = 10.1 Hz, 2H), 8.32 (d, *J* = 8.1 Hz, 1H), 8.27 (s, 2H), 8.18 (s, 1H), 8.06 (d, *J* = 8.1 Hz, 1H), 7.83 (s, 1H), 7.45 (s, 1H), 7.27 (t, *J* = 21.8 Hz, 5H), 3.46–3.41 (m, 2H), 2.84–2.77 (m, 2H); ^13^C NMR (101 MHz, DMSO-*d*_6_): *δ* 161.1, 156.2, 155.1, 154.7, 149.9, 148.1, 148.1, 143.8, 139.0; HRMS (ESI): [M + H]^+^ calcd for C_22_H_20_N_5_O 370.1668, found 370.1665; RP-HPLC purity ≥ 98.1%, *t*_R_ = 17.9 min (method B).

1-(2-Fluorophenethyl)-3-(4-(quinazolin-7-yl)pyridin-2-yl)urea (**4m**)

This compound was prepared from 4-(quinazolin-7-yl)pyridin-2-amine (**3**) and 1-fluoro-2-(2-isocyanatoethyl)benzene according to the general procedure for the conversion of amine to urea (method A) as an off-white solid (29.6 mg, 34%). Mp: 272–275 °C. ^1^H NMR (400 MHz, DMSO-*d*_6_): *δ* 9.67 (s, 1H), 9.35 (s, 2H), 8.30 (d, *J* = 8.5 Hz, 1H), 8.24 (d, *J* = 6.7 Hz, 2H), 8.17 (s, 1H), 8.06–8.02 (m, 1H), 7.81 (s, 1H), 7.44–7.41 (m, 1H), 7.35–7.31 (m, 1H), 7.25 (dd, *J* = 7.8, 5.6 Hz, 1H), 7.17–7.11 (m, 2H), 3.42 (q, *J* = 6.6 Hz, 2H), 2.83 (t, *J* = 7.0 Hz, 2H); ^13^C NMR (101 MHz, DMSO-*d*_6_): *δ* 161.1, 156.2, 155.1, 154.7, 149.9, 148.1, 148.1, 143.7, 131.8, 131.7, 129.4, 128.8, 128.7, 127.1, 125.9, 125.0, 124.8, 124.8, 115.7, 115.5, 109.9, 29.4; ^19^F NMR (376 MHz, Chloroform-*d*): *δ* −118.4; HRMS (ESI): [M + H]^+^ calcd for C_22_H_19_FN_5_O 388.1574, found 388.1573; RP-HPLC purity ≥ 99.6%, *t*_R_ = 18.4 min (method B).

1-(4-Fluorophenethyl)-3-(4-(quinazolin-7-yl)pyridin-2-yl)urea (**4n**)

This compound was prepared from 4-(quinazolin-7-yl)pyridin-2-amine (**3**) and 1-fluoro-4-(2-isocyanatoethyl)benzene according to the general procedure for the conversion of amine to urea (method A) as an off-white solid (42.0 mg, 48%). Mp: 214–217 °C. ^1^H NMR (400 MHz, DMSO-*d*_6_): *δ* 9.67 (s, 1H), 9.35 (s, 2H), 8.31 (d, *J* = 8.5 Hz, 1H), 8.25 (d, *J* = 5.5 Hz, 2H), 8.13 (s, 1H), 8.06–8.03 (m, 1H), 7.82 (s, 1H), 7.43 (dd, *J* = 5.4, 1.7 Hz, 1H), 7.28 (dd, *J* = 8.4, 5.7 Hz, 2H), 7.12 (t, *J* = 8.9 Hz, 2H), 3.43–3.38 (m, 2H), 2.78 (t, *J* = 7.0 Hz, 2H); ^13^C NMR (101 MHz, DMSO-*d*_6_): *δ* 162.5, 161.1, 160.1, 156.2, 155.1, 154.7, 149.9, 148.1, 143.7, 136.0, 131.0, 129.4, 127.1, 125.9, 125.0, 115.6, 115.5, 109.9, 41.1, 35.2; ^19^F NMR (376 MHz, Chloroform-*d*): *δ* −116.9; HRMS (ESI): [M + H]^+^ calcd for C_22_H_19_FN_5_O 388.1574, found 388.1573; RP-HPLC purity ≥ 99.7%, *t*_R_ = 18.8 min (method B).

General Procedure for the Conversion of Amine to Urea (Method B).

(i) To a solution of amine (0.17 mmol) and appropriate isocyanate (0.31 mmol) in THF (10 mL) at room temperature, DIPEA (0.52 mmol) was added. The resulting reaction mixture was heated at 70 °C for 12 h. After cooling to room temperature, the excess of solvent was evaporated to afford crude residue. The crude residue was taken to next step without purification. (ii) 4 N HCl (5 mL) in dioxane was added to a stirred solution of crude residue in MeOH (2 mL), and the mixture was stirred at room temperature for 2 h. The reaction mixture was basified using an aqueous NaHCO_3_ solution (30 mL) and extracted using ethyl acetate (3 × 30 mL). The combined organic layers were dried over anhydrous sodium sulfate, filtered, and evaporated. The crude residue was purified using silica gel column chromatography (0–5% MeOH in DCM).

1-(4-(3-Methoxy-1H-indazol-5-yl)pyridin-2-yl)-3-phenylurea (**8a**)

This compound was prepared from *tert*-butyl 5-(2-aminopyridin-4-yl)-3-methoxy-1*H*-indazole-1-carboxylate (**7**) and phenyl isocyanate according to the general procedure for the conversion of amine to urea (method B) as an off-white solid (14 mg, 22.10%). Mp: 218–221 °C. ^1^H NMR (400 MHz, DMSO-*d*_6_): *δ* 12.15 (s, 1H), 9.45 (s, 1H), 8.30 (d, *J* = 5.4 Hz, 1H), 7.93 (s, 1H), 7.83 (s, 1H), 7.72–7.67 (m, 1H), 7.57–7.50 (m, 3H), 7.43 (d, *J* = 8.4 Hz, 1H), 7.39 (d, *J* = 3.9 Hz, 1H), 7.33–7.26 (m, 2H), 7.04–6.98 (m, 1H), 4.02 (s, 3H); ^13^C NMR (101 MHz, DMSO-*d*_6_): *δ* 154.0, 152.7, 150.1, 142.4, 147.9, 139.4, 129.3, 129.2, 129.1, 129.0, 126.4, 119.2, 118.6, 117.9, 116.0, 114.2, 111.4, 109.0, 109.0, 56.3; HRMS (ESI): [M + H]^+^ calcd for C_20_H_18_N_5_O_2_ 360.1460, found 360.1457; RP-HPLC purity ≥ 93.4%, *t*_R_ = 21.7 min (method B).

1-(3-Fluorophenyl)-3-(4-(3-methoxy-1H-indazol-5-yl)pyridin-2-yl)urea (**8b**)

This compound was prepared from *tert*-butyl 5-(2-aminopyridin-4-yl)-3-methoxy-1*H*-indazole-1-carboxylate (**7**) and 3-fluorophenyl isocyanate according to the general procedure for the conversion of amine to urea (method B) as an off-white solid (12.0 mg, 18%). Mp: 229–232 °C. ^1^H NMR (400 MHz, DMSO-*d*_6_): *δ* 12.18–12.11 (m, 1H), 10.81–10.74 (m, 1H), 9.59–9.54 (m, 1H), 8.29 (d, *J* = 5.4 Hz, 1H), 7.91 (s, 1H), 7.82 (s, 1H), 7.67 (s, 1H), 7.60–7.55 (m, 1H), 7.51–7.47 (m, 1H), 7.39 (d, *J* = 5.5 Hz, 1H), 7.31 (s, 1H), 7.22 (s, 1H), 6.84–6.79 (m, 1H), 4.01 (s, 3H); ^13^C NMR (101 MHz, DMSO-*d*_6_): *δ* 173.1, 153.8, 152.7, 150.2, 147.9, 147.6, 147.5, 142.4, 130.9, 130.9, 130.8, 128.8, 115.9, 115.0, 115.0, 109.9, 109.1, 56.3; ^19^F NMR (376 MHz, DMSO-*d*_6_): *δ* −112.1; HRMS (ESI): [M + H]^+^ calcd for C_20_H_17_FN_5_O_2_ 378.1366, found 378.1364; RP-HPLC purity ≥ 97.4%, *t*_R_ = 24.6 min (method B).

1-(4-Fluorophenyl)-3-(4-(3-methoxy-1H-indazol-5-yl)pyridin-2-yl)urea (**8c**)

This compound was prepared from *tert*-butyl 5-(2-aminopyridin-4-yl)-3-methoxy-1*H*-indazole-1-carboxylate (**7**) and 4-fluorophenyl isocyanate according to the general procedure for the conversion of amine to urea (method B) as an off-white solid (10.0 mg, 15%). Mp: 229–232 °C. ^1^H NMR (400 MHz, DMSO-*d*_6_): *δ* 10.82 (s, 1H), 9.61 (s, 1H), 8.32 (d, *J* = 5.5 Hz, 1H), 7.94 (s, 1H), 7.86 (s, 1H), 7.72 (d, *J* = 8.8 Hz, 1H), 7.61 (d, *J* = 11.8 Hz, 1H), 7.42 (dd, *J* = 5.4, 1.6 Hz, 1H), 7.35 (dd, *J* = 15.0, 8.1 Hz, 1H), 7.24 (d, *J* = 8.1 Hz, 1H), 6.85 (dd, *J* = 9.2, 7.0 Hz, 2H), 4.04 (s, 3H); ^13^C NMR (101 MHz, DMSO-*d*_6_): *δ* 153.8, 152.6, 150.4, 150.2, 147.9, 147.6, 142.4, 130.9, 130.8, 128.8, 115.9, 115.8, 115.0, 109.9, 109.8, 109.3, 109.1, 56.3; ^19^F NMR (376 MHz, DMSO-*d*_6_): *δ* −112.1; HRMS (ESI): [M + H]^+^ calcd for C_20_H_17_FN_5_O_2_ 378.1366, found 378.1362; RP-HPLC purity ≥ 97.4%, *t*_R_ = 24.6 min (method B).

1-(4-(3-Methoxy-1H-indazol-5-yl)pyridin-2-yl)-3-(3-methoxyphenyl)urea (**8d**)

This compound was prepared from *tert*-butyl 5-(2-aminopyridin-4-yl)-3-methoxy-1*H*-indazole-1-carboxylate (**7**) and 3-methoxyphenyl isocyanate according to the general procedure for the conversion of amine to urea (method B) as a pale-yellow solid (15.0 mg, 21%). Mp: 230–233 °C. ^1^H NMR (400 MHz, DMSO-*d*_6_): *δ* 12.32 (s, 1H), 8.43 (d, *J* = 7.5 Hz, 1H), 8.26 (s, 1H), 7.96 (dd, *J* = 9.0, 1.7 Hz, 1H), 7.53 (d, *J* = 8.2 Hz, 2H), 7.50 (s, 1H), 7.41 (t, *J* = 8.0 Hz, 1H), 7.05–7.00 (m, 1H), 7.00–6.97 (m, 1H), 6.95 (d, *J* = 7.9 Hz, 1H), 4.06 (s, 3H), 3.78 (s, 3H); ^13^C NMR (101 MHz, DMSO-*d*_6_): *δ* 160.1, 157.7, 154.7, 154.7, 153.1, 148.7, 142.9, 137.4, 130.0, 129.6, 126.6, 126.0, 121.0, 120.0, 116.9, 114.3, 112.2, 112.1, 111.7, 56.4, 55.7; HRMS (ESI): [M + H]^+^ calcd for C_21_H_20_N_5_O_3_ 390.1566, found 390.1565; RP-HPLC purity ≥ 97.5%, *t*_R_ = 21.5 min (method B).

1-(4-(3-Methoxy-1H-indazol-5-yl)pyridin-2-yl)-3-(4-methoxyphenyl)urea (**8e**)

This compound was prepared from *tert*-butyl 5-(2-aminopyridin-4-yl)-3-methoxy-1*H*-indazole-1-carboxylate (**7**) and 4-methoxyphenyl isocyanate according to the general procedure for the conversion of amine to urea (method B) as an off-white solid (17.0 mg, 24%). Mp: 240–242 °C. ^1^H NMR (400 MHz, DMSO-*d*_6_): *δ* 12.16 (s, 1H), 10.42 (s, 1H), 9.40 (s, 1H), 8.30 (d, *J* = 5.5 Hz, 1H), 7.94 (s, 1H), 7.81 (s, 1H), 7.71 (dd, *J* = 8.8, 1.7 Hz, 1H), 7.52 (d, *J* = 8.7 Hz, 1H), 7.45 (d, *J* = 9.0 Hz, 2H), 7.38 (dd, *J* = 5.5, 1.6 Hz, 1H), 6.90 (d, *J* = 9.0 Hz, 2H), 4.04 (s, 3H), 3.74 (s, 3H); ^13^C NMR (101 MHz, DMSO-*d*_6_): *δ* 157.3, 155.3, 154.1, 152.8, 152.8, 150.1, 147.8, 142.4, 132.4, 128.8, 126.4, 121.0, 117.9, 115.5, 114.5, 114.3, 112.0, 111.4, 109.9, 109.0, 56.3, 55.6; HRMS (ESI): [M + H]^+^ calcd for C_21_H_20_N_5_O_3_ 390.1566, found 390.1556; RP-HPLC purity ≥ 98.5%, *t*_R_ = 20.9 min (method B).

1-(3-Chloro-4-fluorophenyl)-3-(4-(3-methoxy-1H-indazol-5-yl)pyridin-2-yl)urea (**8f**)

This compound was prepared from *tert*-butyl 5-(2-aminopyridin-4-yl)-3-methoxy-1*H*-indazole-1-carboxylate (**7**) and 2-chloro-1-fluoro-4-isocyanatobenzene according to the general procedure for the conversion of amine to urea (method B) as an off-white solid (17.0 mg, 23%). Mp: 300–305 °C. ^1^H NMR (400 MHz, DMSO-*d*_6_): *δ* 12.05 (s, 1H), 9.86 (s, 1H), 7.92 (d, *J* = 5.4 Hz, 1H), 7.81 (s, 1H), 7.67 (d, *J* = 5.0 Hz, 1H), 7.61 (d, *J* = 8.8 Hz, 1H), 7.43 (d, *J* = 8.7 Hz, 1H), 7.34 (d, *J* = 8.6 Hz, 2H), 6.81 (d, *J* = 5.2 Hz, 1H), 6.73 (s, 1H), 5.89 (s, 1H), 4.01 (s, 3H); ^13^C NMR (101 MHz, DMSO-*d*_6_): *δ* 160.8, 157.2, 154.3, 148.8, 142.2, 136.9, 129.9, 126.3, 119.7, 119.5, 118.7, 117.5 117.3, 117.2, 111.9, 111.1, 110.5, 109.9, 105.2, 56.3; ^19^F NMR (376 MHz, DMSO-*d*_6_): *δ* −124.5; HRMS (ESI): [M + H]^+^ calcd for C_20_H_16_ClFN_5_O_2_ 412.0977, found 412.0973; RP-HPLC purity ≥ 95.4%, *t*_R_ = 31.2 min (method B).

1-Cyclopentyl-3-(4-(3-methoxy-1H-indazol-5-yl)pyridin-2-yl)urea (**8g**)

This compound was prepared from *tert*-butyl 5-(2-aminopyridin-4-yl)-3-methoxy-1*H*-indazole-1-carboxylate (**7**) and cyclopentyl isocyanate according to the general procedure for the conversion of amine to urea (method B) as an off-white solid (25.0 mg, 40%). Mp: 222–225 °C. ^1^H NMR (400 MHz, DMSO-*d*_6_): δ 12.13 (s, 1H), 9.04 (s, 1H), 8.18 (d, *J* = 5.4 Hz, 1H), 8.12 (s, 1H), 7.88 (s, 1H), 7.72 (s, 1H), 7.66 (d, *J* = 8.6 Hz, 1H), 7.48 (d, *J* = 8.7 Hz, 1H), 7.27 (d, *J* = 4.7 Hz, 1H), 4.02 (s, 3H), 3.98 (d, *J* = 6.5 Hz, 1H), 1.87 (dd, *J* = 11.9, 5.5 Hz, 2H), 1.65 (s, 2H), 1.55 (s, 2H), 1.45–1.37 (m, 2H); ^13^C NMR (101 MHz, DMSO-*d*_6_): δ 157.3, 154.7, 154.6, 149.8, 147.8, 142.3, 129.0, 126.4, 117.8, 115.0, 112.0, 111.4, 108.6, 56.3, 51.2, 33.3, 23.6; HRMS (ESI): [M + H]^+^ calcd for C_19_H_22_N_5_O_2_ 352.1773, found 352.1773; RP-HPLC purity ≥ 98.5%, *t*_R_ = 18.1 min (method B).

1-Benzyl-3-(4-(3-methoxy-1H-indazol-5-yl)pyridin-2-yl)urea (**8h**)

This compound was prepared from *tert*-butyl 5-(2-aminopyridin-4-yl)-3-methoxy-1*H*-indazole-1-carboxylate (**7**) and (isocyanatomethyl)benzene according to the general procedure for the conversion of amine to urea (method B) as a pale-yellow solid (22.0 mg, 33%). Mp: 230–233 °C. ^1^H NMR (400 MHz, DMSO-*d*_6_): *δ* 12.12 (s, 1H), 9.29 (s, 1H), 8.65 (s, 1H), 8.19 (d, *J* = 5.4 Hz, 1H), 7.89 (s, 1H), 7.70 (s, 1H), 7.66 (dd, *J* = 8.8, 1.6 Hz, 1H), 7.48 (d, *J* = 8.7 Hz, 1H), 7.34–7.28 (m, 5H), 7.27–7.21 (m, 1H), 4.41 (d, *J* = 5.9 Hz, 2H), 4.02 (s, 3H); ^13^C NMR (101 MHz, DMSO-*d*_6_): *δ* 157.3, 155.4, 154.5, 149.9, 147.7, 142.4, 140.4, 128.9, 128.8, 127.4, 127.2, 126.4, 117.8, 115.1, 112.0, 111.4, 108.8, 56.3, 43.0; HRMS (ESI): [M + H]^+^ calcd for C_21_H_20_N_5_O_2_ 374.1617, found 374.1617; RP-HPLC purity ≥ 96.8%, *t*_R_ = 19.0 min (method B).

1-(4-(3-Methoxy-1H-indazol-5-yl)pyridin-2-yl)-3-phenethylurea (**8i**)

This compound was prepared from *tert*-butyl 5-(2-aminopyridin-4-yl)-3-methoxy-1*H*-indazole-1-carboxylate (**7**) and phenethyl isocyanate according to the general procedure for the conversion of amine to urea (method B) as a pale-yellow solid (18.0 mg, 26%). Mp: 200–203 °C. ^1^H NMR (400 MHz, DMSO-*d*_6_): *δ* 12.13 (s, 1H), 9.22 (s, 1H), 8.36 (s, 1H), 8.10 (d, *J* = 5.2 Hz, 1H), 7.88 (s, 1H), 7.67–7.59 (m, 2H), 7.48 (d, *J* = 8.9 Hz, 1H), 7.33–7.19 (m, 5H), 4.02 (s, 3H), 3.43 (d, *J* = 6.3 Hz, 2H), 2.79 (t, *J* = 6.9 Hz, 2H); ^13^C NMR (101 MHz, DMSO-*d*_6_): *δ* 157.3, 155.2, 154.5, 149.8, 147.5, 142.4, 139.9, 129.2, 128.9, 128.8, 126.5, 126.4, 117.8, 115.0, 112.0, 111.4, 108.6, 56.3, 41.1, 36.1; HRMS (ESI): [M + H]^+^ calcd for C_22_H_22_N_5_O_2_ 388.1773, found 388.1773; RP-HPLC purity ≥ 99.5%, *t*_R_ = 20.6 min (method B).

1-(2-Fluorophenethyl)-3-(4-(3-methoxy-1H-indazol-5-yl)pyridin-2-yl)urea (**8j**)

This compound was prepared from *tert*-butyl 5-(2-aminopyridin-4-yl)-3-methoxy-1*H*-indazole-1-carboxylate (**7**) and 1-fluoro-2-(2-isocyanatoethyl)benzene according to the general procedure for the conversion of amine to urea (method B) as a pale-yellow solid (16.0 mg, 22%). Mp: 208–211 °C. ^1^H NMR (400 MHz, DMSO-*d*_6_): *δ* 12.13 (s, 1H), 9.22 (s, 1H), 8.35 (s, 1H), 8.11 (d, *J* = 5.5 Hz, 1H), 7.88 (s, 1H), 7.67–7.60 (m, 2H), 7.48 (d, *J* = 8.8 Hz, 1H), 7.29 (dd, *J* = 10.5, 6.1 Hz, 3H), 7.13 (t, *J* = 8.9 Hz, 2H), 4.02 (s, 3H), 3.41 (dd, *J* = 13.1, 6.9 Hz, 2H), 2.78 (t, *J* = 7.0 Hz, 2H); ^13^C NMR (101 MHz, DMSO-*d*_6_): *δ* 155.2, 154.5, 149.8, 147.5, 142.4, 131.0, 130.9, 128.9, 126.4, 117.8, 115.5, 115.3, 115.0, 112.0, 111.4, 109.9, 108.6, 56.3, 41.1, 35.2; ^19^F NMR (376 MHz, DMSO-*d*_6_): *δ* −117.1; RP-HPLC purity ≥ 96.9%, *t*_R_ = 21.1 min (method B).

1-(4-Fluorophenethyl)-3-(4-(3-methoxy-1H-indazol-5-yl)pyridin-2-yl)urea (**8k**)

This compound was prepared from *tert*-butyl 5-(2-aminopyridin-4-yl)-3-methoxy-1*H*-indazole-1-carboxylate (**7**) and 1-fluoro-4-(2-isocyanatoethyl)benzene according to the general procedure for the conversion of amine to urea (method B) as a pale yellow solid (20.0 mg, 28%). Mp: 209–212 °C. ^1^H NMR (400 MHz, DMSO-*d*_6_): *δ* 12.13 (s, 1H), 9.22 (s, 1H), 8.34 (s, 1H), 8.11 (d, *J* = 5.3 Hz, 1H), 7.88 (s, 1H), 7.65 (d, *J* = 8.8 Hz, 1H), 7.61 (s, 1H), 7.48 (d, *J* = 8.9 Hz, 1H), 7.29 (dd, *J* = 10.5, 6.3 Hz, 3H), 7.13 (t, *J* = 8.9 Hz, 2H), 4.02 (s, 3H), 3.45–3.38 (m, 2H), 2.78 (t, *J* = 7.1 Hz, 2H); ^13^C NMR (101 MHz, DMSO-*d*_6_): *δ* 155.2, 150.7, 149.8, 147.5, 142.4, 131.0, 130.9, 128.9, 126.4, 117.8, 115.5, 115.3, 115.0, 112.0, 111.4, 109.9, 108.6, 56.3, 41.1, 35.2; ^19^F NMR (376 MHz, DMSO-*d*_6_): *δ* −117.1; HRMS (ESI): [M + H]^+^ calcd for C_22_H_21_FN_5_O_2_ 406.1679, found 406.1678; RP-HPLC purity ≥ 97.5%, *t*_R_ = 21.1 min (method B).

### 4.2. Biology

#### 4.2.1. Cell Culture

Breast cancer cell line MCF7 and colorectal carcinoma cell line HCT116 were obtained from the National Cancer Institute (MTA Number: 2702-09). Cells were cultured in complete RPMI 1640 medium (Hyclone, Logan, UT, USA) containing 10% FBS (Hyclone, Logan, UT, USA) in an atmosphere of 5% CO 2/100% humidity at 37 °C. Each investigated compound was diluted using DMSO to make a stock solution. It was treated by mixing with cell culture medium (RPMI1640 containing 10% FBS (Hyclone, Logan, UT, USA)) so that the final concentration of the drug was 0.001, 0.01, 0.1, 1, or 10 uM. There was no compound precipitated upon treatment in the culture medium.

#### 4.2.2. SRB Assay of Antiproliferative Activity

The SRB assay was performed following the methodology of previously published studies [51]. In brief, the cells (100 μL medium containing 5000 HCT116 or 10,000 MCF7 cells/well) were incubated in 96-well microtiter plates. After 24 h, the drug was added (100 μL) to each well and the cultures were incubated for 48 h at 37 °C. The cells were fixed in 50% TCA (50 μL per well) for 1 h at 4 °C. The liquid was removed from the plate, which was then rinsed five times with water and allowed to dry at room temperature (RT). Washed cells were stained for 10 min at RT with 0.4% SRB (100 μL per well). After staining, the plate was washed 3 times with 1% glacial acetic acid and dried at RT. The SRB stain was then solubilized in 10 mM Tris and absorbances were read at 515 nm. The effect of the drugs was expressed in terms of the GI_50_ (the concentration resulting in 50% maximal inhibition of cell proliferation). Each experiment was repeated at least three times, and the GI50 value calculated based on the results was expressed as mean ± standard deviation.

#### 4.2.3. SRB Assay of Antiproliferative Activity on Normal Cells

The antiproliferative activity of compounds **8a**, **8h**, and **8i** was evaluated in a sulforhodamine B (SRB) assay using normal cell line HEK293 cells. Cells were treated for 48 h under the same conditions used for the assessment of the antiproliferative activity on the cancer cells.

### 4.3. Molecular Docking

The crystal structure of VEGFR2 (juxtamembrane and kinase domains) in complex with Tivozanib (Supporting Information, Figure S69) was downloaded from the protein data bank (PDB ID: 4ASE; resolution: 1.83 Å) [52]. It is interesting to note that Tivozanib possessed a urea linker as well as a methoxy group on its “tail moiety”. During the protein preparation phase, several actions were taken. Firstly, water molecules and crystallized inhibitor (Tivozanib) were removed, while any missing residues and hydrogen atoms were added. Subsequently, the prepared protein structure was protonated to attain a state suitable for physiological pH conditions. Finally, energy minimization was performed using the OPLS-2005 force field and a conjugate gradient method. This optimization process aimed to achieve a unique low-energy minimum for the protein structure. The Ligprep module of Maestro Schrodinger 2021.2 was implemented to prepare the proteins, involving the removal of water molecules and co-crystals as well as the inclusion of any missing residues or hydrogen atoms. The Glide extra precision module of Maestro Schrodinger was used for docking the compounds with each protein structure, producing 32 poses for each ligand. To establish the center of the Glide grid, the position of the co-crystallized ligand was utilized. Default settings were employed for both the grid generation and the docking processes. Using BIOVIA Discovery Studio Visualizer 2021 software, the pose with the lowest energy score was chosen for visualization [53,54].

### 4.4. MD Calculations

DESMOND software (version 2021.2) was utilized to perform molecular dynamics (MD) simulations with specific parameters. The simulation system was constructed by creating an orthorhombic box, which was solvated using single-point charge (SPC) water molecules. The temperature of the system was maintained at 300 K, and the pressure was set to 1 bar throughout the MD run. The simulation duration was set to 100 ns, and a relaxation time of 1 ps was applied for the selected positions. To minimize the energy of the solvated system, OPLS 2005 force parameters were employed. Electrostatic interactions were handled using the particle mesh Ewald (PME) method, while periodic boundary conditions (PBC) and a nonbond interaction cutoff distance of 9.0 Å (Angstrom) were implemented [53].

For each MD simulation, a six-step relaxation protocol was followed. This involved 2000 steps of LBFGS minimization, with the solute being restricted, and a loose convergence criteria of 50 kcal mol^−1^ Å^−1^. Two preliminary simulations were conducted: a 12 ns simulation to equilibrate temperature (T = 10 K) with a thermostat relaxation constant of 0.1 ps, and a 12 ns simulation to equilibrate pressure (P = 1 atm) with a barostat relaxation constant of 50 ps. In both preliminary simulations, the nonhydrogen solute atoms were restrained. Subsequently, a 24 ps NPT ensemble simulation was performed to complete the preparation processes, with a temperature of 300 K, a thermostat relaxation constant of 0.1 ps, and a pressure of 1 atm. After the relaxation phase, the systems underwent a 5 ns MD simulation in the NPT ensemble. This simulation utilized a Nose–Hoover thermostat and Martyna–Tobias–Klein barostat, maintaining a temperature of 300 K with a thermostat relaxation time of 1.0 ps, a pressure of 1 atm, and a barostat relaxation time of 2.0 ps. The resulting MD simulation data were analyzed using QtGrace (version 2009) and Microsoft Excel [53].

### 4.5. Molecular Mechanics–Generalized Born Surface Area (MM–GBSA) Calculations

The MM–GBSA method, which combines molecular mechanics and the generalized Born surface area method, was used to calculate the binding free energy of ligand–protein complexes [55]. The thermal mmgbsa.py script was used to perform the calculations, which employed the 0–100 ns MD simulation trajectories, the VSGB solvation model, the OPLS3e force field, and a sampling rate of 10 steps per ns [56]. The binding free energy was calculated using the law of additivity, which took into account a variety of energy components such as hydrogen bonding, van der Waals interactions, columbic interactions, lipophilic interactions, covalent interactions, solvation, stacking, and self-contact of the ligand and protein [57].

### 4.6. In Silico Pharmacokinetic Study

The in silico pharmacokinetics of the most active compounds were predicted using the SwissADME server [58].

## Figures and Tables

**Table 1:** GI_50_ of the synthesized compounds over MCF7 and HCT116 cancer cell lines.

Compd.	GI_50_ (μM) *	
	MCF7	HCT116
**4a**	>10	>10
**4b**	>10	>10
**4c**	>10	>10
**4d**	>10	>10
**4e**	>10	>10
**4f**	>10	>10
**4g**	>10	>10
**4h**	>10	>10
**4i**	3.35 ± 0.101	>10
**4j**	>10	>10
**4k**	3.03 ± 0.061	>10
**4l**	9.00 ± 0.347	>10
**4m**	>10	>10
**4n**	>10	>10
**8a**	0.06 ± 0.014	1.19 ± 0.035
**8b**	>10	>10
**8c**	3.73 ± 0.075	1.22 ± 0.141
**8d**	1.89 ± 0.26	>10
**8e**	4.37 ± 0.359	>10
**8f**	0.89 ± 0.209	0.92 ± 0.114
**8g**	1.17 ± 0.059	1.25 ± 0.037
**8h**	0.23 ± 0.015	0.33 ± 0.042
**8i**	0.16 ± 0.013	1.08 ± 0.006
**8j**	0.75 ± 0.142	>10
**8k**	1.13 ± 0.003	1.38 ± 0.086
**Sorafenib**	11.2 [31]	-
**Irinotecan**	0.35 ± 0.069	0.62 ± 0.003

* All values are expressed as mean ± standard error (*n* = 3).

**Table 2:** MM–GBSA calculations for the bound complexes of compounds **8a**, **8h**, and **8i**.

Complex Name	MM-GBSA (kcal/mol)				
	Average ΔG_Bind_	Average ΔG_Coulomb_	Average ΔG_H_bond_	Average ΔG_Lipo_	Average ΔG_Solv_GB_
Compound **8a**–VEGFR2 complex	−55.64	−9.25	−0.88	−21.71	25.87
Compound **8h**–VEGFR2 complex	−48.78	−9.65	−0.84	−20.40	27.55
Compound **8i**–VEGFR2 complex	−59.55	−16.67	−1.17	−20.86	30.61

**Table 3:** Predicted pharmacokinetic properties of compounds **8a**, **8h**, and **8i**.

Compound	Lipinski Violations	Solubility in Water	BBB Permeant	GI Absorption
**8a**	0	Moderately soluble	No	High
**8h**	0	Moderately soluble	No	High
**8i**	0	Moderately soluble	No	High

## Data Availability

In vitro and MD raw data can be found in the raw data file.

## Supplementary Materials

The following are available online at https://www.mdpi.com/article/10.3390/molecules28134952/s1, Schemes and synthesis of the biologically tested compounds.

## Sample Availability

Samples of the compounds are available from the authors.
