# Supplementary material for: Integration of Hybridization Strategies in Pyridine–Urea Scaffolds for Novel Anticancer Agents: Design, Synthesis, and Mechanistic Insights
Source: Molecules. 2023 Jun 23;28(13):4952. doi: 10.3390/molecules28134952 (PMC10343686; doi:10.3390/molecules28134952)

Supporting Information

Table of Contents

^{1}H NMR and ^{13}C NMR spectra of compounds.	S1−S50
HPLC of compounds.	S51−S59
HR-ESIMS of compounds.	S60−S67
2D structures of Irinotecan and Tivozanib	S68
2D interaction of VEGFR2 complexed with Tivozanib	S69
RMSD plot of ligand-protein complexes	S70
Protein-ligand contact diagrams for compounds 8a, 8h and 8i	S71

Figure S1. ^{1}H NMR (400 MHz, DMSO-d_6) spectrum of compound **4a**

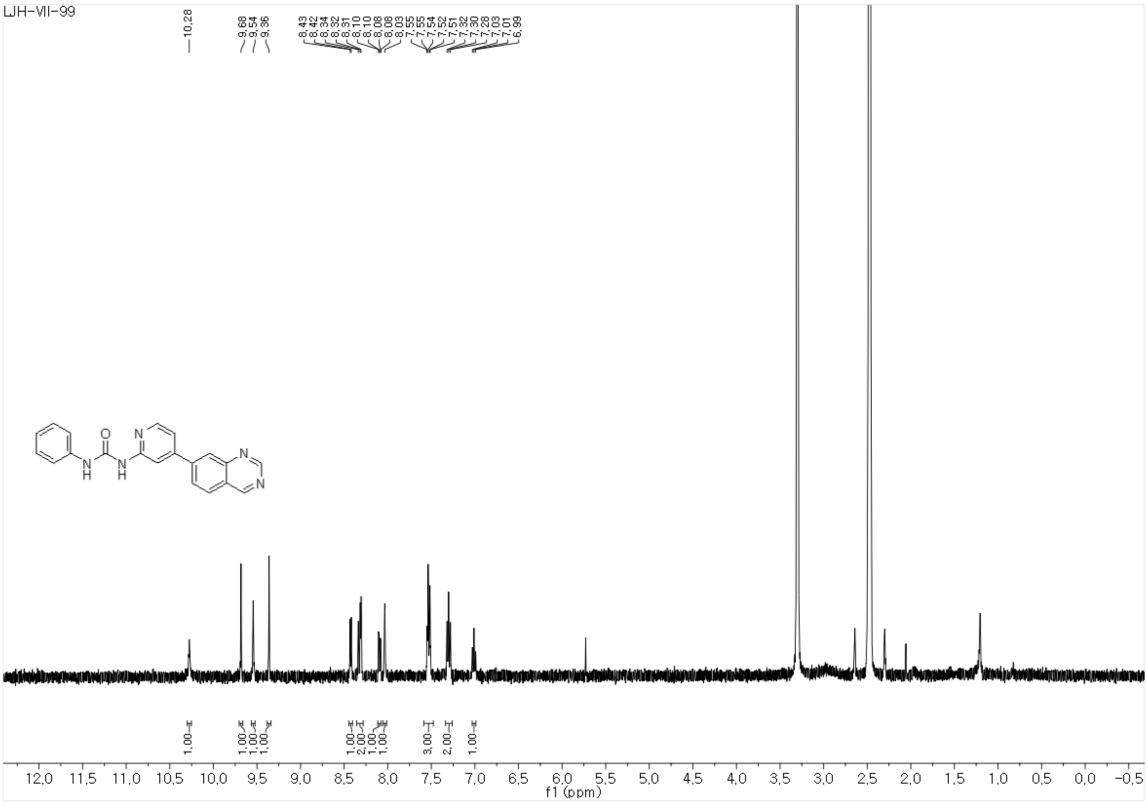

Figure S2. ^{13}C NMR (100 MHz, DMSO-d_6) spectrum of compound **4a**

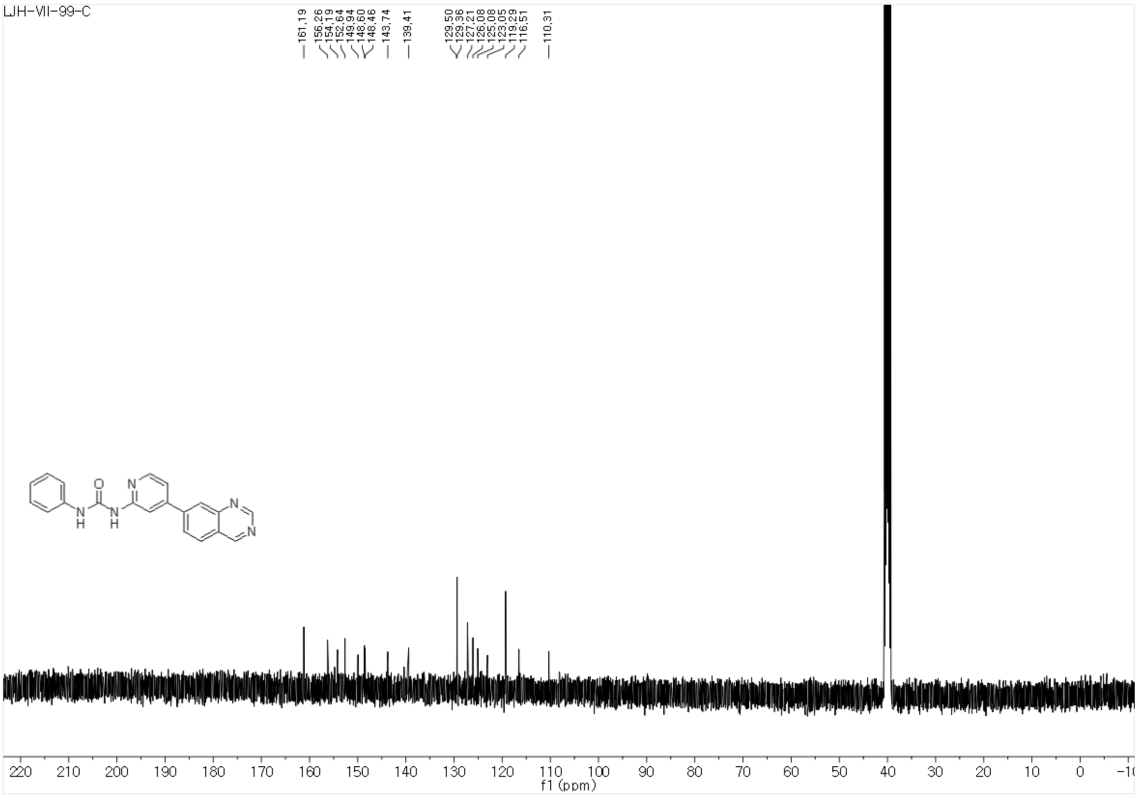

Figure S3. ^{1}H NMR (400 MHz, DMSO-d_6) spectrum of compound **4b**

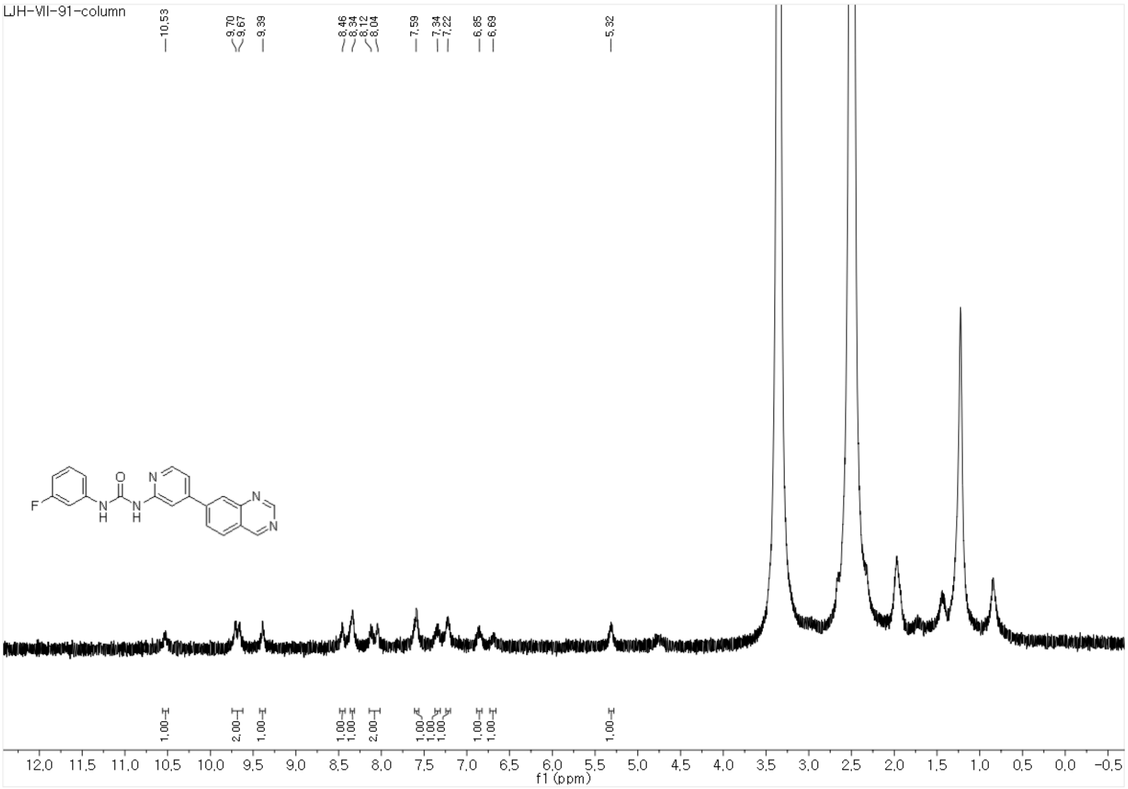

Figure S4. ^{13}C NMR (100 MHz, DMSO-d_6) spectrum of compound **4b**

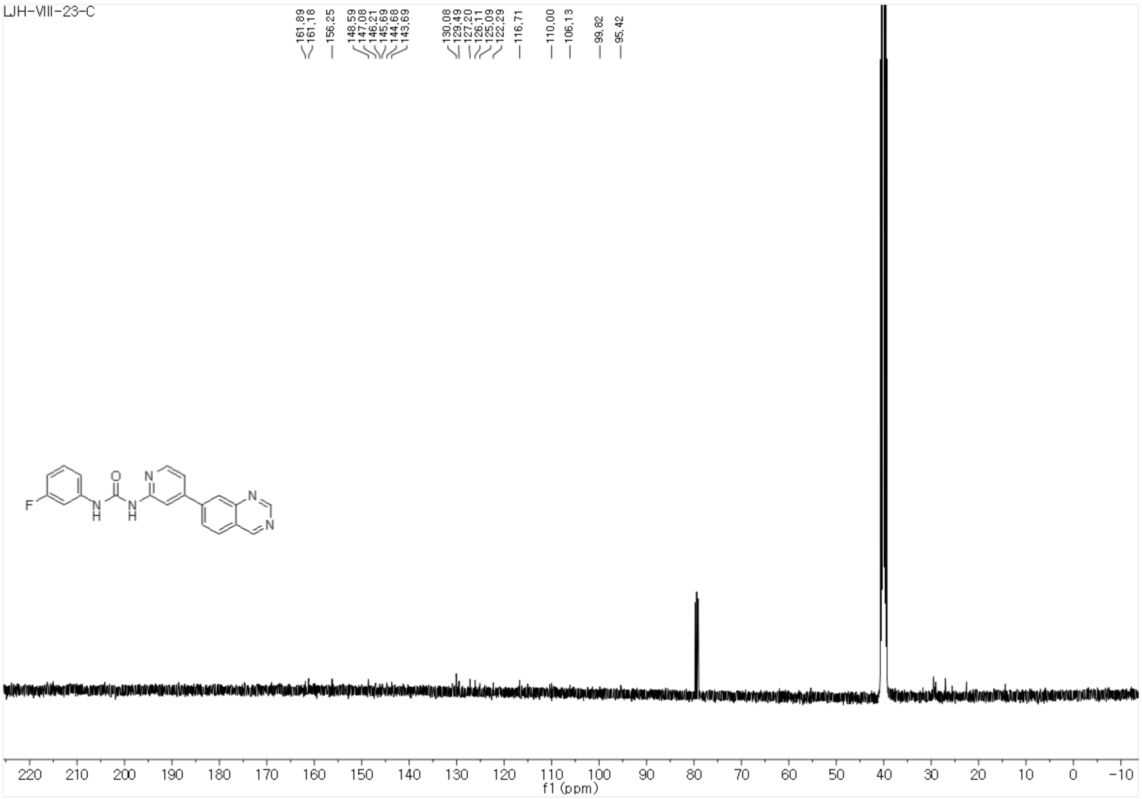

Figure S5. ^{1}H NMR (400 MHz, DMSO-d_6) spectrum of compound **4c**

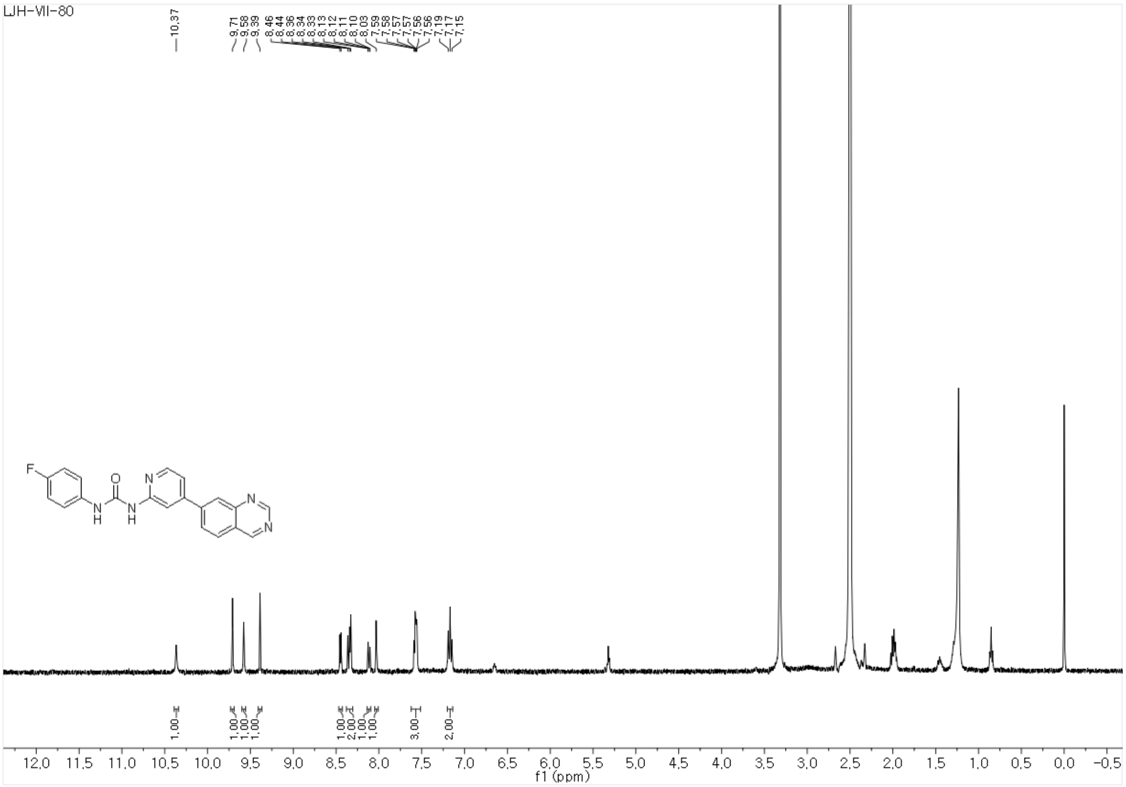

Figure S6. ^{13}C NMR (100 MHz, DMSO-d_6) spectrum of compound **4c**

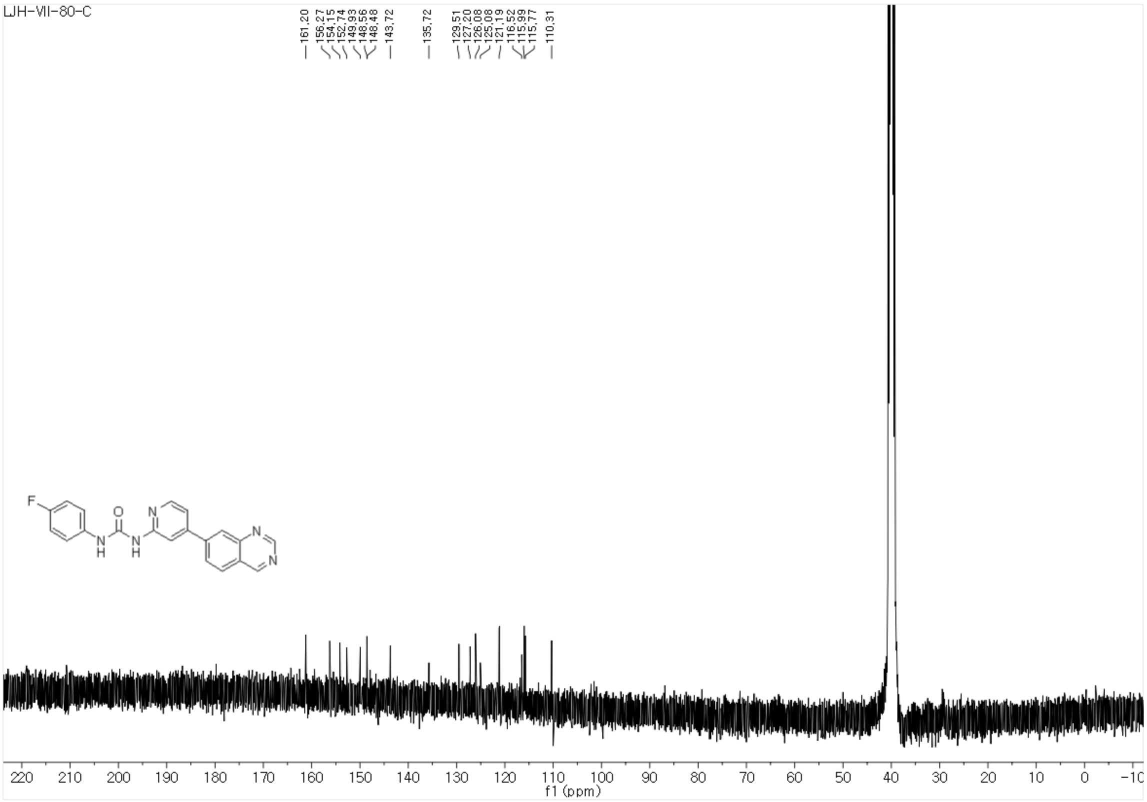

Figure S7. ^{1}H NMR (400 MHz, DMSO-d_6) spectrum of compound **4d**

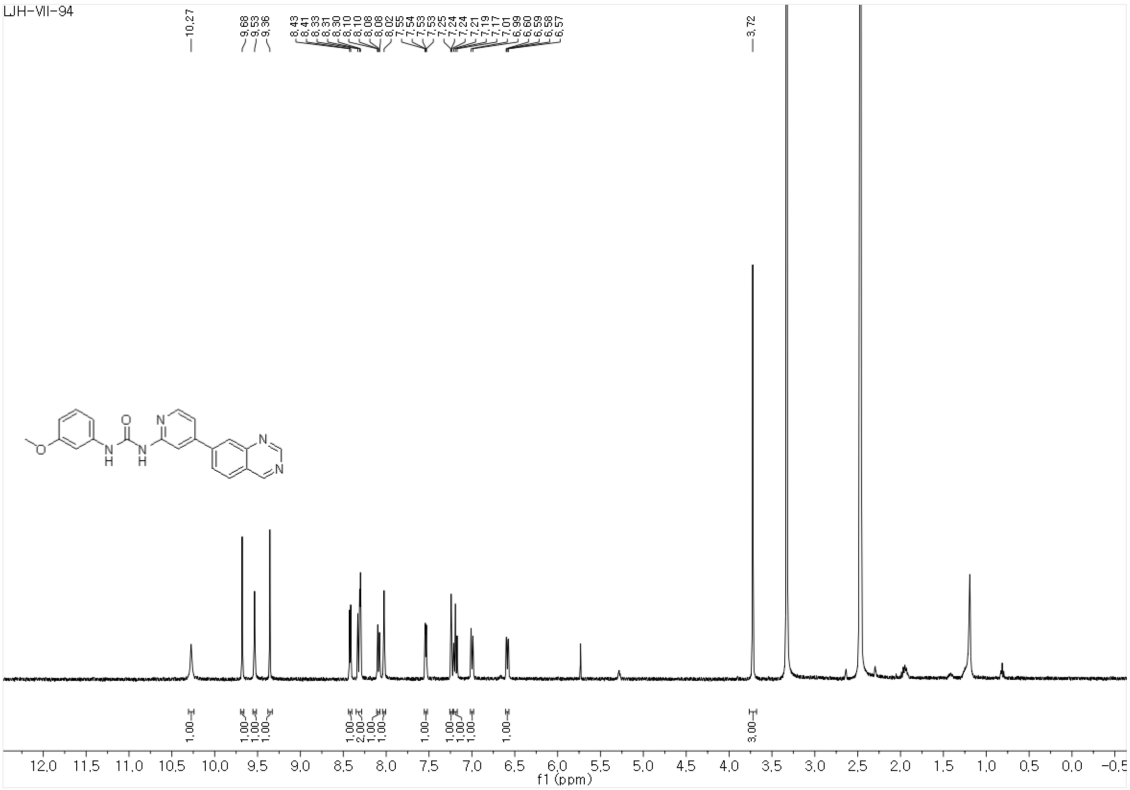

Figure S8. ^{13}C NMR (100 MHz, DMSO-d_6) spectrum of compound **4d**

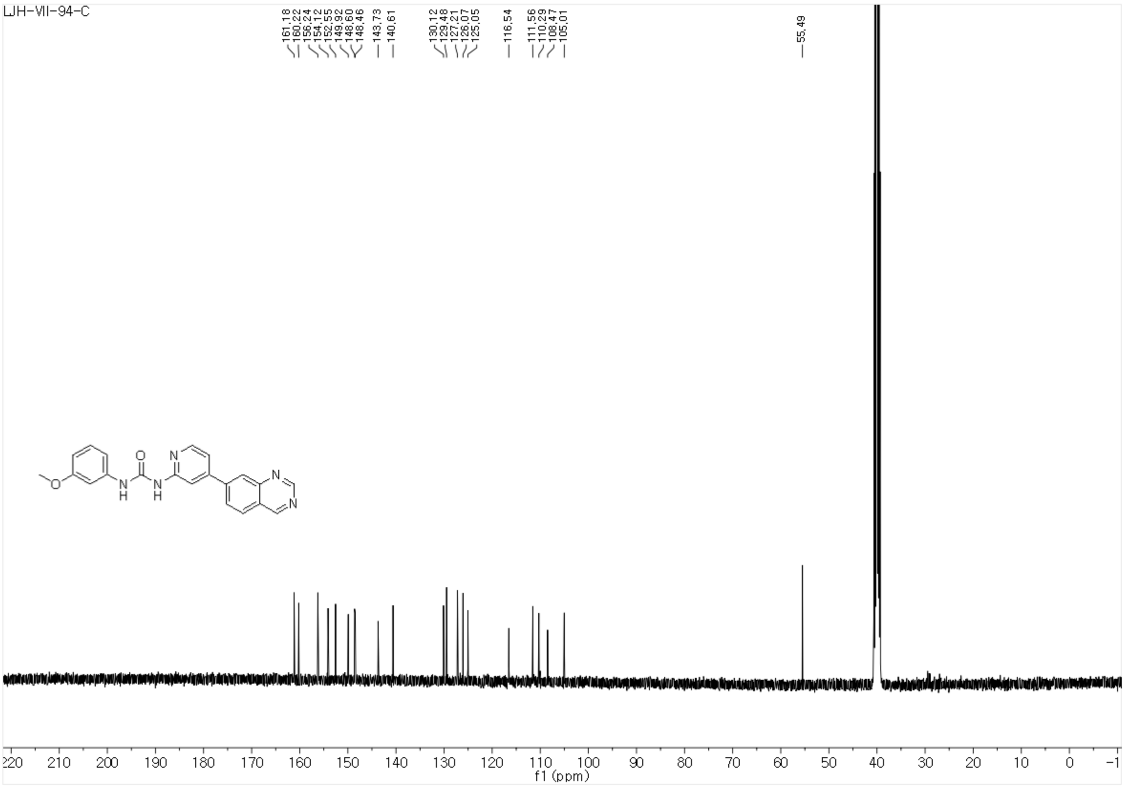

Figure S9. ^{1}H NMR (400 MHz, DMSO-d_6) spectrum of compound **4e**

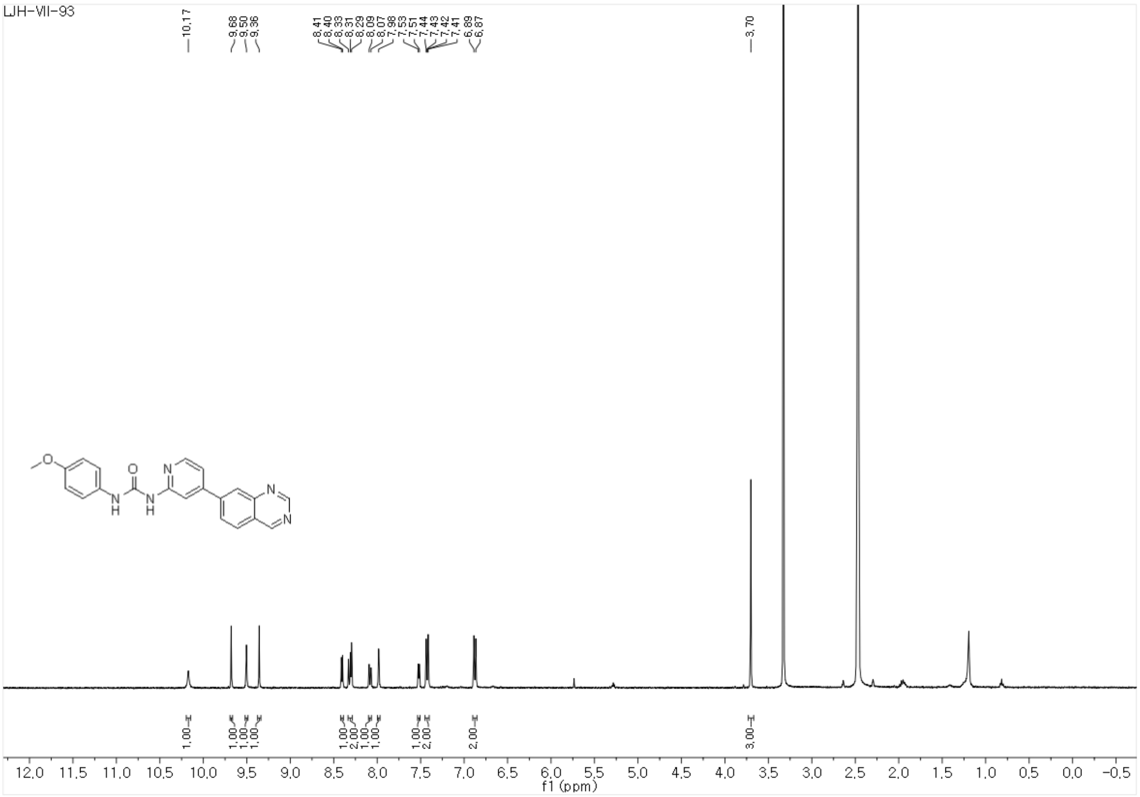

Figure S10. ^{13}C NMR (100 MHz, DMSO-d_6) spectrum of compound **4e**

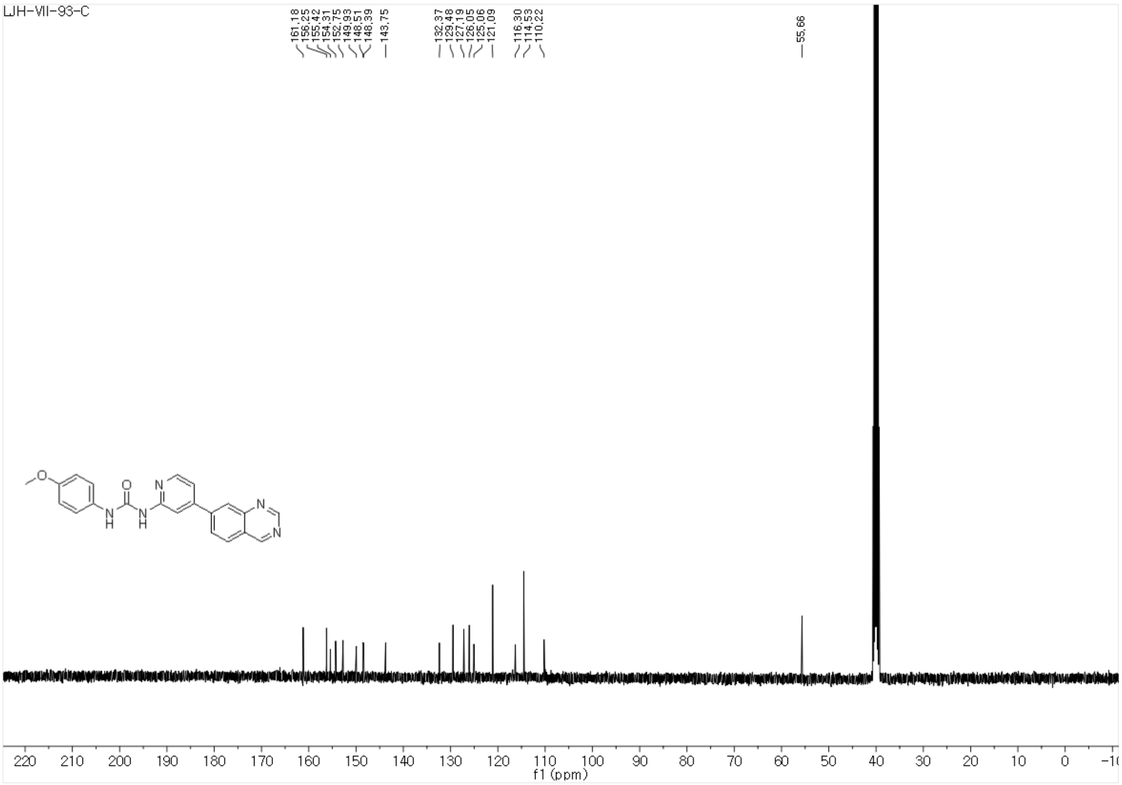

Figure S11. ^{1}H NMR (400 MHz, DMSO-d_6) spectrum of compound **4f**

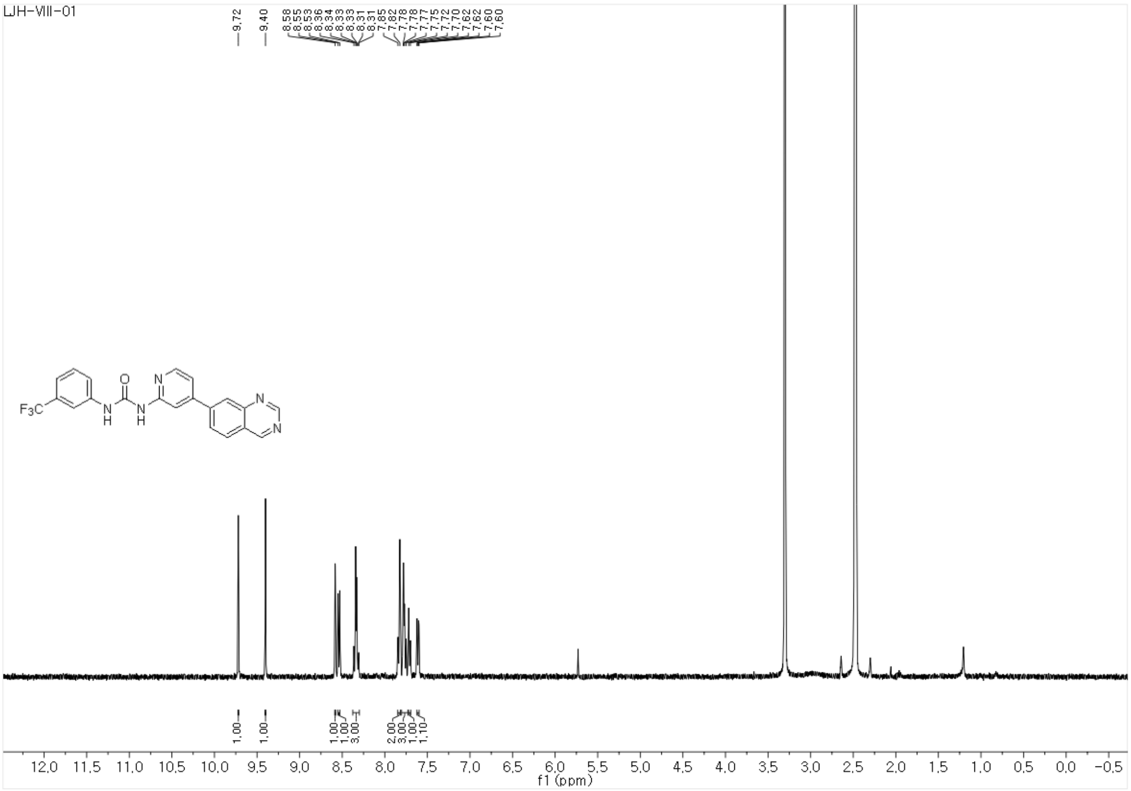

Figure S12. ^{13}C NMR (100 MHz, DMSO-d_6) spectrum of compound **4f**

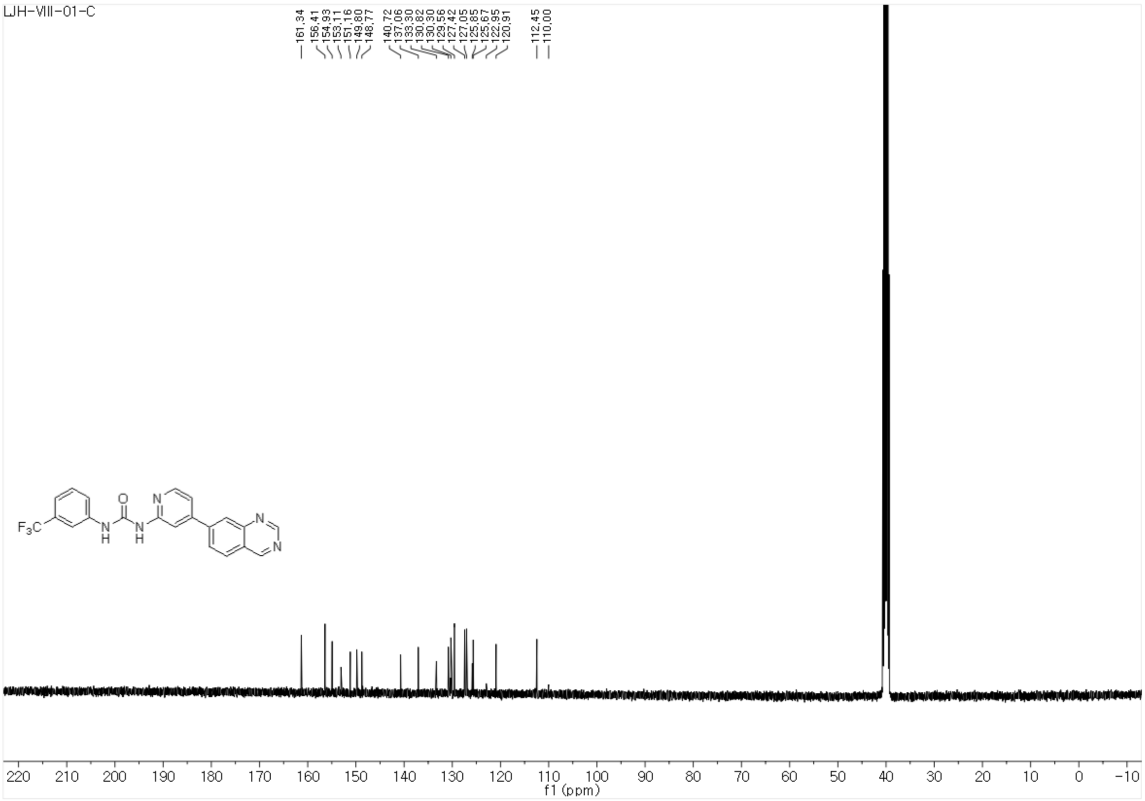

Figure S13. ^{1}H NMR (400 MHz, Chloroform-*d*) spectrum of compound **4g**

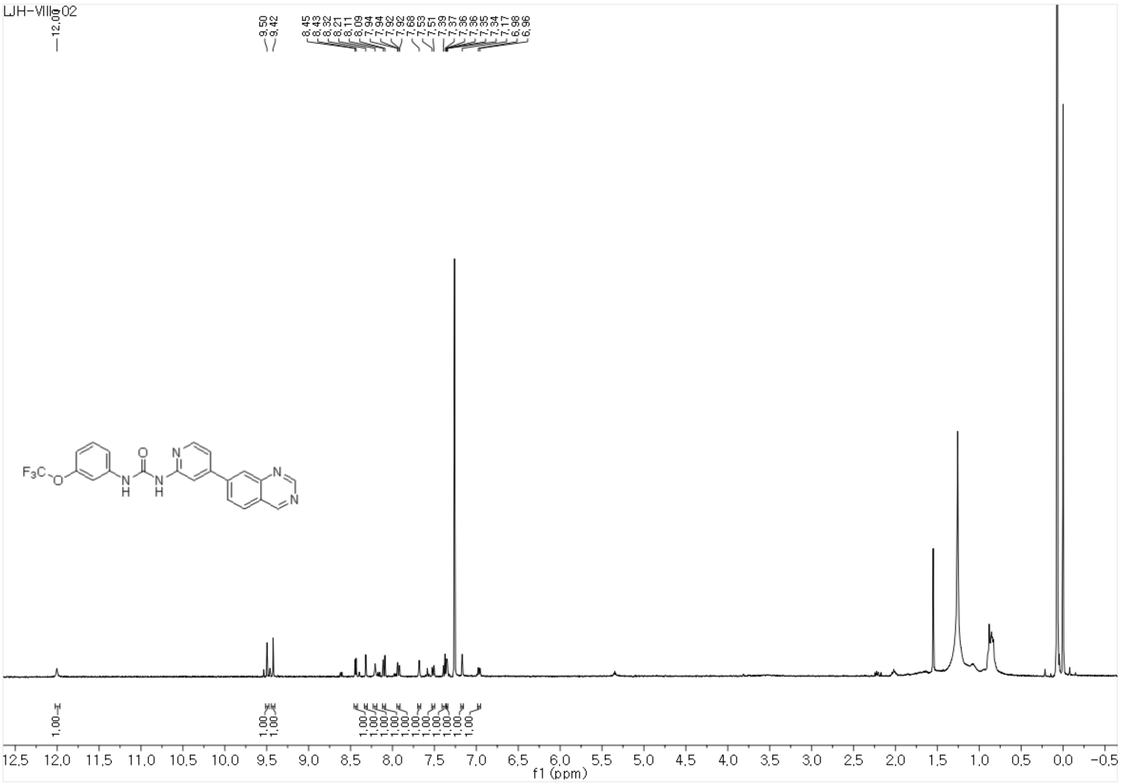

Figure S14. ^{13}C NMR (100 MHz, Chloroform-*d*) spectrum of compound **4g**

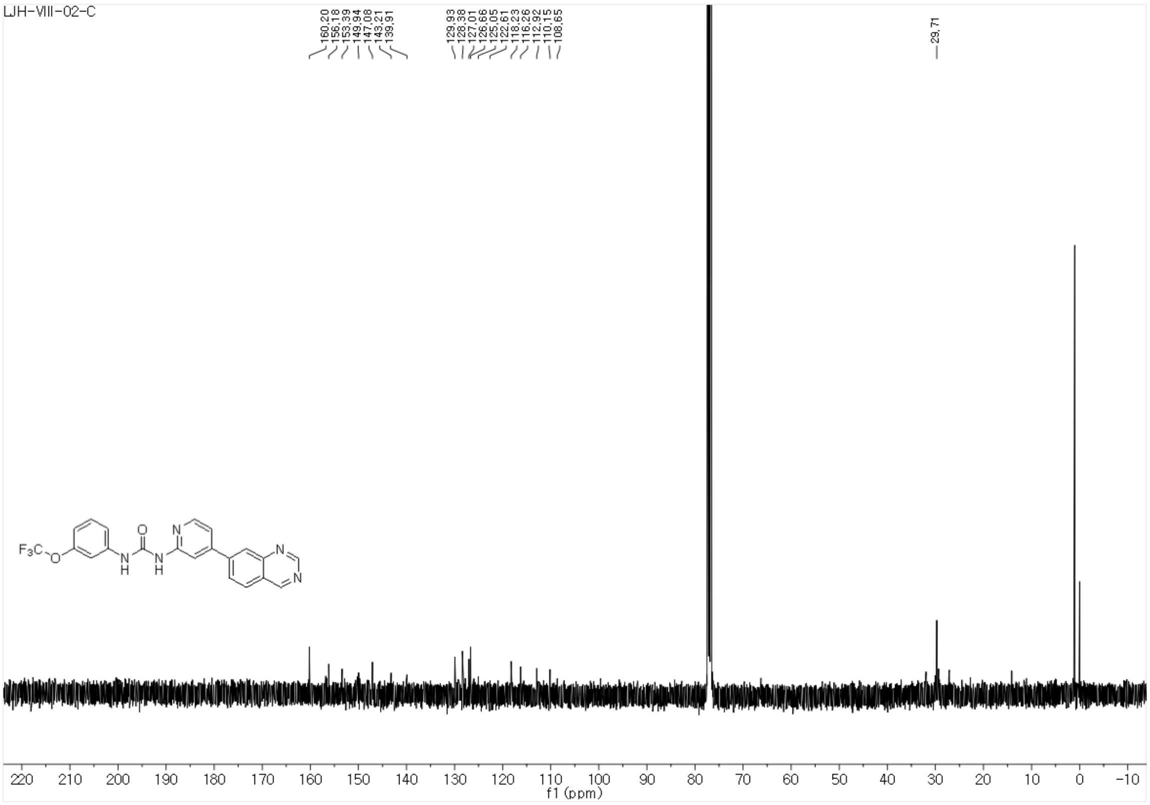

Figure S15. ^{1}H NMR (400 MHz, DMSO-d_6) spectrum of compound **4h**

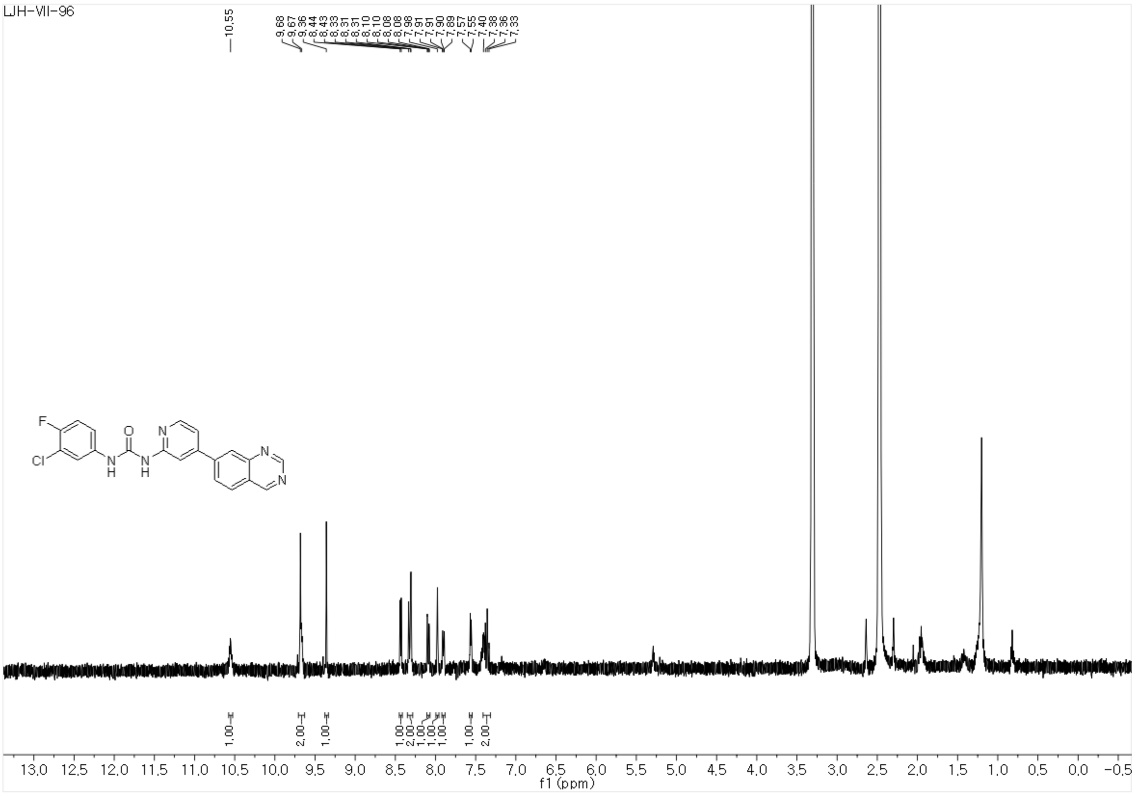

Figure S16. ^{13}C NMR (100 MHz, DMSO-d_6) spectrum of compound **4h**

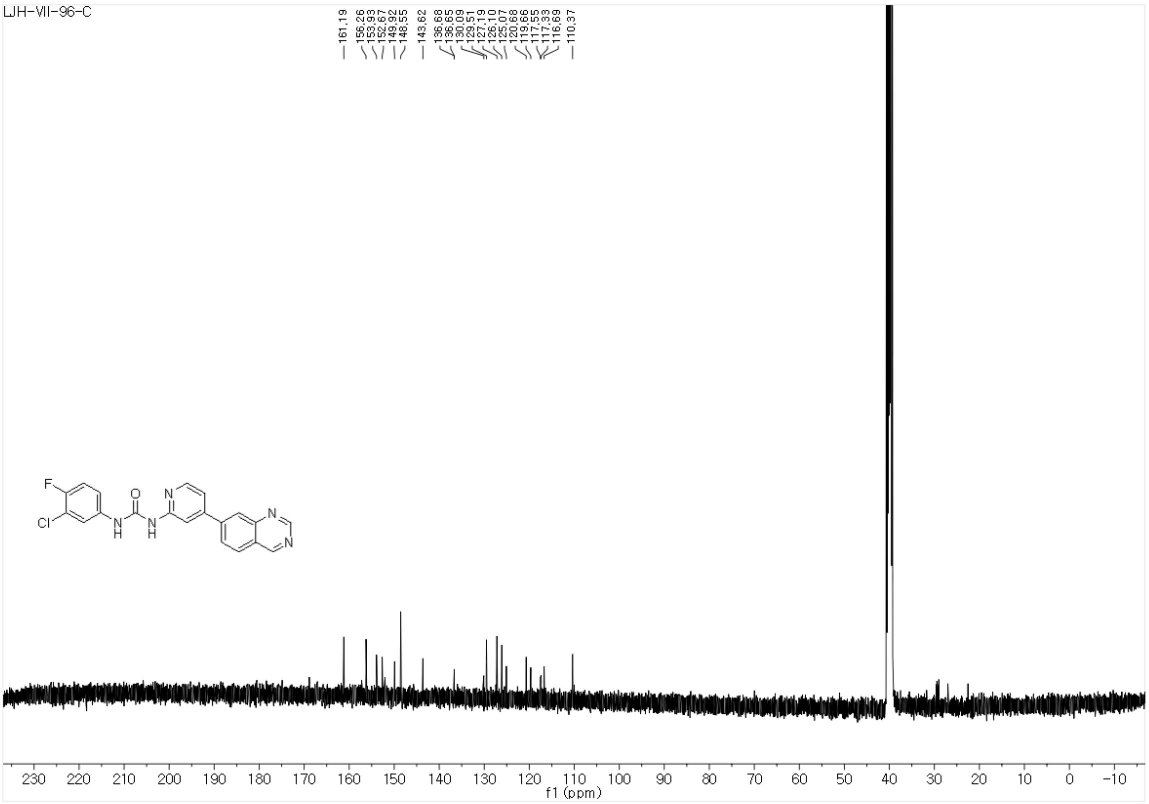

Figure S17. ^{1}H NMR (400 MHz, DMSO-d_6) spectrum of compound **4i**

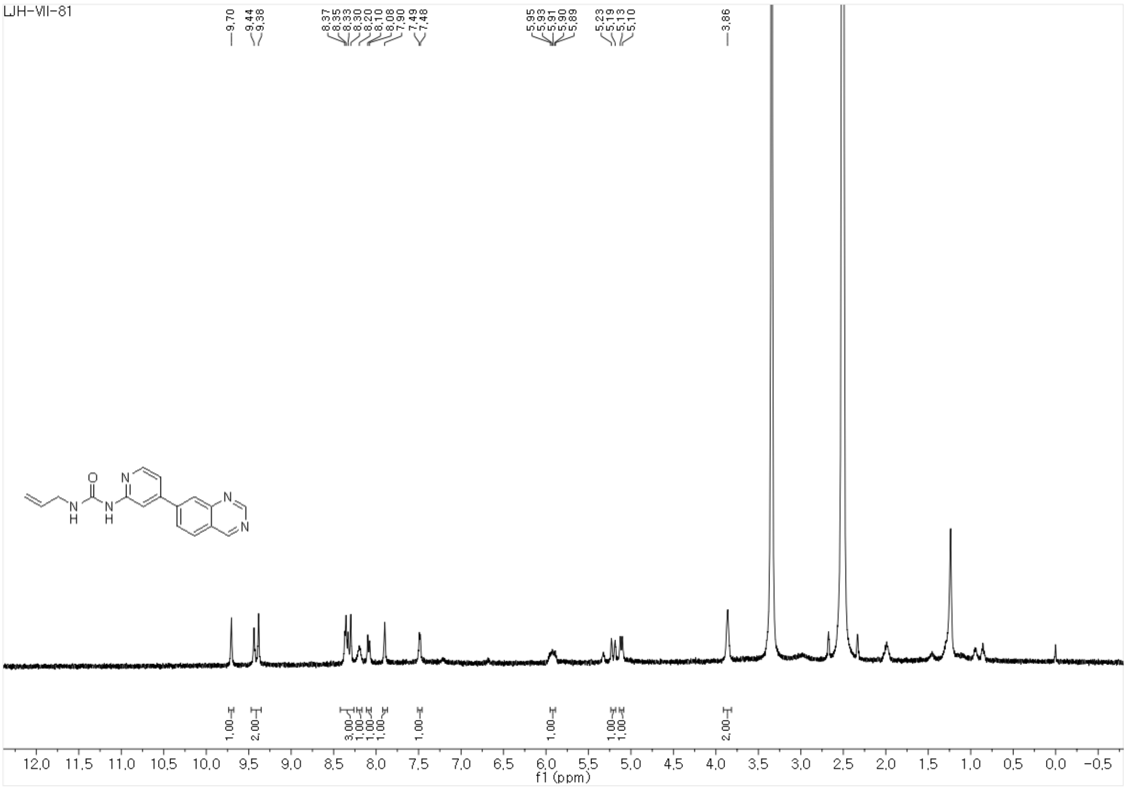

Figure S18. ^{13}C NMR (100 MHz, Chloroform-*d*) spectrum of compound **4i**

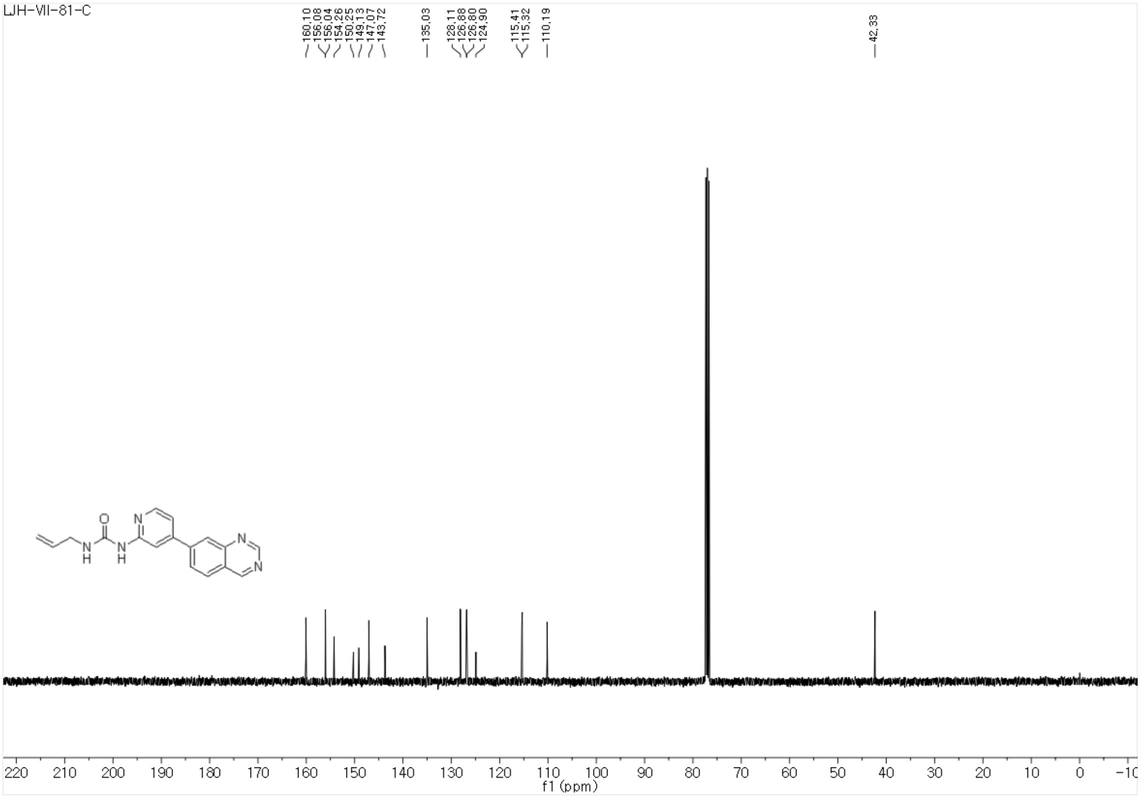

Figure S19. ^{1}H NMR (400 MHz, DMSO-d_6) spectrum of compound **4j**

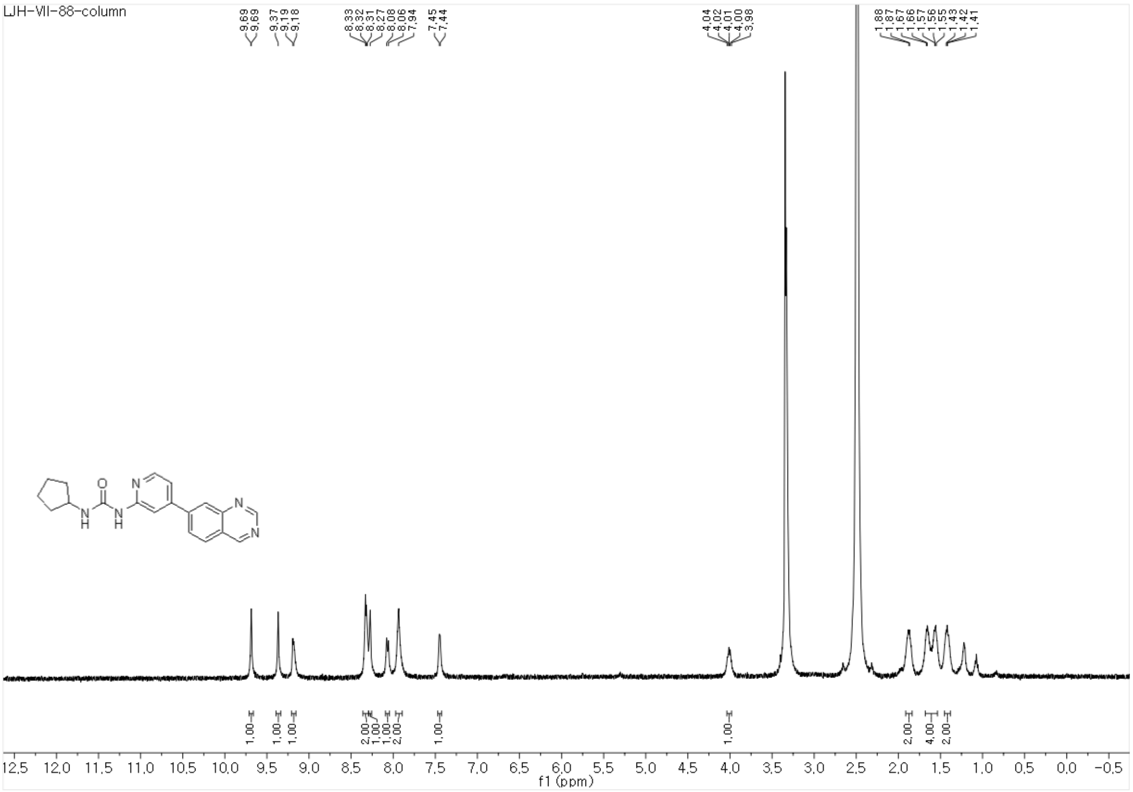

Figure S20. ^{13}C NMR (100 MHz, DMSO-d_6) spectrum of compound **4j**

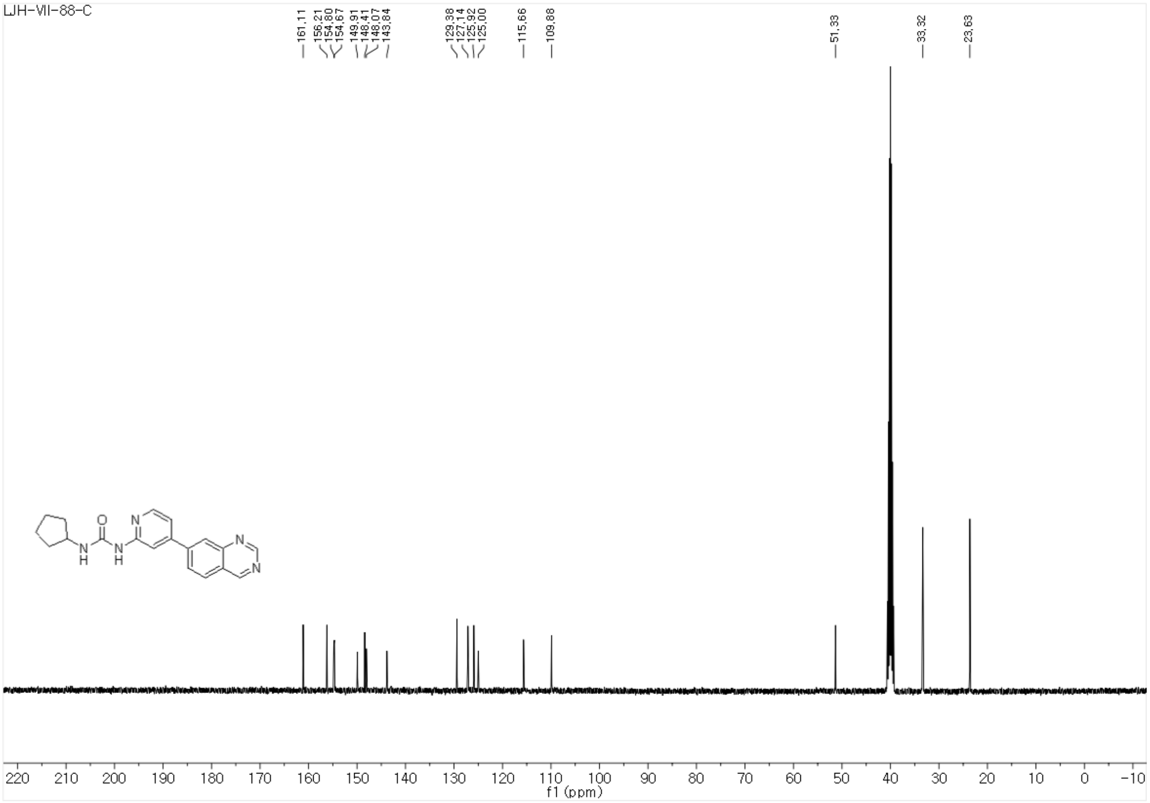

Figure S21. ^{1}H NMR (400 MHz, DMSO-d_6) spectrum of compound **4k**

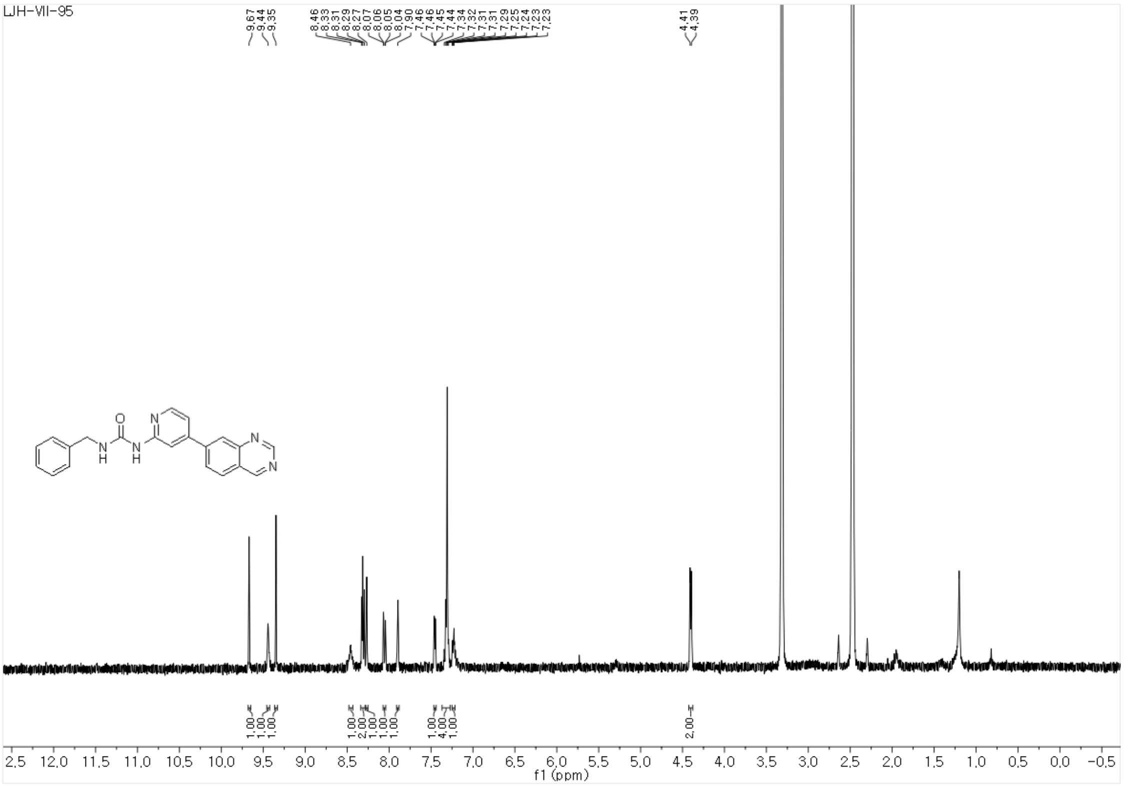

Figure S22. ^{13}C NMR (100 MHz, DMSO-d_6) spectrum of compound **4k**

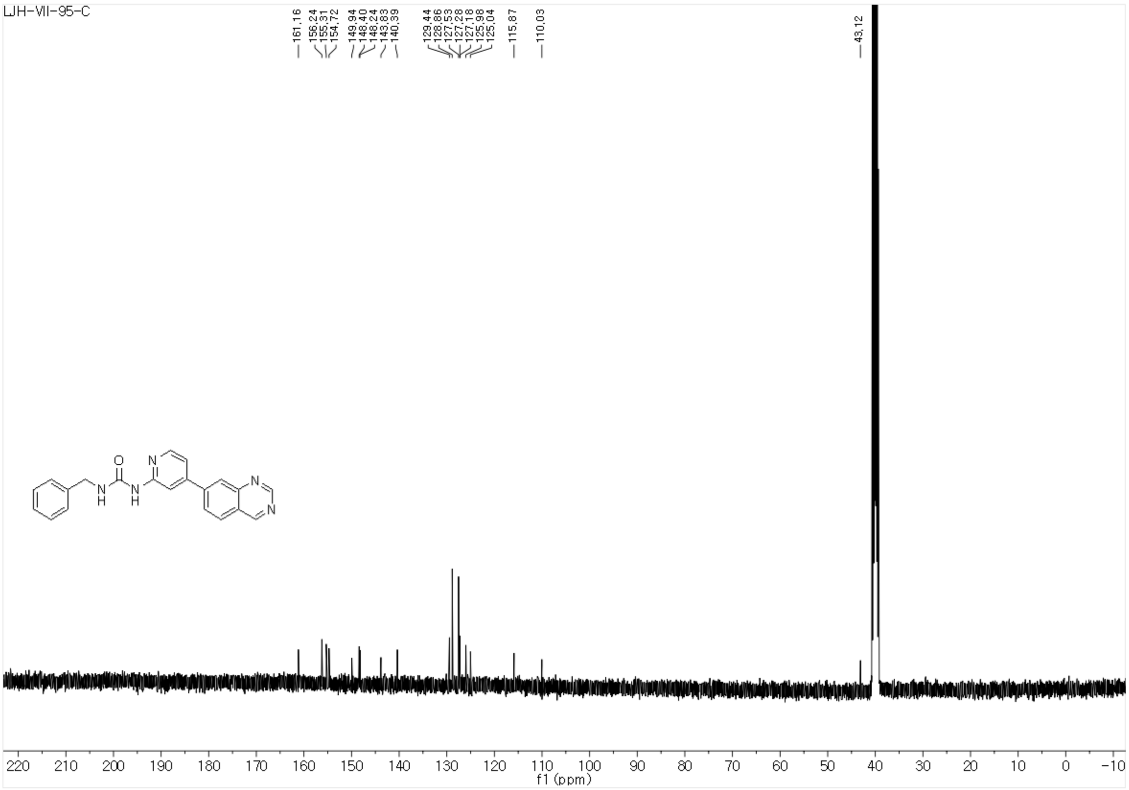

Figure S23. ^{1}H NMR (400 MHz, DMSO-d_6) spectrum of compound **4l**

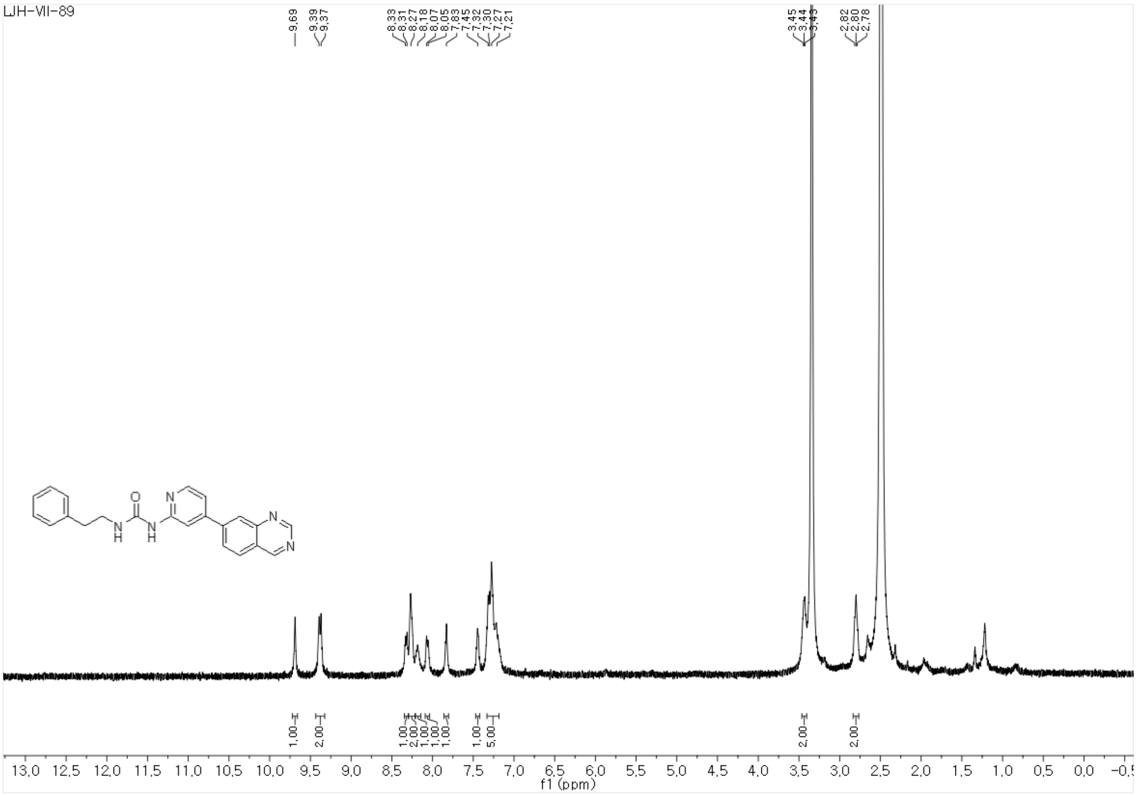

Figure S24. ^{13}C NMR (100 MHz, DMSO-d_6) spectrum of compound **4l**

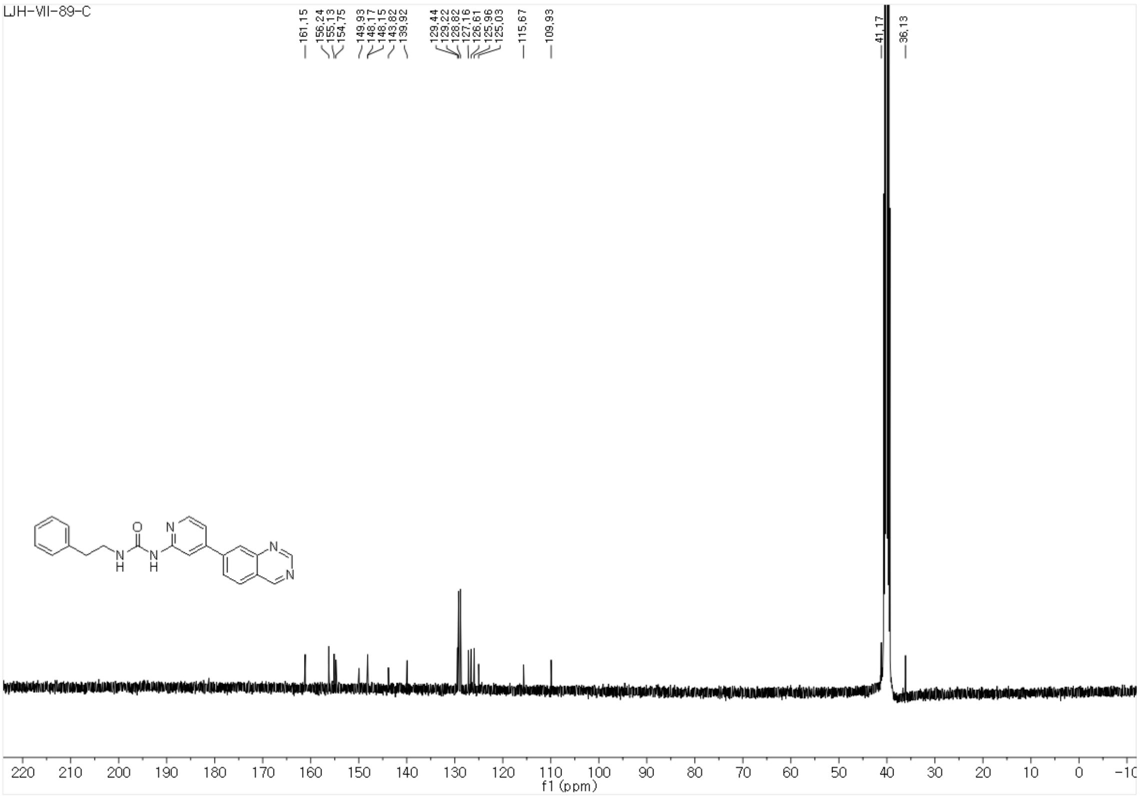

Figure S25. ^{1}H NMR (400 MHz, DMSO-d_6) spectrum of compound **4m**

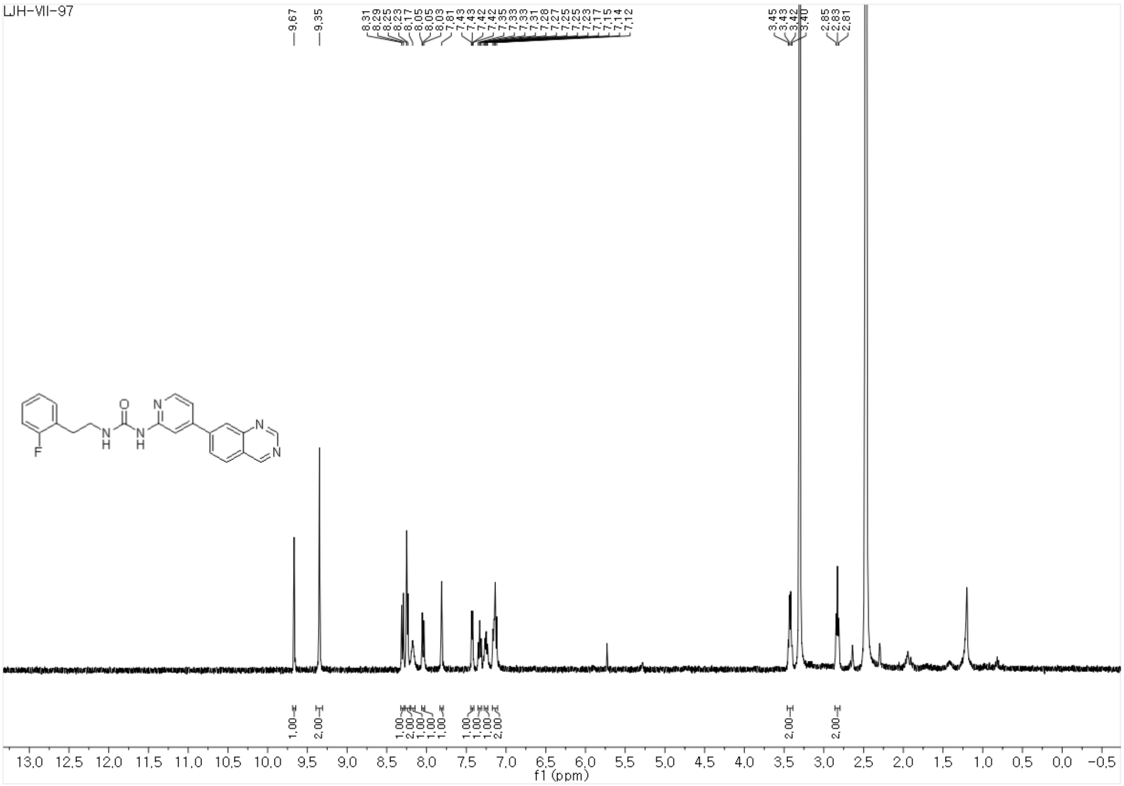

Figure S26. ^{13}C NMR (100 MHz, DMSO-d_6) spectrum of compound **4m**

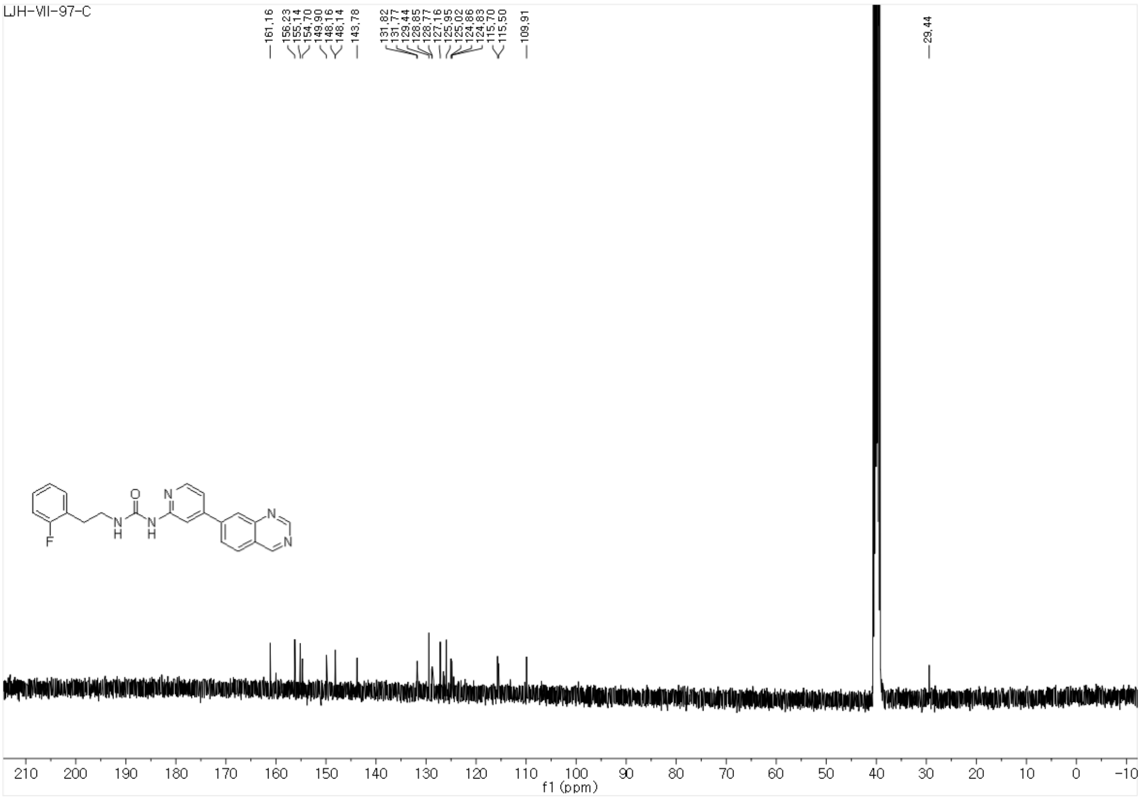

Figure S27. ^{1}H NMR (400 MHz, DMSO-d_6) spectrum of compound **4n**

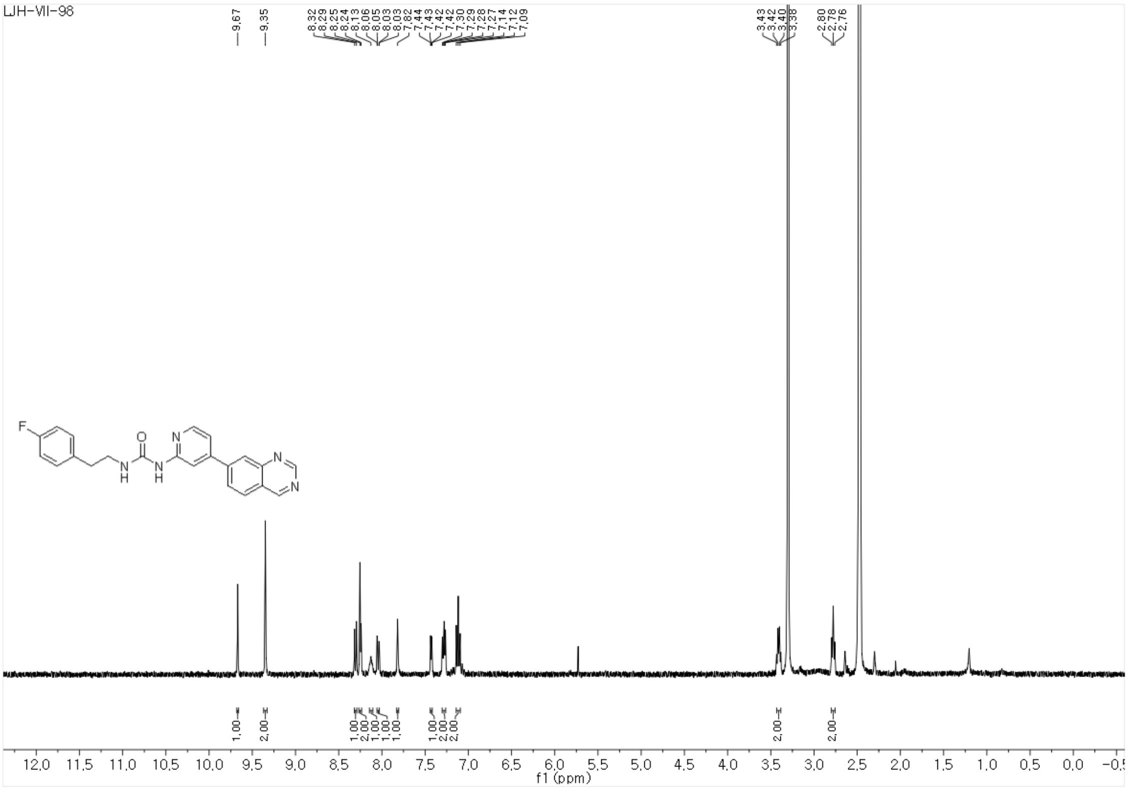

Figure S28. ^{13}C NMR (100 MHz, DMSO-d_6) spectrum of compound **4n**

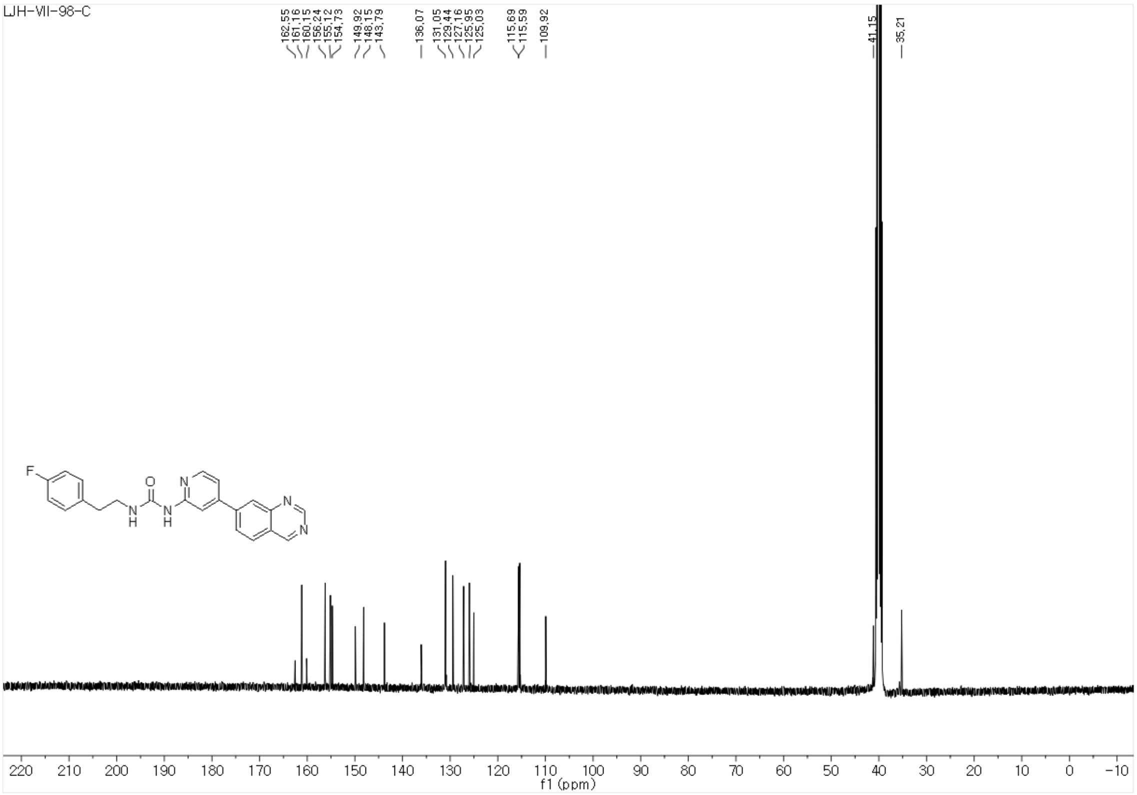

Figure S29. ^{1}H NMR (400 MHz, DMSO-d_6) spectrum of compound **8a**

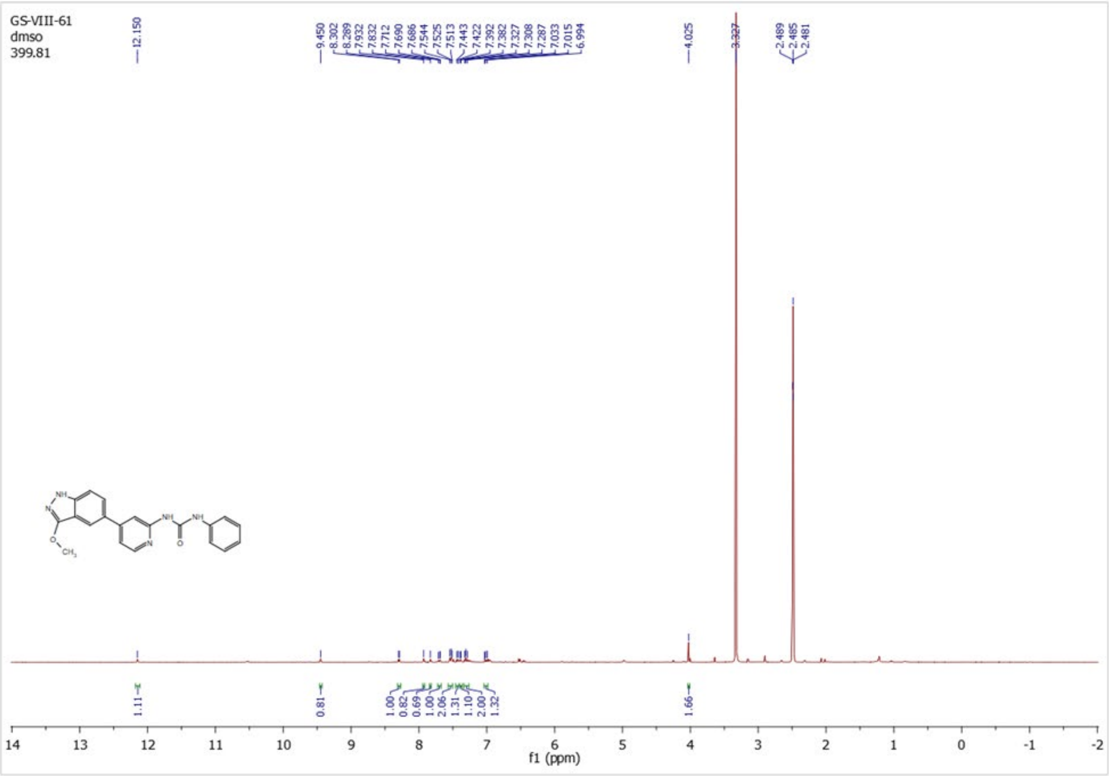

Figure S30. ^{13}C NMR (100 MHz, DMSO-d_6) spectrum of compound **8a**

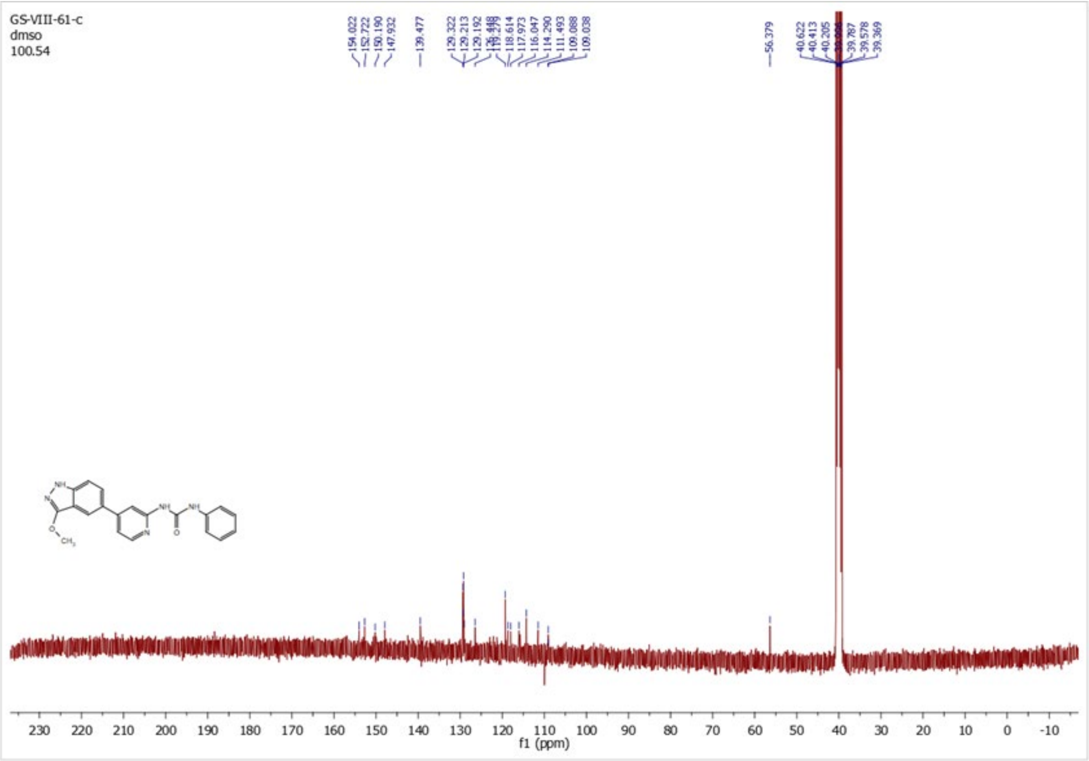

Figure S31. ^{1}H NMR (400 MHz, DMSO-d_6) spectrum of compound **8b**

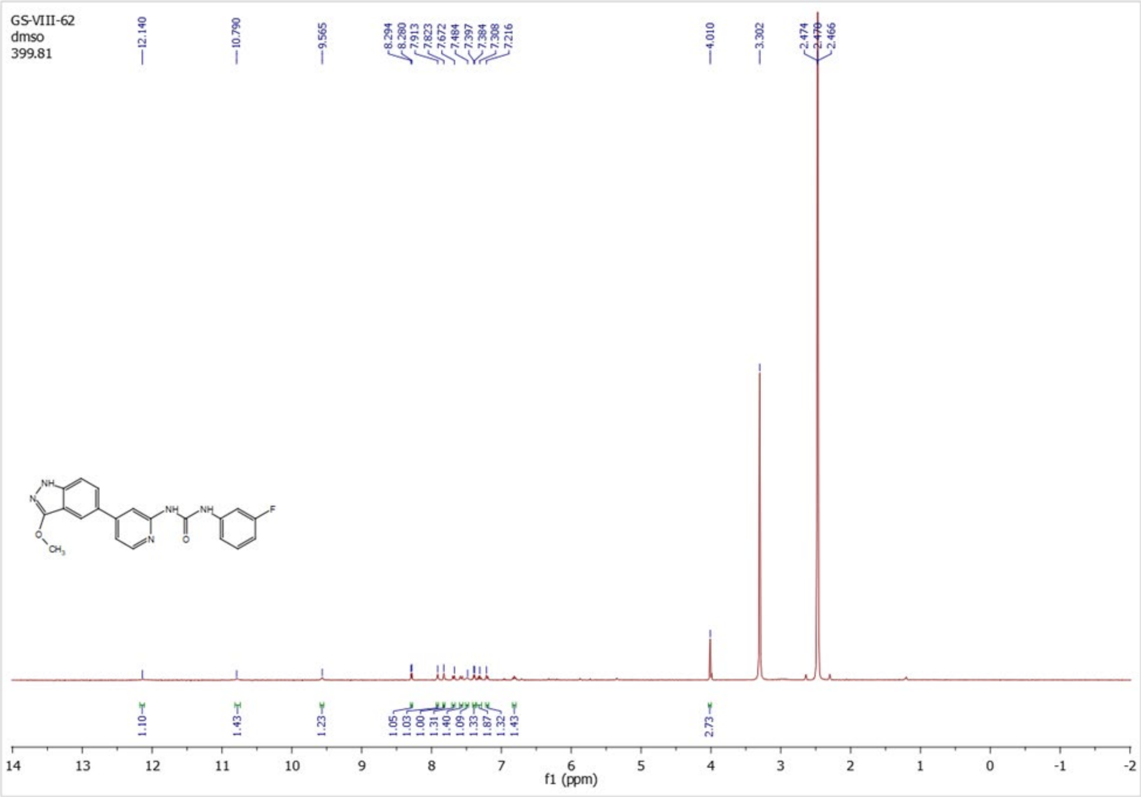

Figure S32. ^{13}C NMR (100 MHz, DMSO-d_6) spectrum of compound **8b**

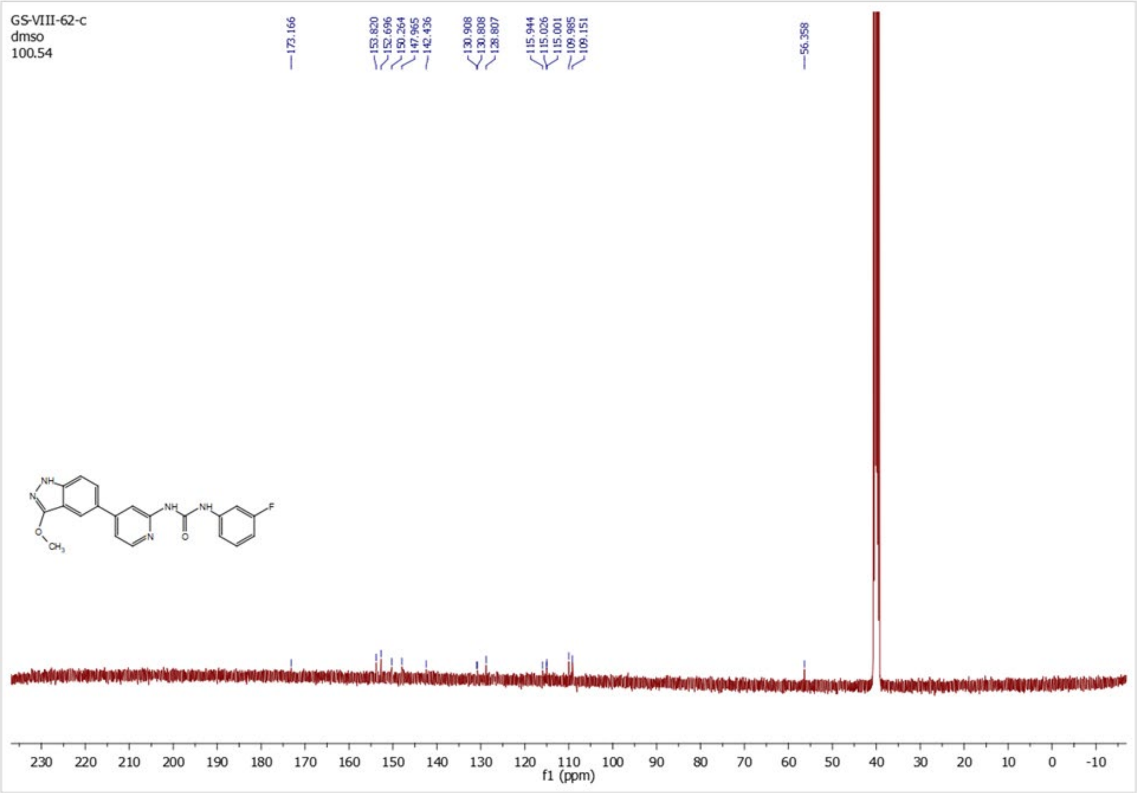

Figure S33. ^{1}H NMR (400 MHz, DMSO-d_6) spectrum of compound **8c**

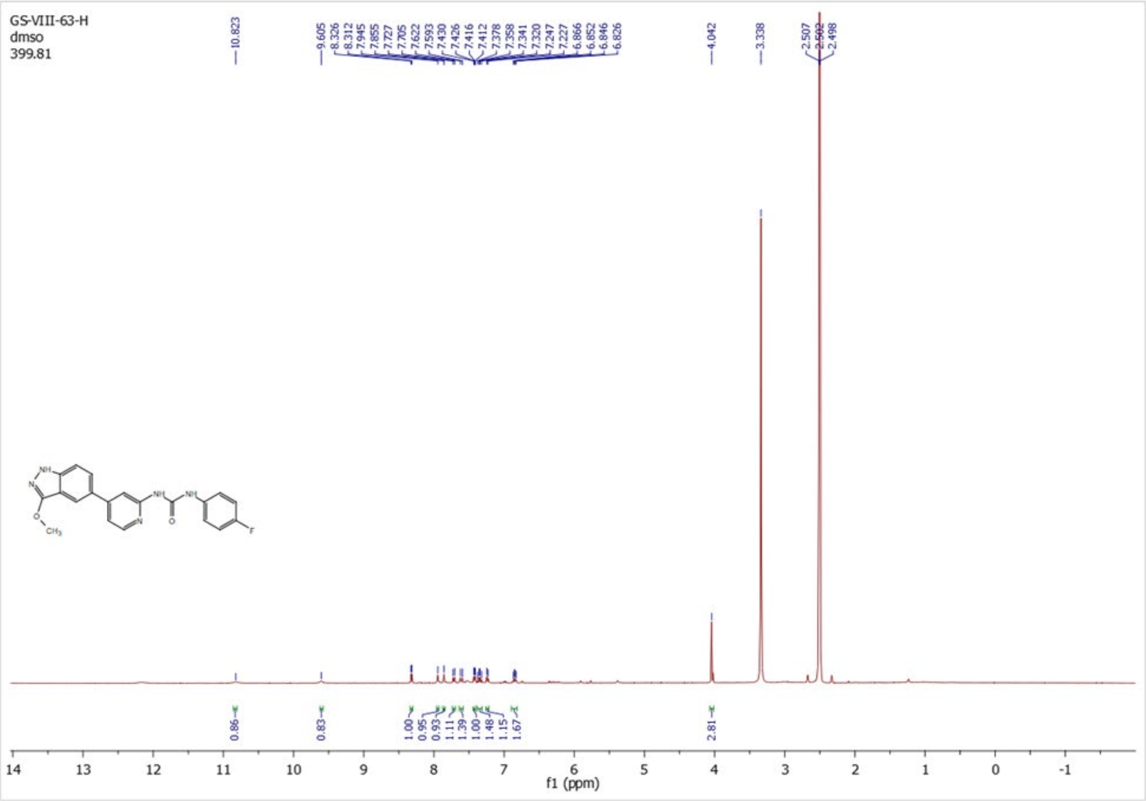

Figure S34. ^{13}C NMR (100 MHz, DMSO-d_6) spectrum of compound **8c**

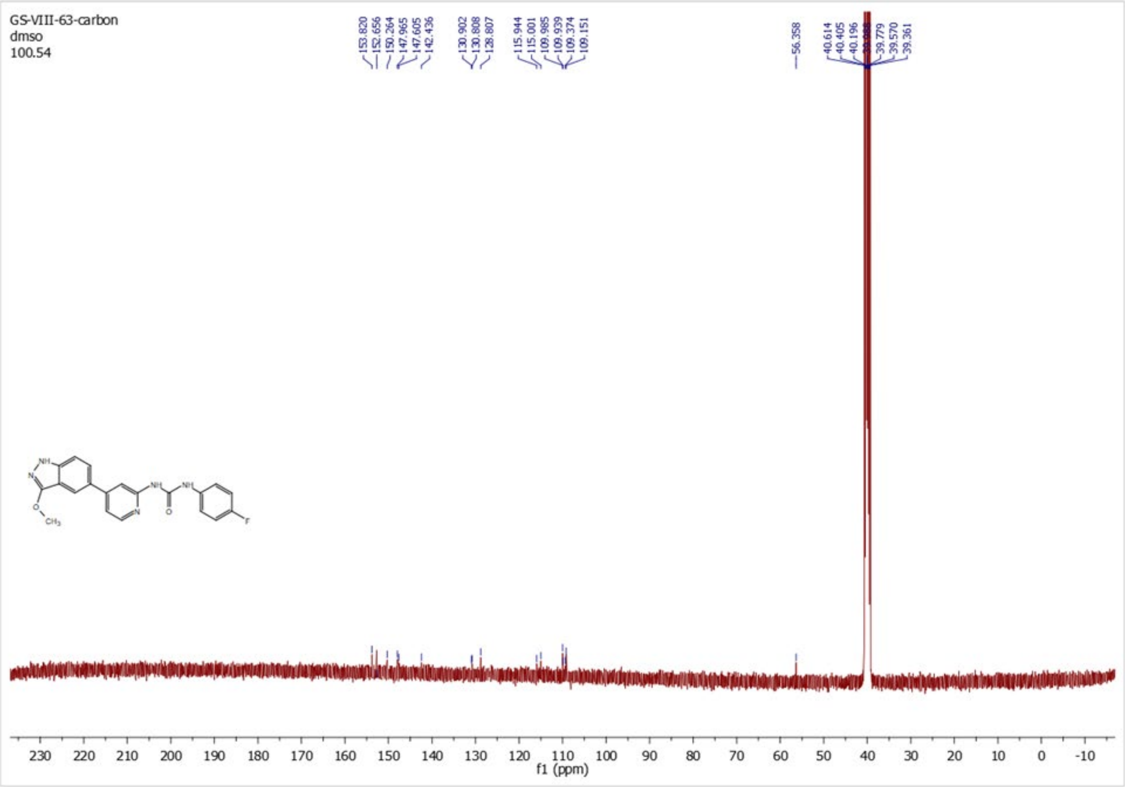

Figure S35. ^{1}H NMR (400 MHz, DMSO-d_6) spectrum of compound **8d**

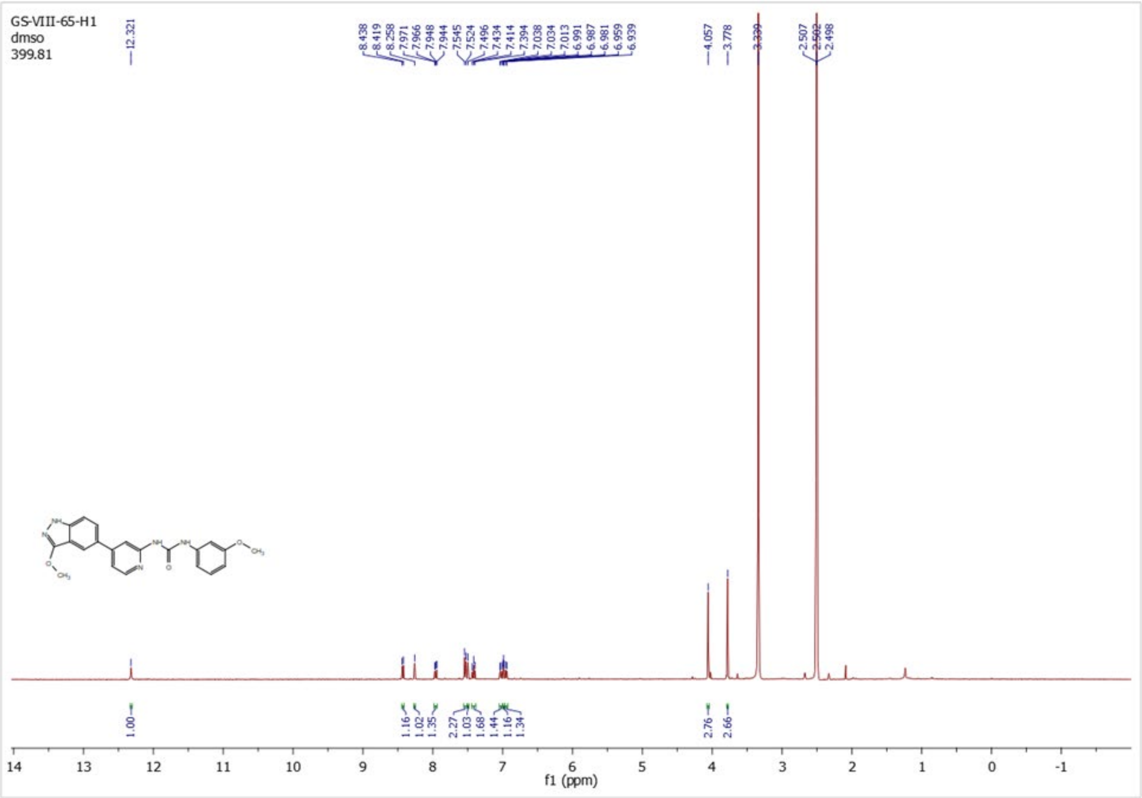

Figure S36. ^{13}C NMR (100 MHz, DMSO-d_6) spectrum of compound **8d**

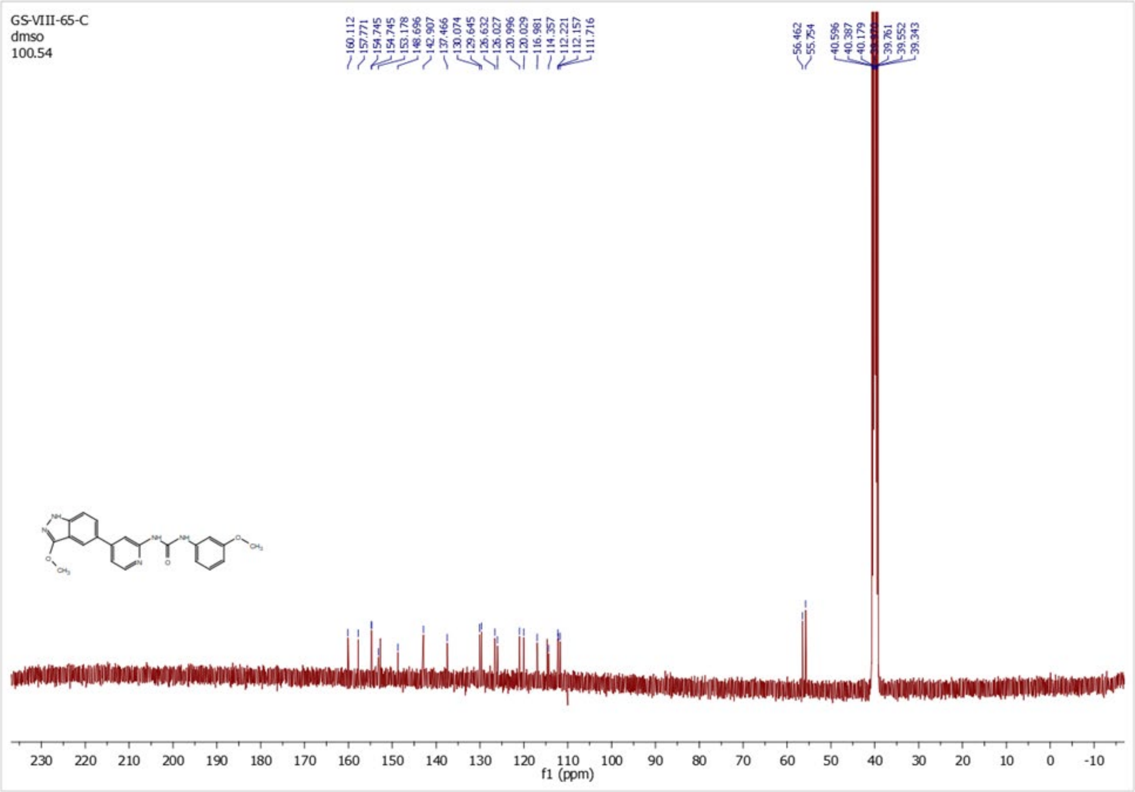

Figure S37. ^{1}H NMR (400 MHz, DMSO-d_6) spectrum of compound **8e**

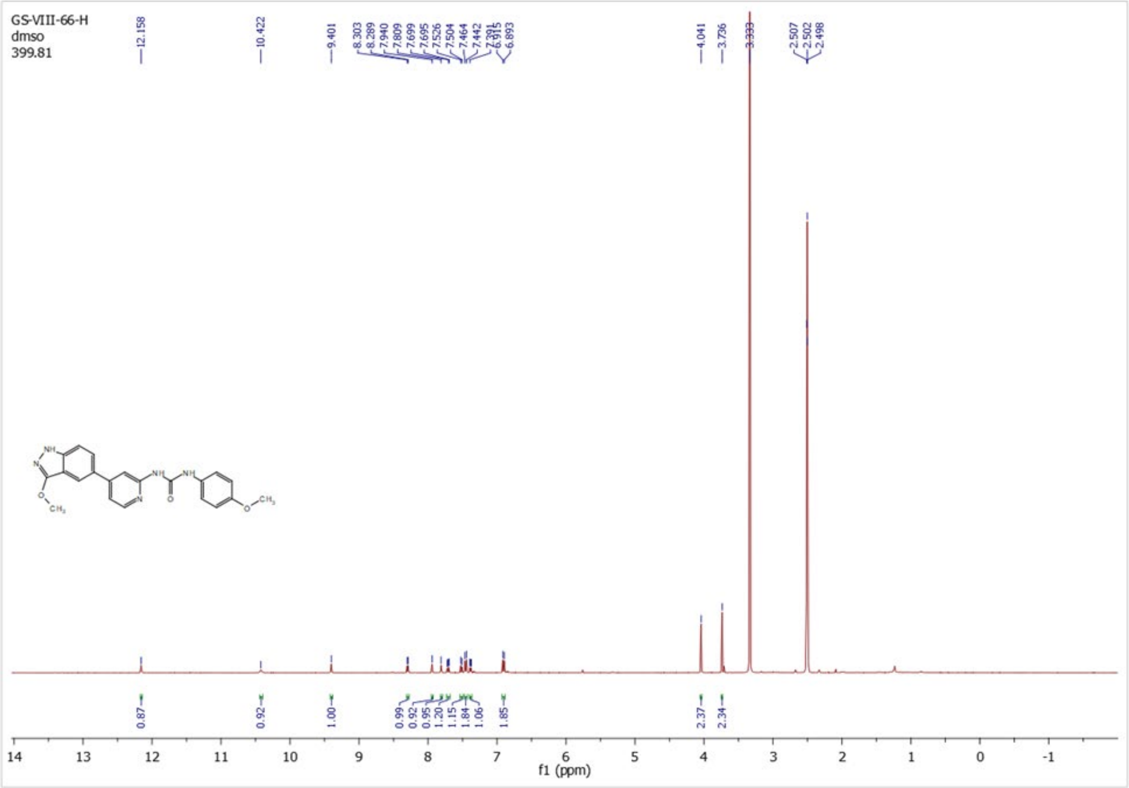

Figure S38. ^{13}C NMR (100 MHz, DMSO-d_6) spectrum of compound **8e**

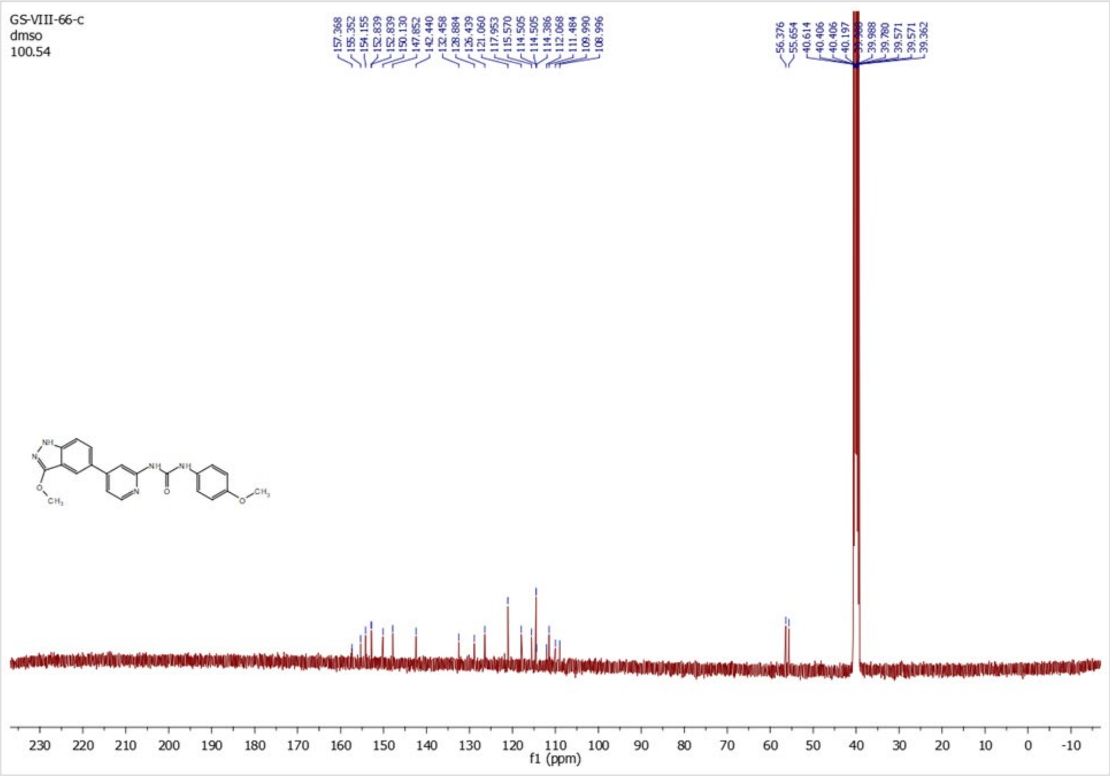

Figure S39. ^{1}H NMR (400 MHz, DMSO-d_6) spectrum of compound **8f**

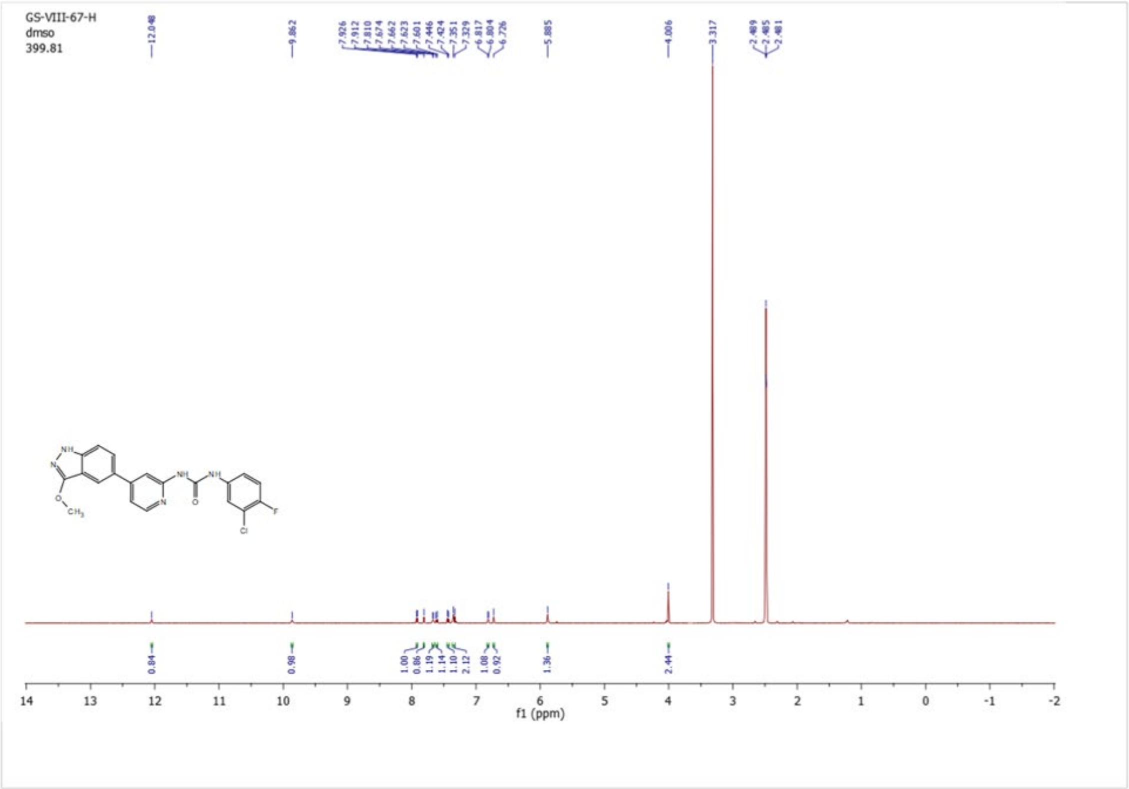

Figure S40. ^{13}C NMR (100 MHz, DMSO-d_6) spectrum of compound **8f**

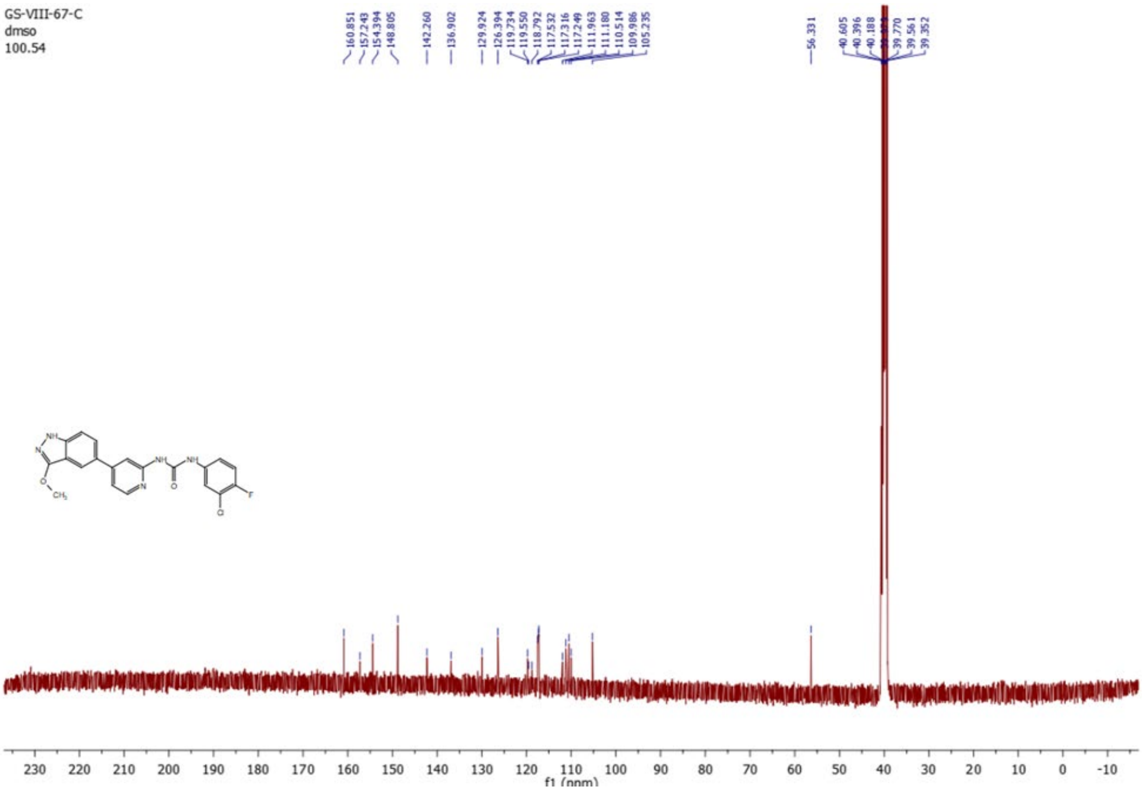

Figure S41. ^{1}H NMR (400 MHz, DMSO-d_6) spectrum of compound **8g**

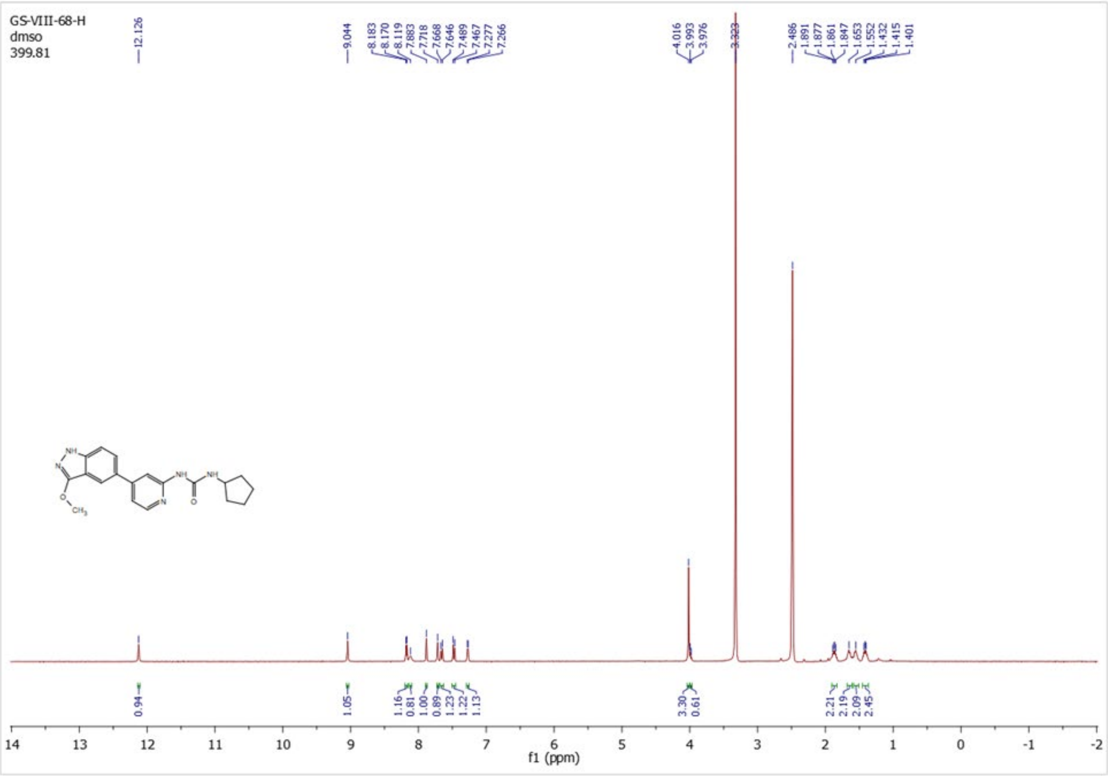

Figure S42. ^{13}C NMR (100 MHz, DMSO-d_6) spectrum of compound **8g**

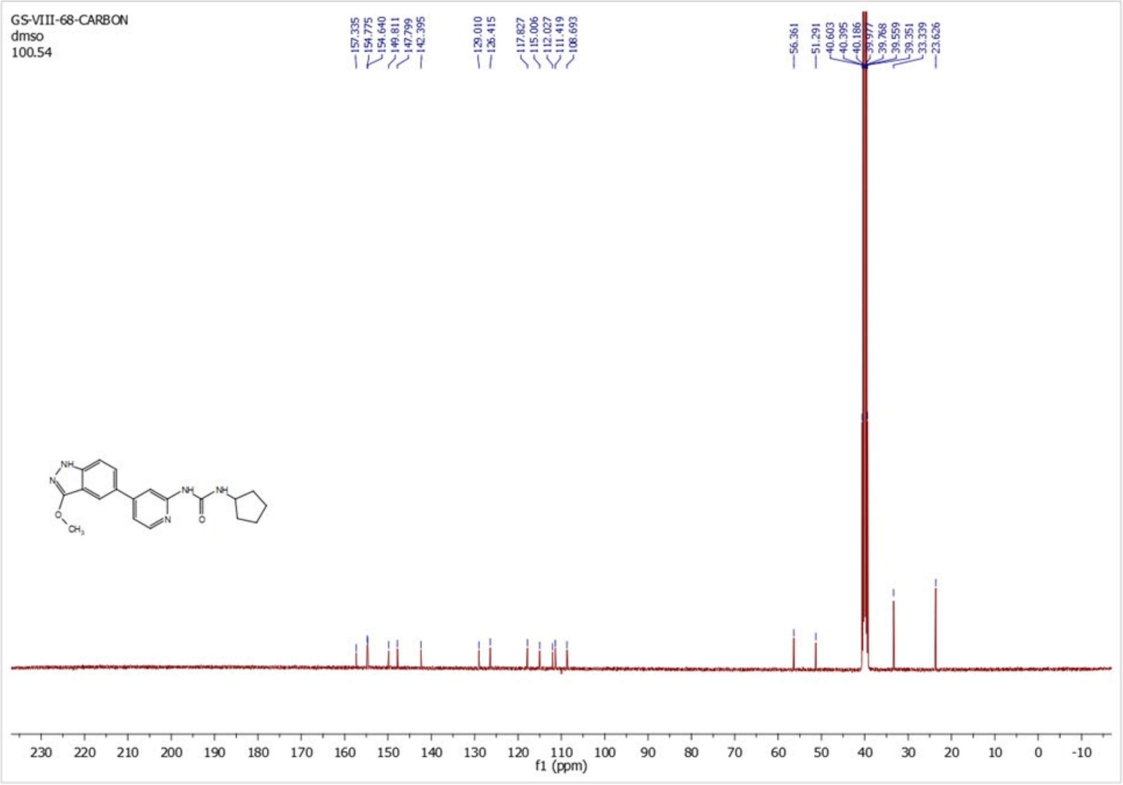

Figure S43. ^{1}H NMR (400 MHz, DMSO-d_6) spectrum of compound **8h**

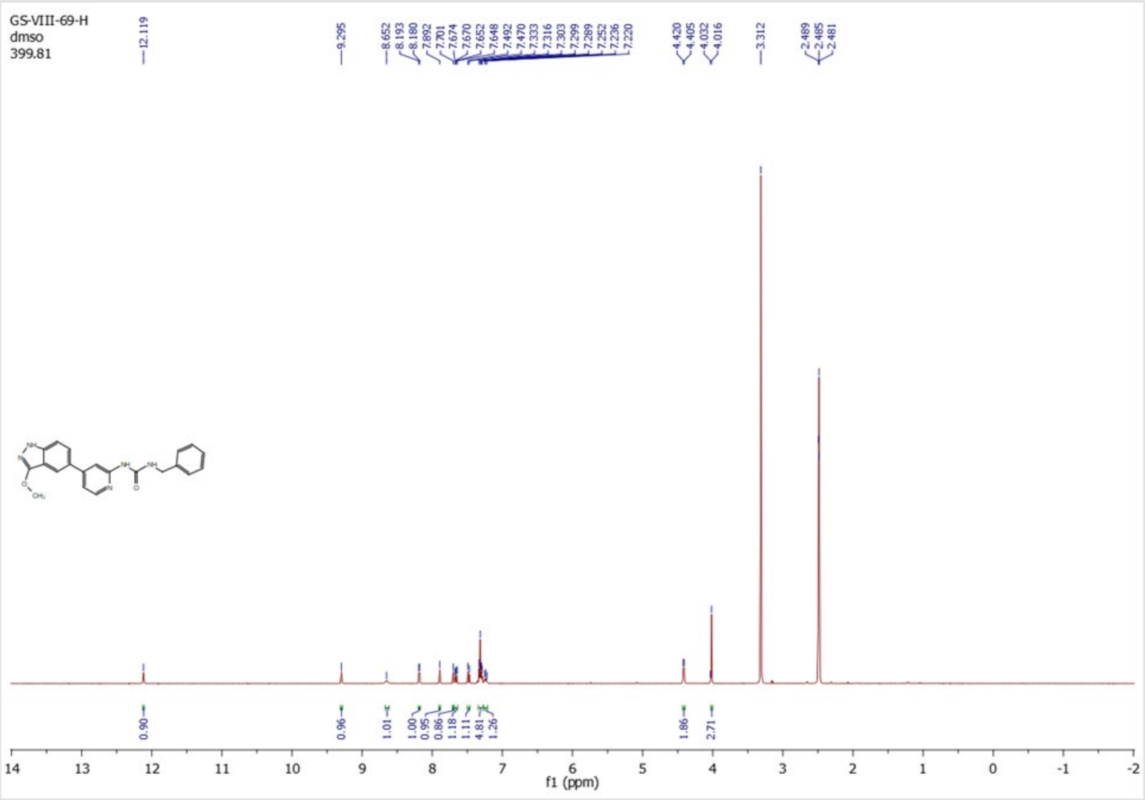

Figure S44. ^{13}C NMR (100 MHz, DMSO-d_6) spectrum of compound **8h**

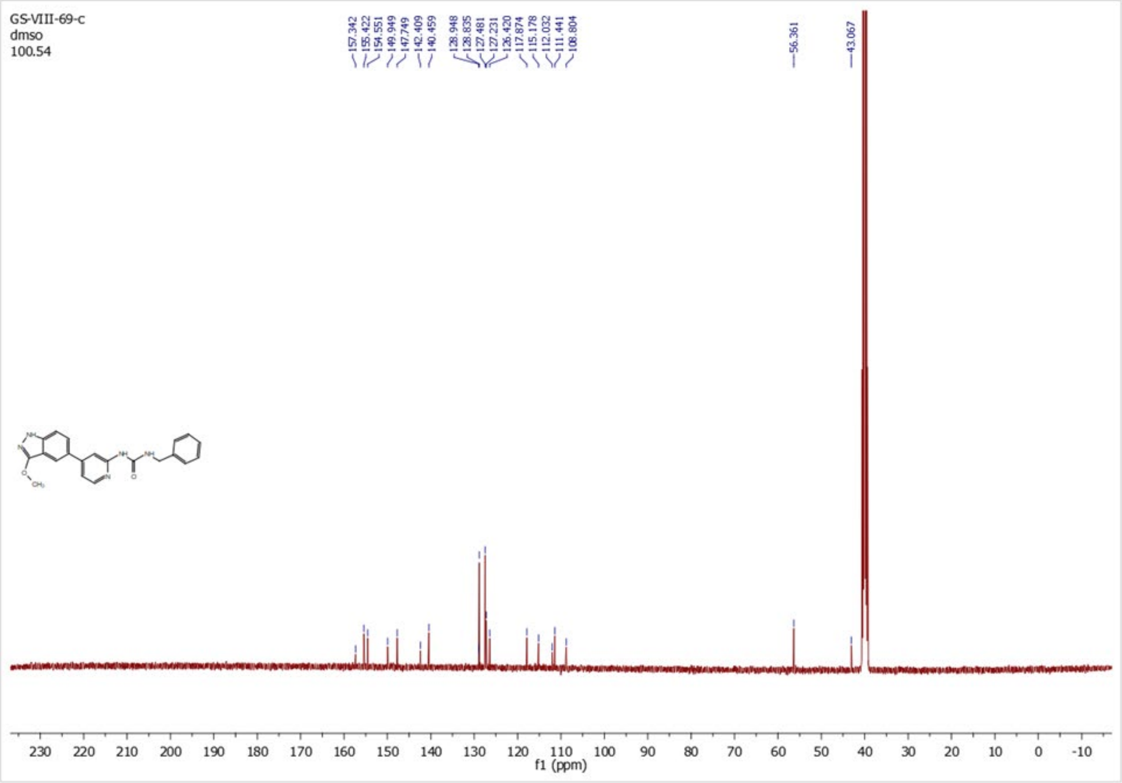

Figure S45. ^{1}H NMR (400 MHz, DMSO-d_6) spectrum of compound **8i**

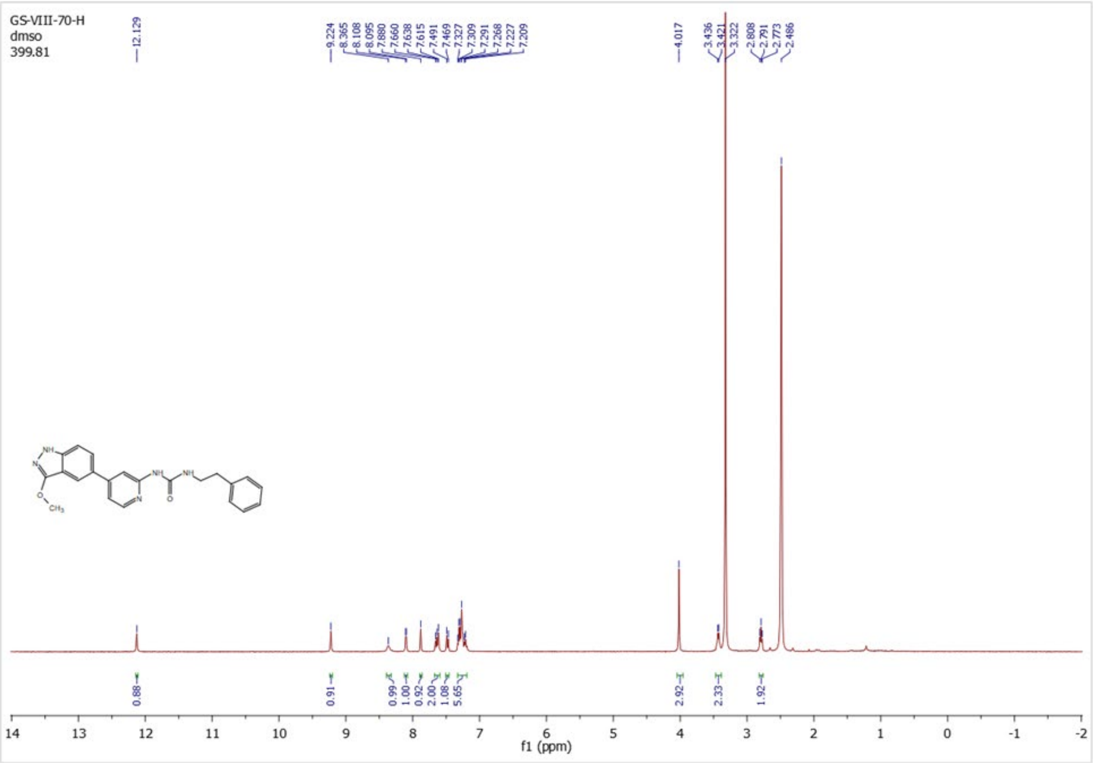

Figure S46. ^{13}C NMR (100 MHz, DMSO-d_6) spectrum of compound **8i**

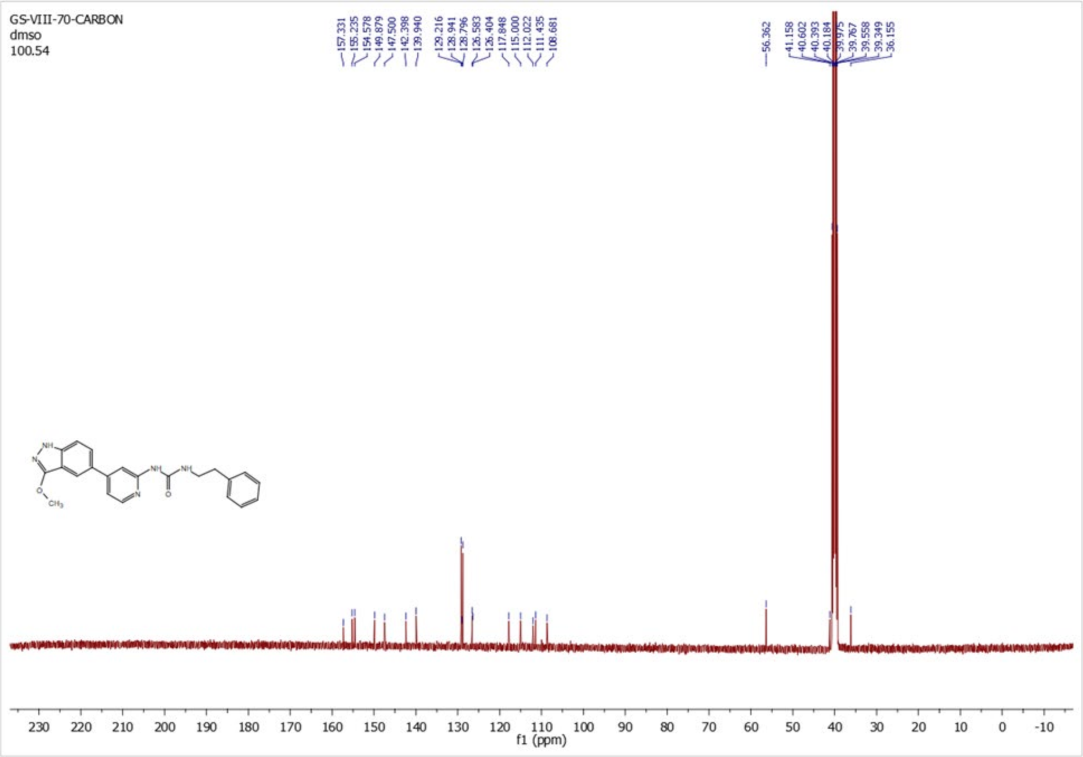

Figure S47. ^{1}H NMR (400 MHz, DMSO-d_6) spectrum of compound **8j**

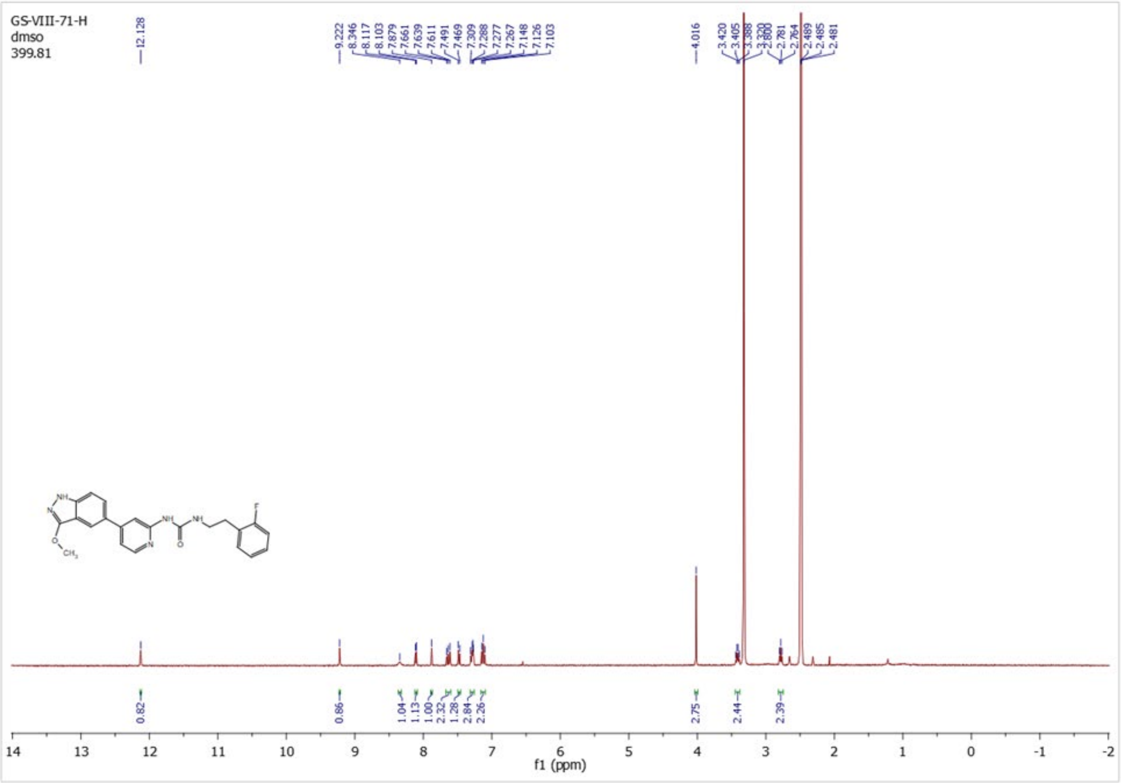

Figure S48. ^{13}C NMR (100 MHz, DMSO-d_6) spectrum of compound **8j**

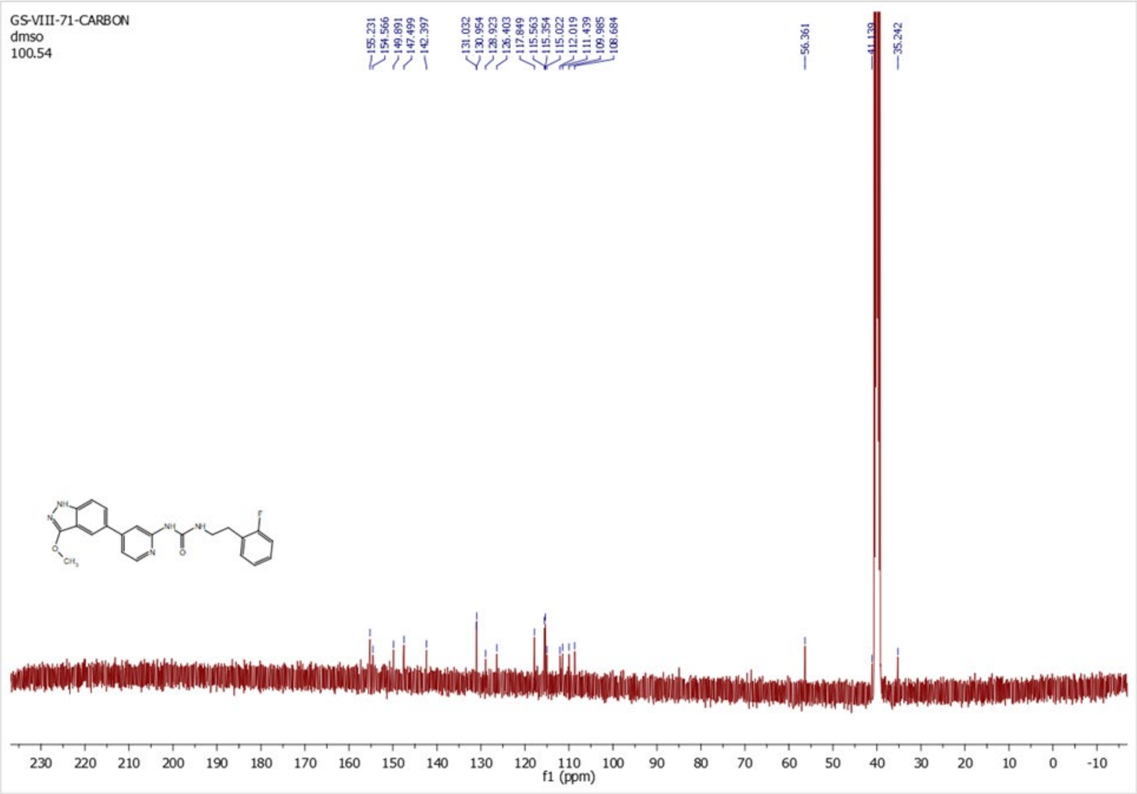

Figure S49. ^{1}H NMR (400 MHz, DMSO-d_6) spectrum of compound **8k**

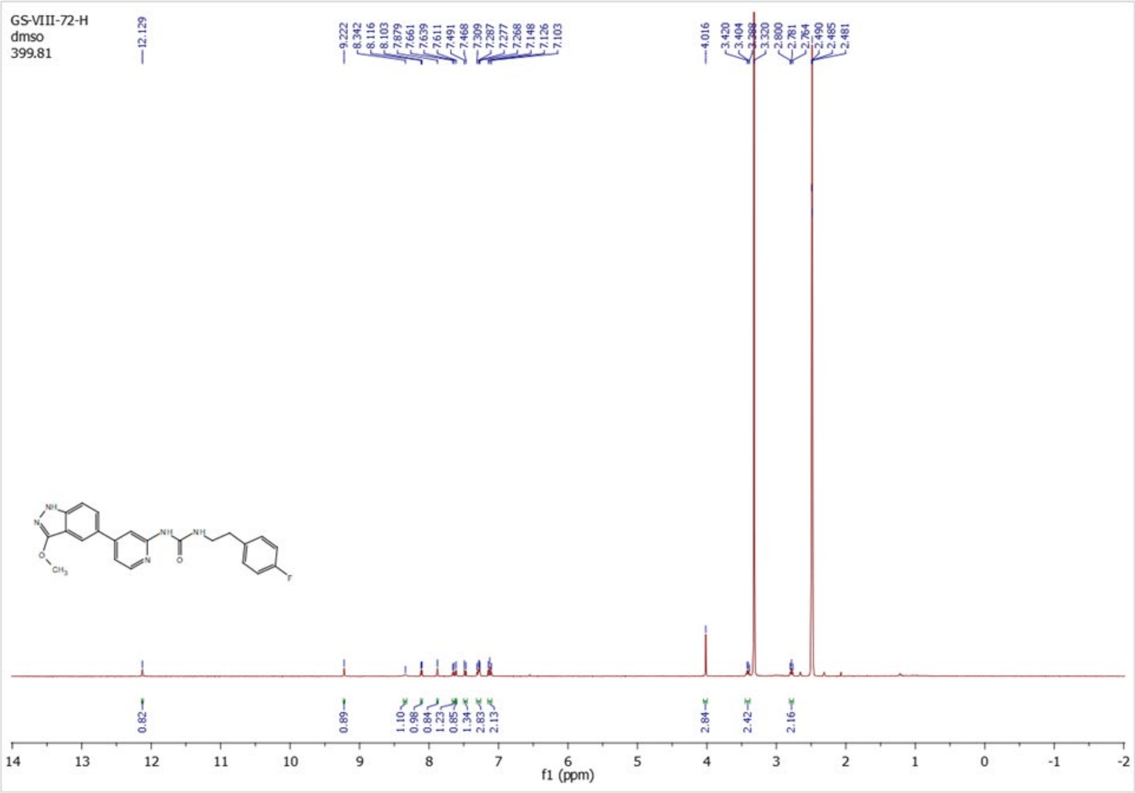

Figure S50. ^{13}C NMR (100 MHz, DMSO-d_6) spectrum of compound **8k**

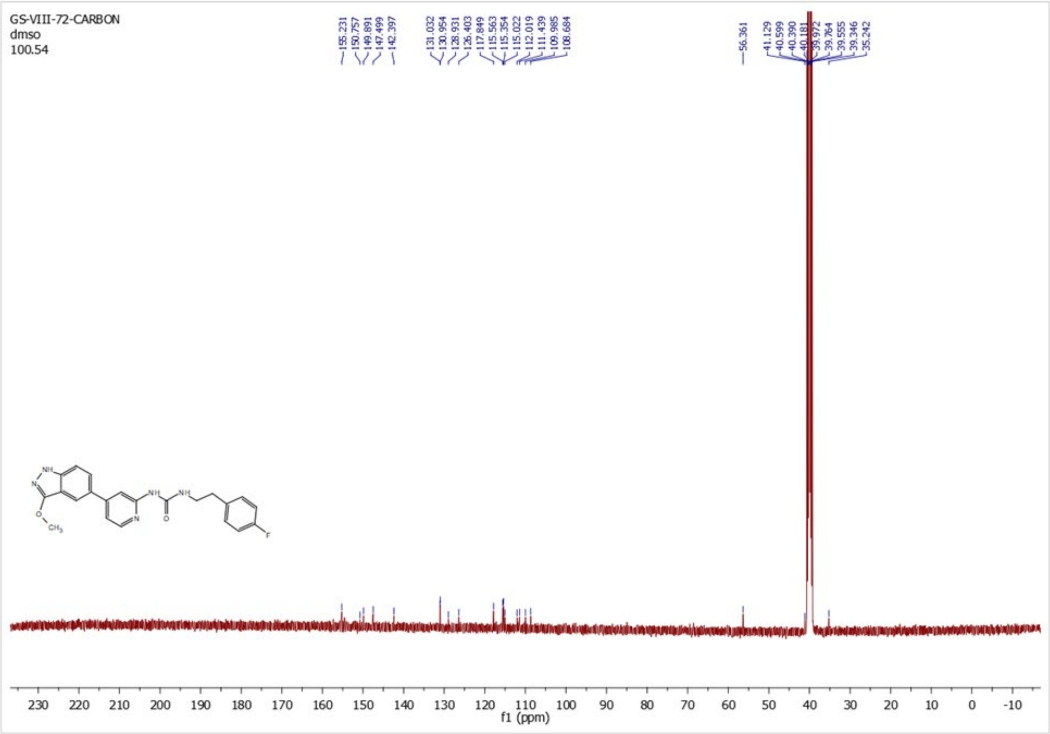

Figure S51. HPLC Analysis

Compound 4a

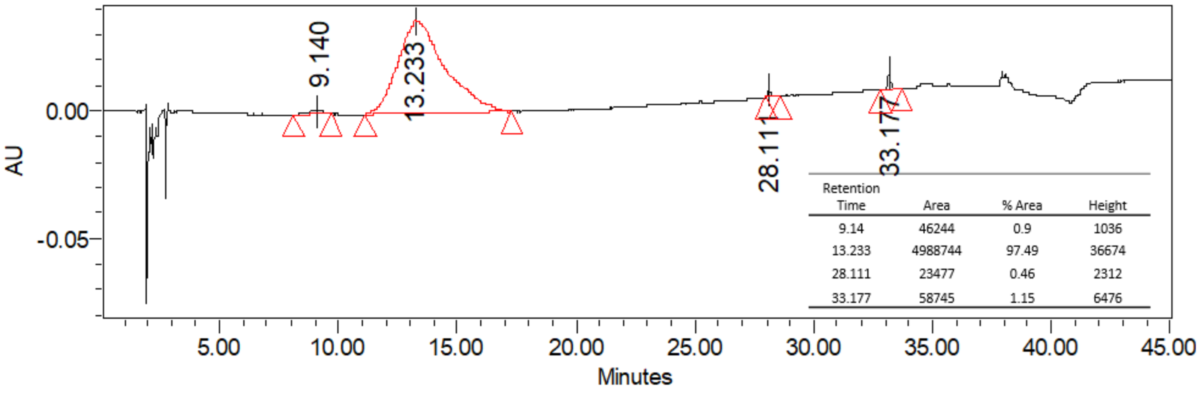

Retention Time	Area	% Area	Height
9.14	46244	0.9	1036
13.233	4988744	97.49	36674
28.111	23477	0.46	2312
33.177	58745	1.15	6476

Compound 4b

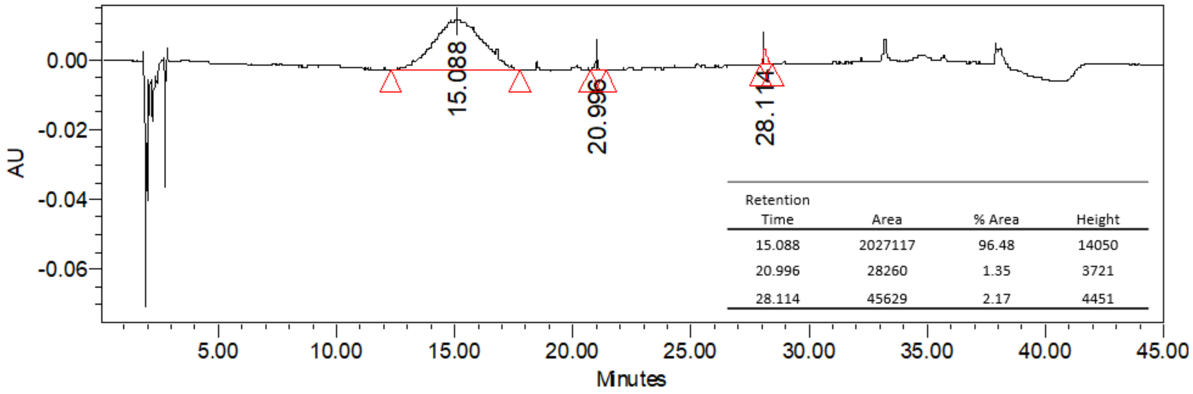

Retention Time	Area	% Area	Height
15.088	2027117	96.48	14050
20.996	28260	1.35	3721
28.114	45629	2.17	4451

Compound 4c

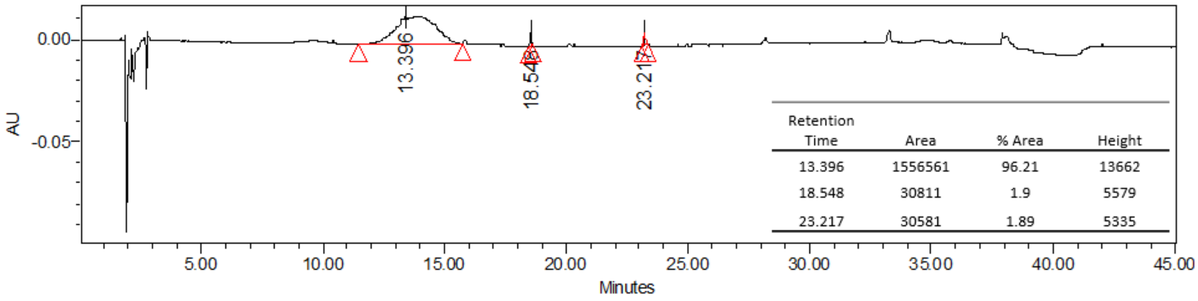

Retention Time	Area	% Area	Height
13.396	1556561	96.21	13662
18.548	30811	1.9	5579
23.217	30581	1.89	5335

Figure S52. HPLC Analysis

Compound 4d

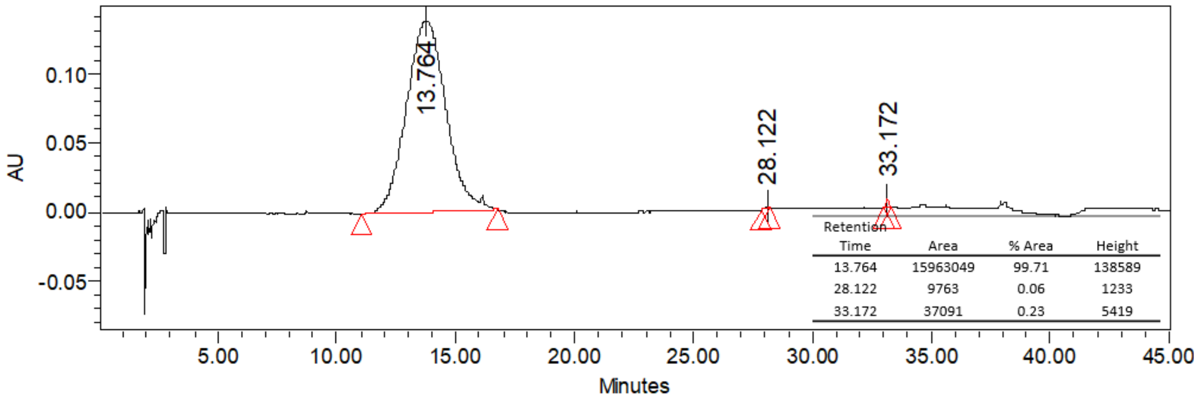

Compound 4e

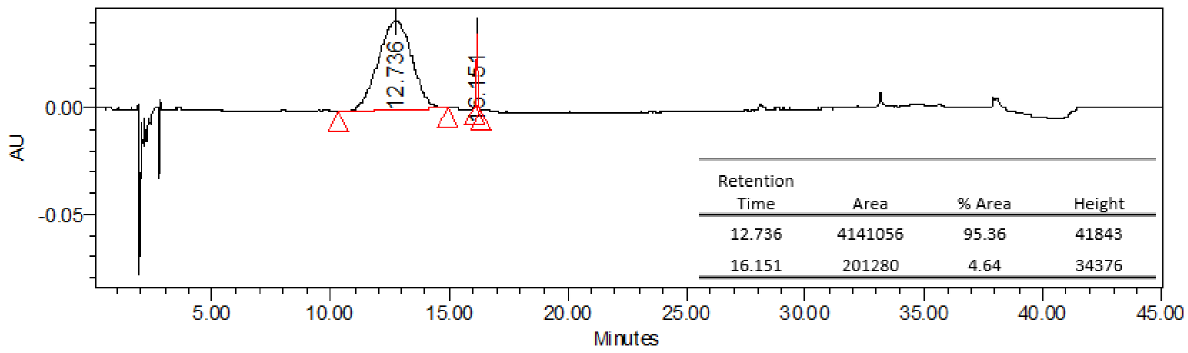

Compound 4f

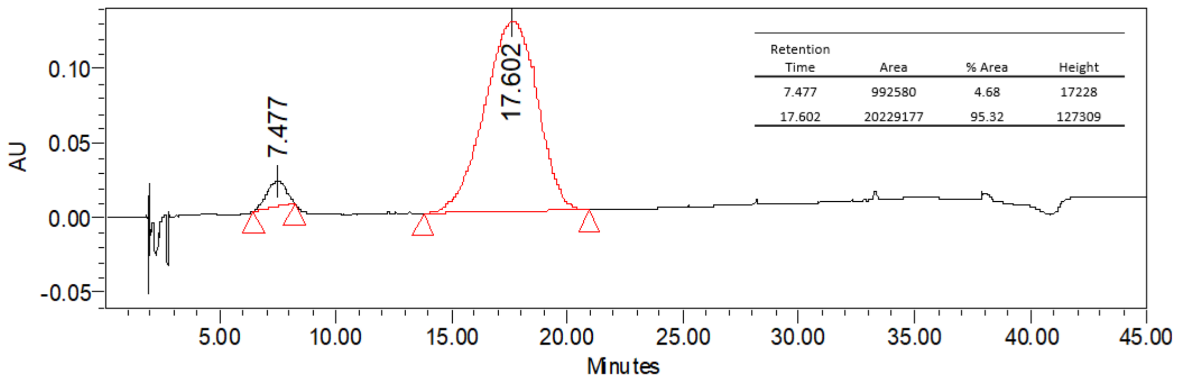

Figure S53. HPLC Analysis

Compound 4g

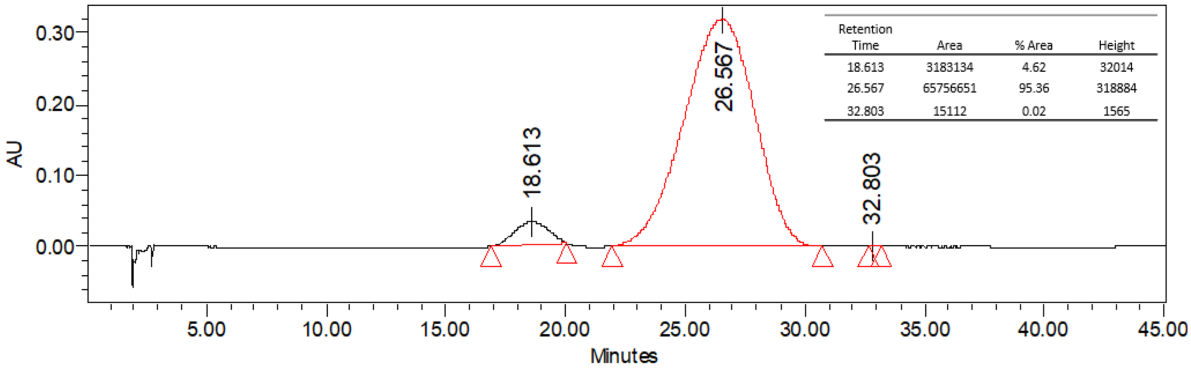

Retention Time	Area	% Area	Height
18.613	3183134	4.62	32014
26.567	65756651	95.36	318884
32.803	15112	0.02	1565

Compound 4h

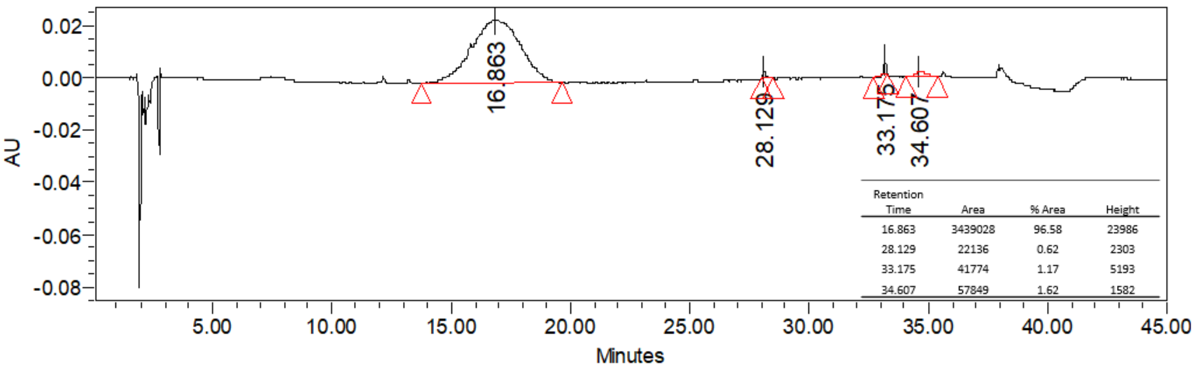

Retention Time	Area	% Area	Height
16.863	3439028	96.58	23986
28.129	22136	0.62	2303
33.175	41774	1.17	5193
34.607	57849	1.62	1582

Compound 4i

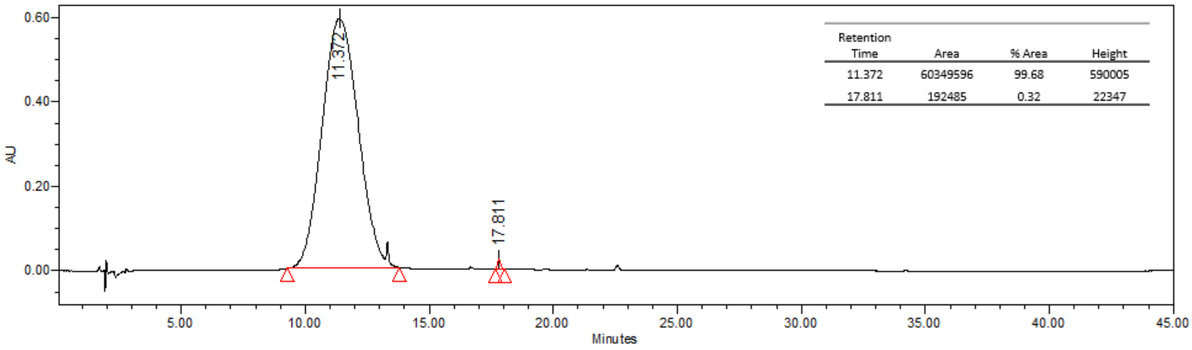

Retention Time	Area	% Area	Height
11.372	60349596	99.68	590005
17.811	192485	0.32	22347

Figure S54. HPLC Analysis

Compound 4j

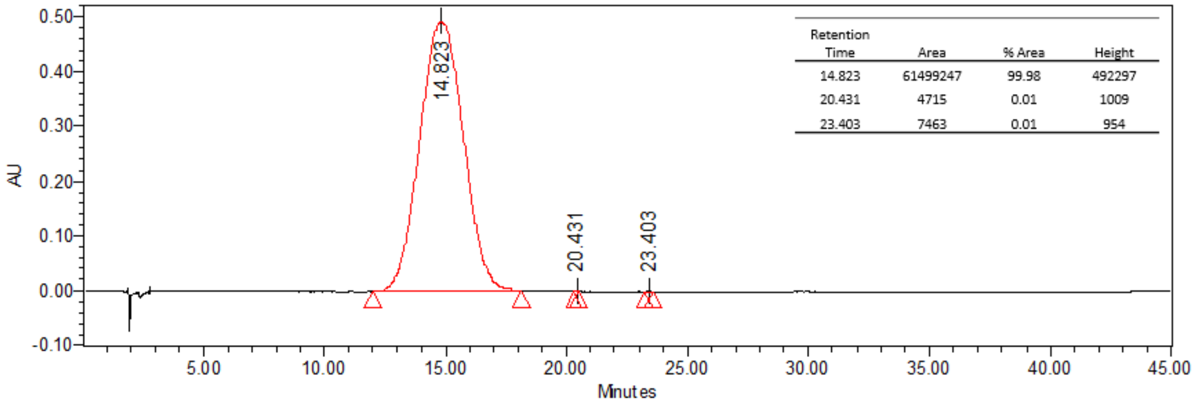

Retention Time	Area	% Area	Height
14.823	61499247	99.98	492297
20.431	4715	0.01	1009
23.403	7463	0.01	954

Compound 4k

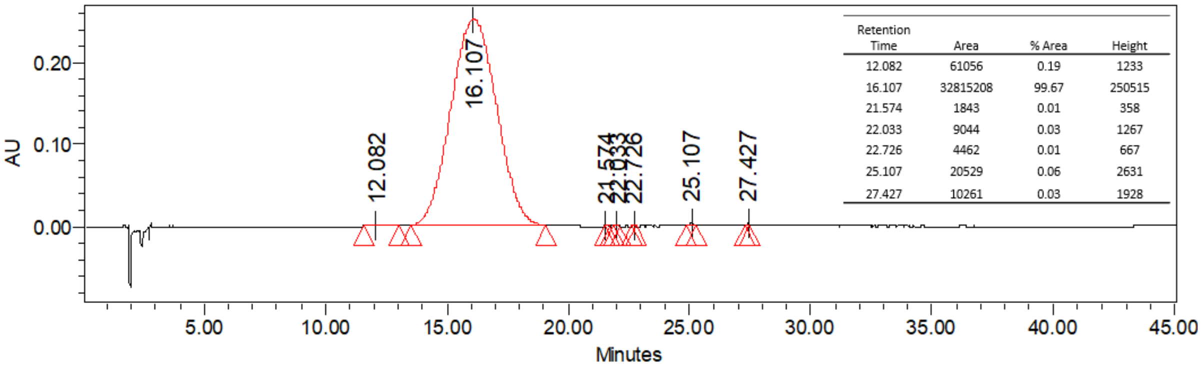

Retention Time	Area	% Area	Height
12.082	61056	0.19	1233
16.107	32815208	99.67	250515
21.574	1843	0.01	358
22.033	9044	0.03	1267
22.726	4462	0.01	667
25.107	20529	0.06	2631
27.427	10261	0.03	1928

Compound 4l

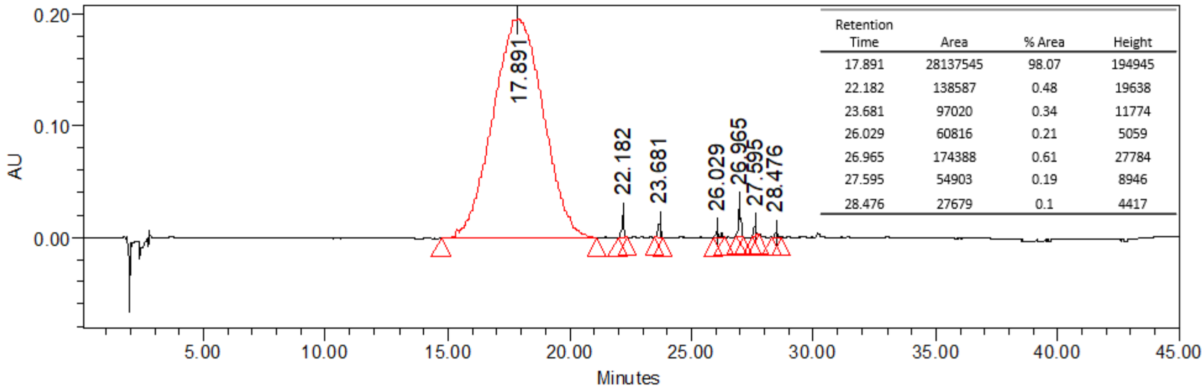

Retention Time	Area	% Area	Height
17.891	28137545	98.07	194945
22.182	138587	0.48	19638
23.681	97020	0.34	11774
26.029	60816	0.21	5059
26.965	174388	0.61	27784
27.595	54903	0.19	8946
28.476	27679	0.1	4417

Figure S55. HPLC Analysis

Compound 4m

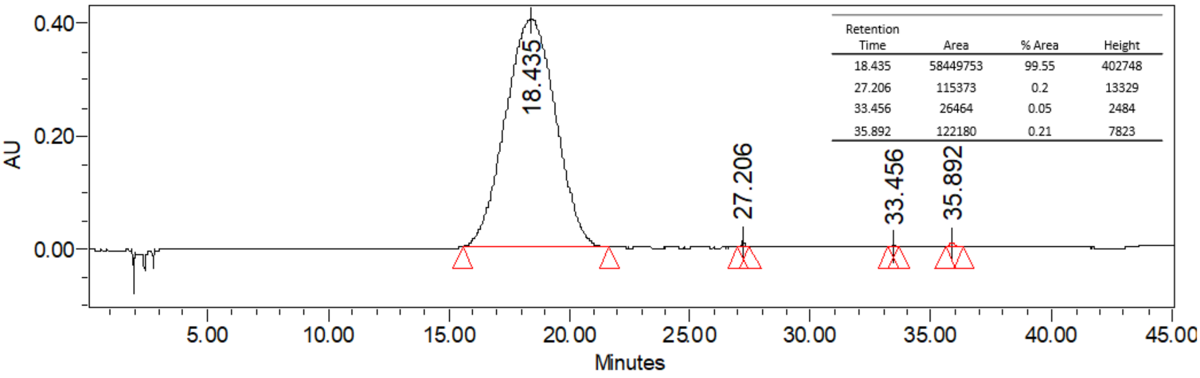

Retention Time	Area	% Area	Height
18.435	58449753	99.55	402748
27.206	115373	0.2	13329
33.456	26464	0.05	2484
35.892	122180	0.21	7823

Compound 4n

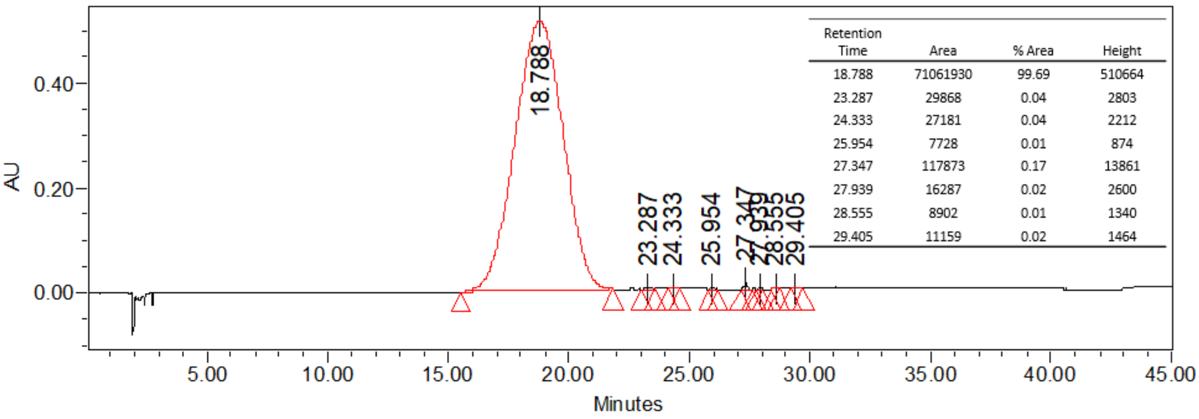

Retention Time	Area	% Area	Height
18.788	71061930	99.69	510664
23.287	29868	0.04	2803
24.333	27181	0.04	2212
25.954	7728	0.01	874
27.347	117873	0.17	13861
27.939	16287	0.02	2600
28.555	8902	0.01	1340
29.405	11159	0.02	1464

Figure S56. HPLC Analysis

Compound 8a

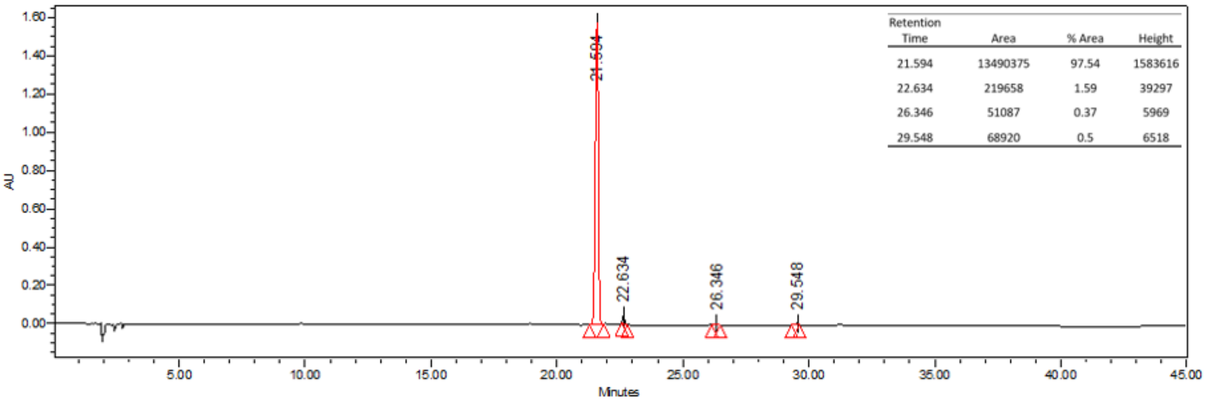

Retention Time	Area	% Area	Height
21.594	13490375	97.54	1583616
22.634	219658	1.59	39297
26.346	51087	0.37	5969
29.548	68920	0.5	6518

Compound 8b

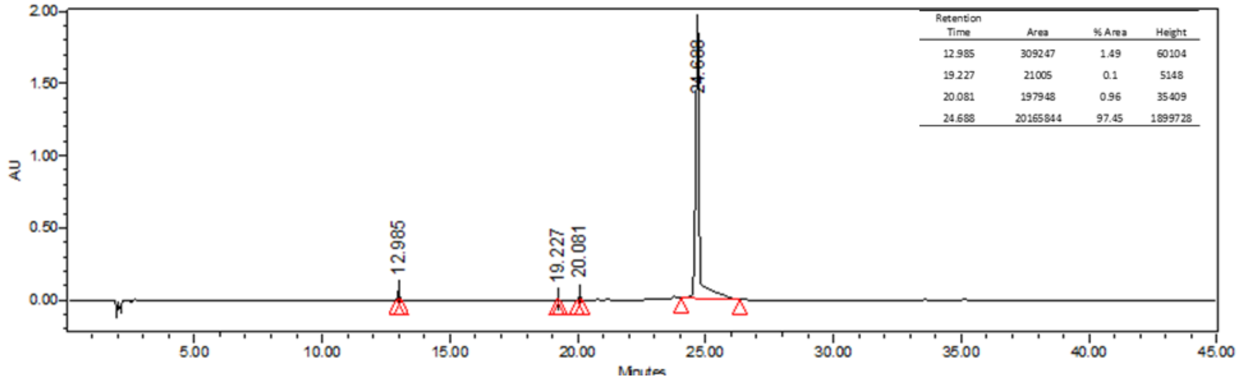

Retention Time	Area	% Area	Height
12.985	309247	1.49	60104
19.227	21005	0.1	5148
20.081	197948	0.96	35409
24.688	20165844	97.45	1899728

Compound 8c

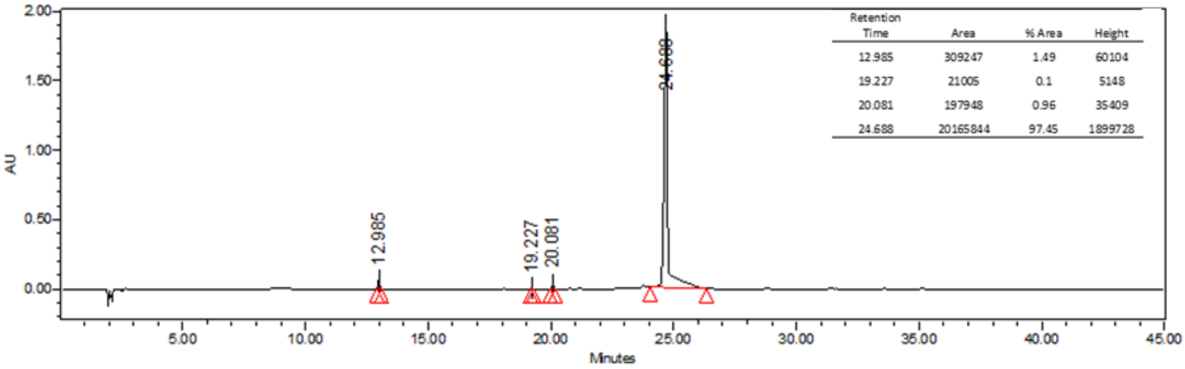

Retention Time	Area	% Area	Height
12.985	309247	1.49	60104
19.227	21005	0.1	5148
20.081	197948	0.96	35409
24.688	20165844	97.45	1899728

Figure S57. HPLC Analysis

Compound 8d

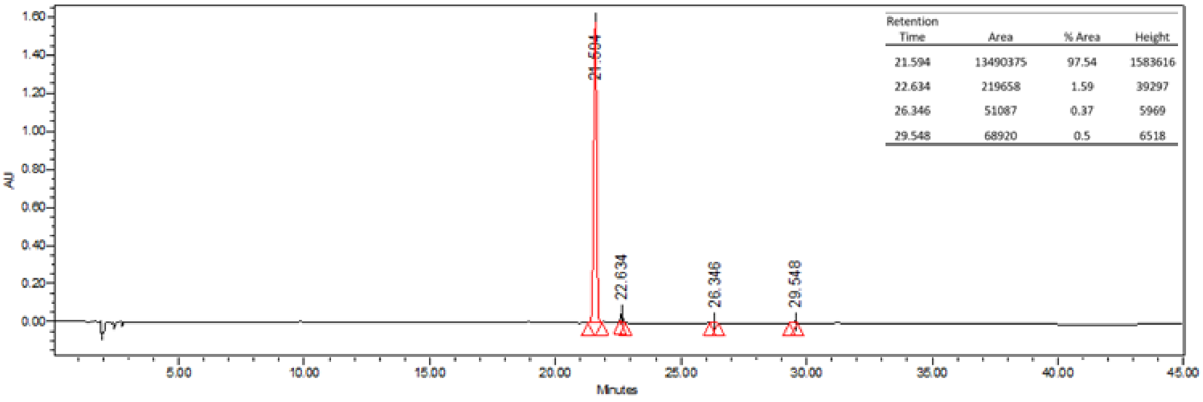

Compound 8e

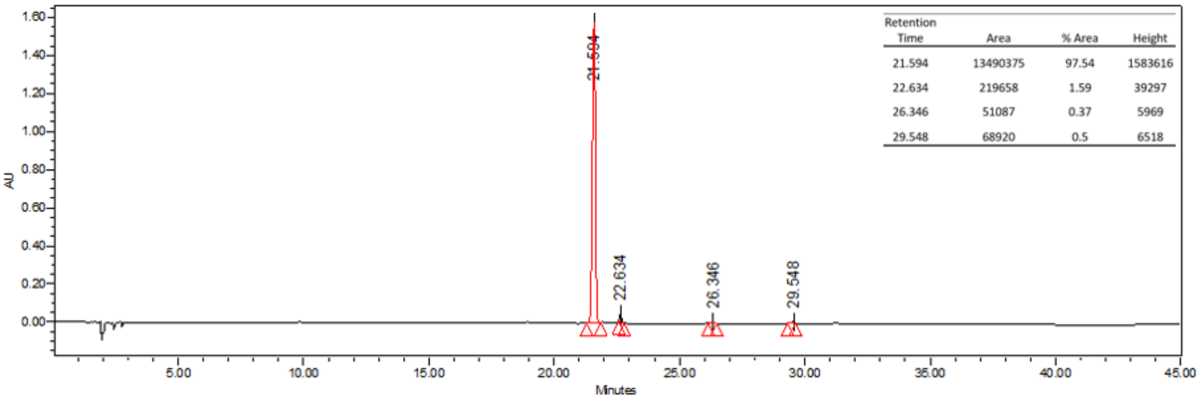

Compound 8f

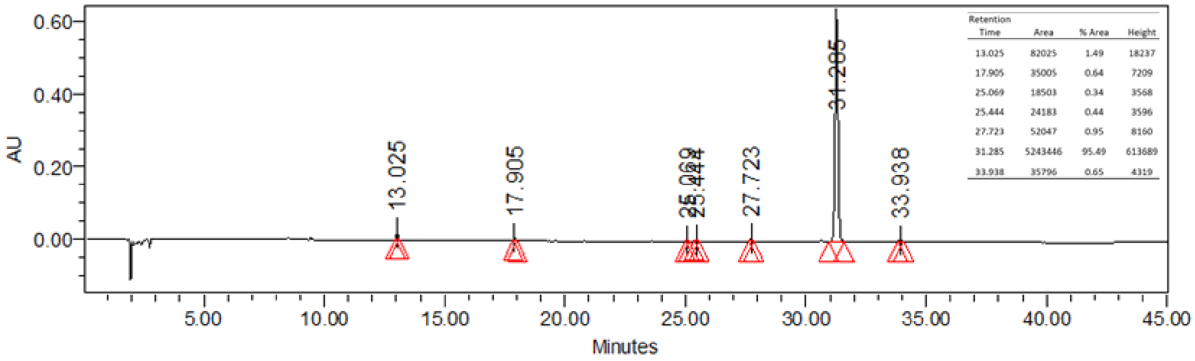

Figure S58. HPLC Analysis

Compound 8g

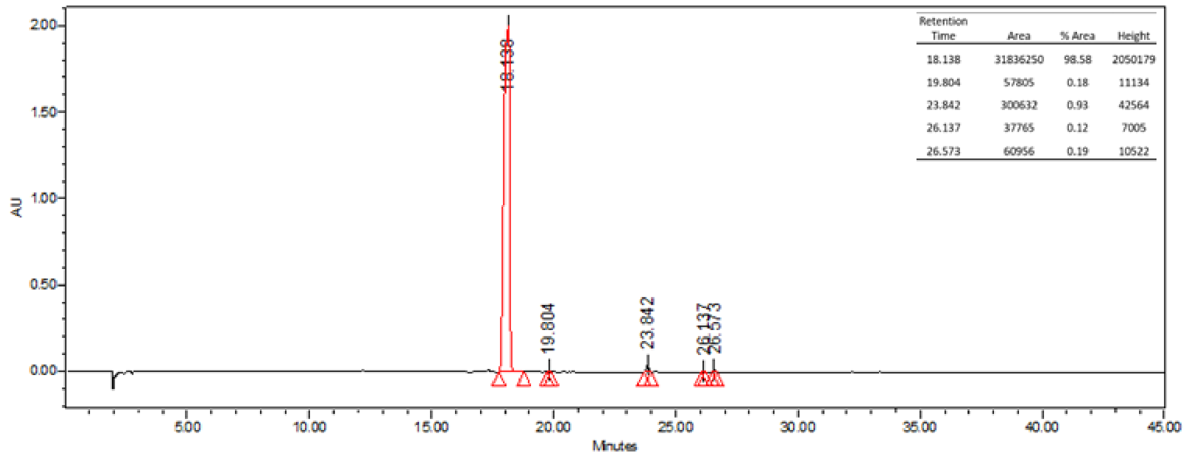

Retention Time	Area	% Area	Height
18.138	31836250	98.58	2050179
19.804	57805	0.18	11134
23.842	300632	0.93	42564
26.137	37765	0.12	7005
26.573	60956	0.19	10522

Compound 8h

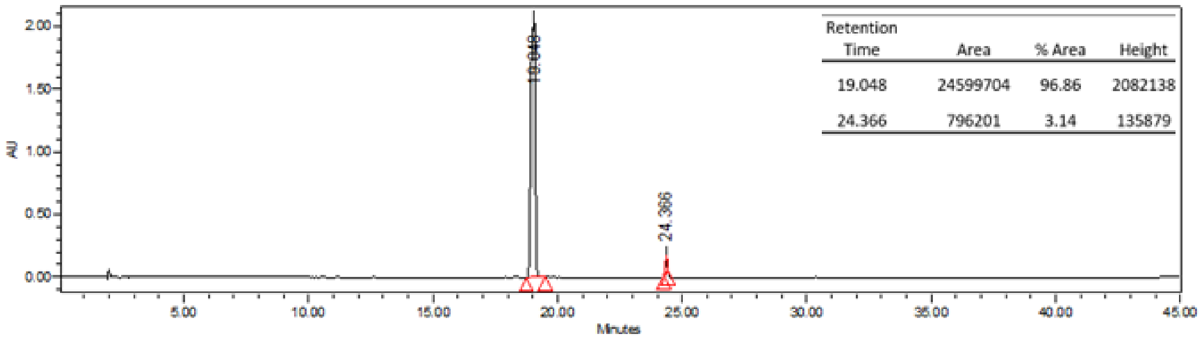

Retention Time	Area	% Area	Height
19.048	24599704	96.86	2082138
24.366	796201	3.14	135879

Compound 8i

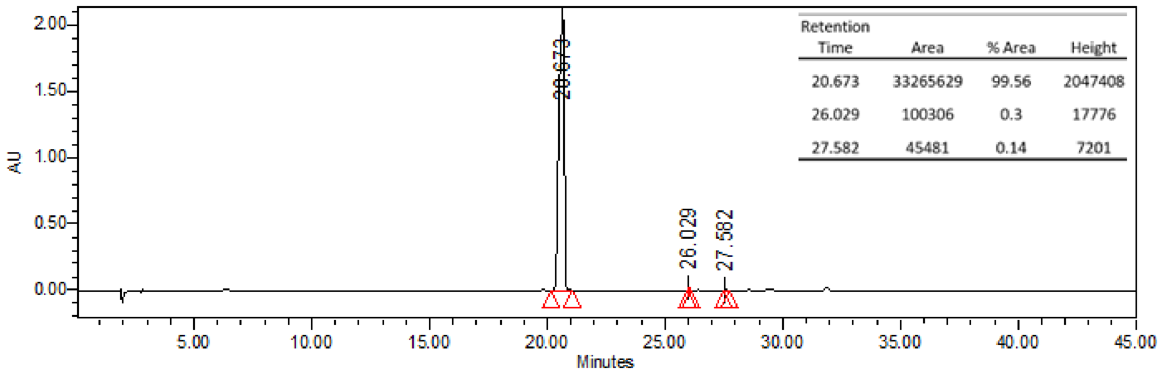

Retention Time	Area	% Area	Height
20.673	33265629	99.56	2047408
26.029	100306	0.3	17776
27.582	45481	0.14	7201

Figure S59. HPLC Analysis

Compound 8j

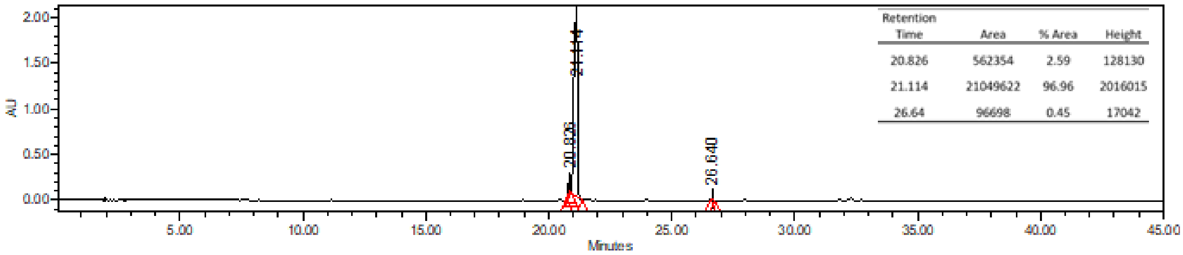

Retention Time	Area	% Area	Height
20.826	562354	2.59	128130
21.114	21049622	96.96	2016015
26.64	96698	0.45	17042

Compound 8k

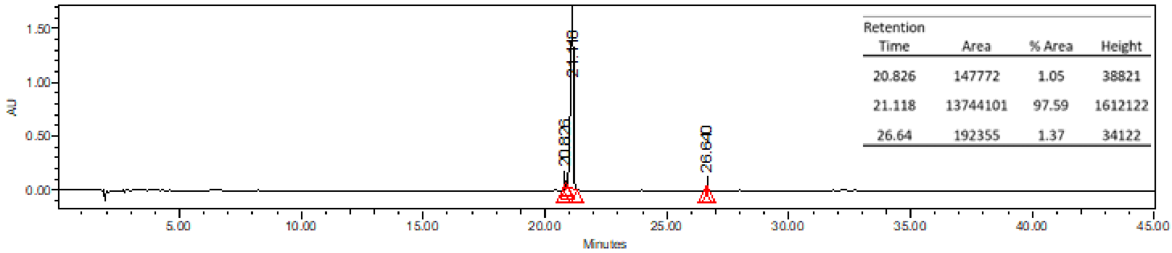

Retention Time	Area	% Area	Height
20.826	147772	1.05	38821
21.118	13744101	97.59	1612122
26.64	192355	1.37	34122

Figure S60. HRMS Analysis

Compound 4i

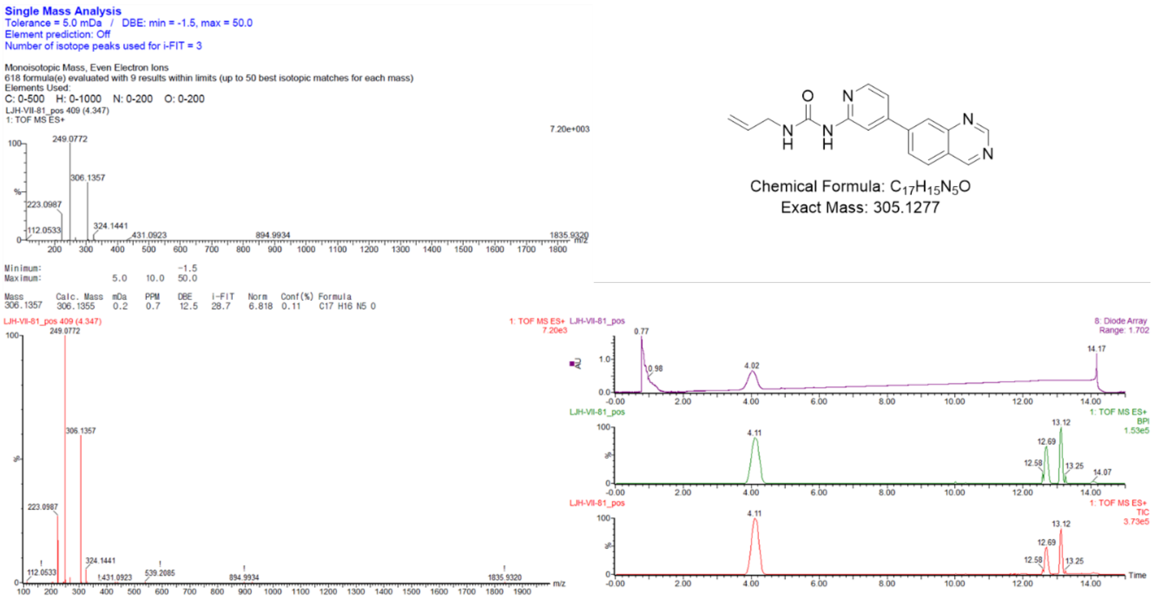

Compound 4j

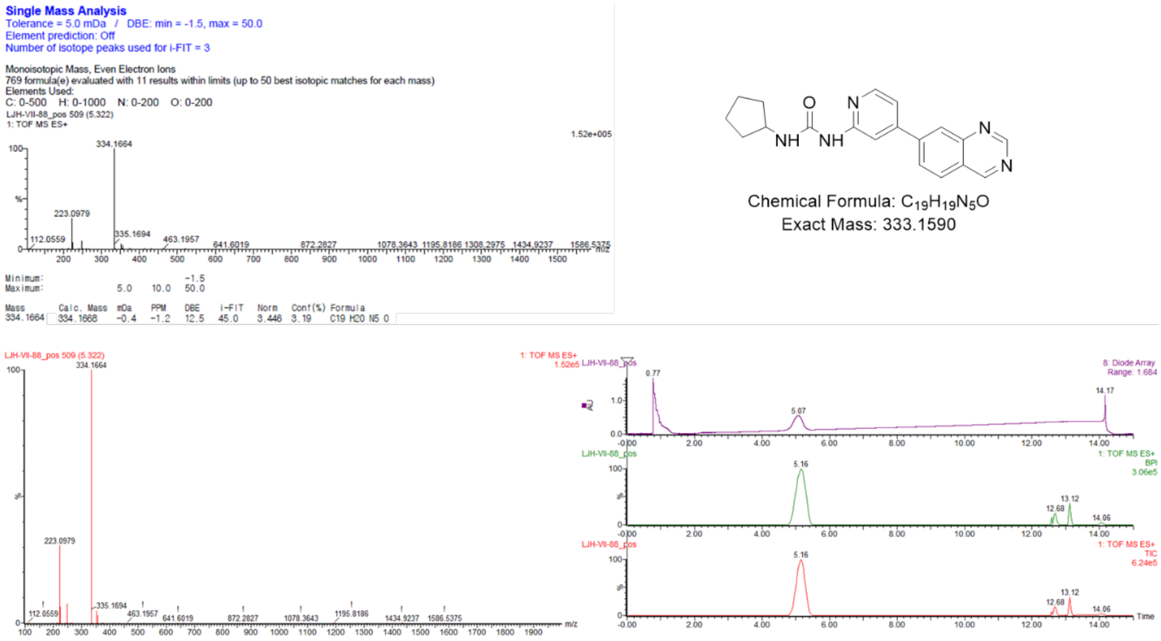

Figure S61. HRMS Analysis

Compound 4k

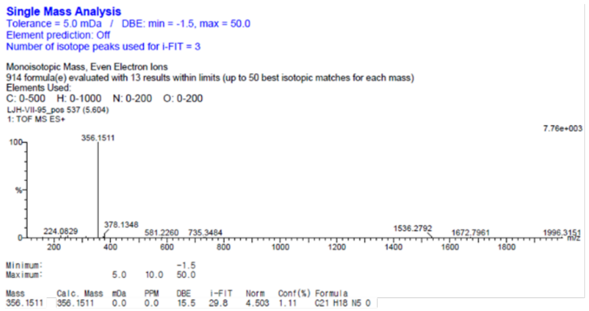

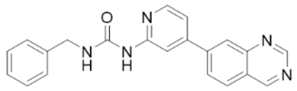

Chemical Formula: $C_{21}H_{17}N_5O$
Exact Mass: 355.1433

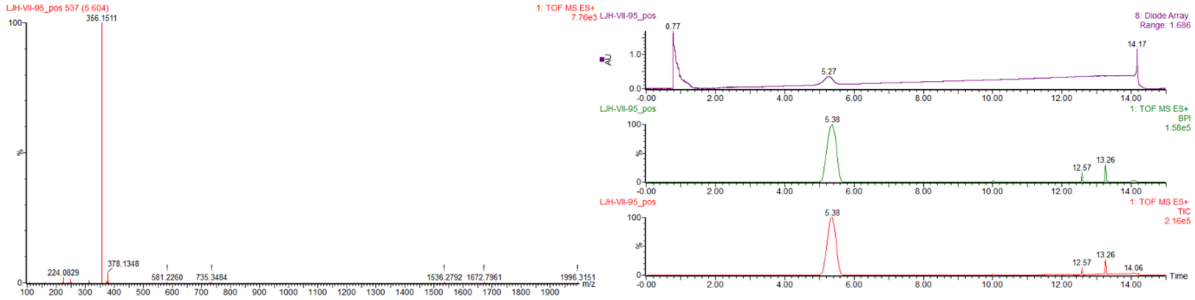

Compound 4l

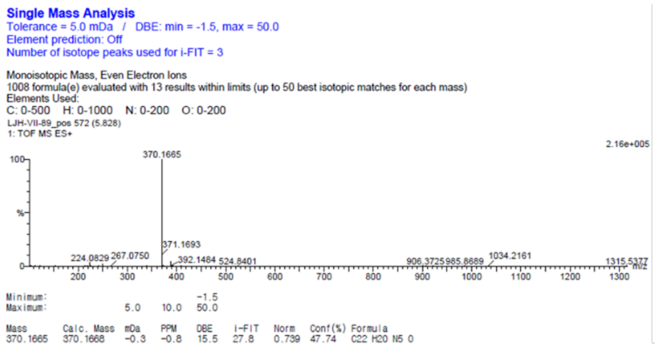

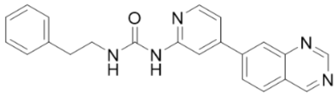

Chemical Formula: $C_{22}H_{19}N_5O$
Exact Mass: 369.1590

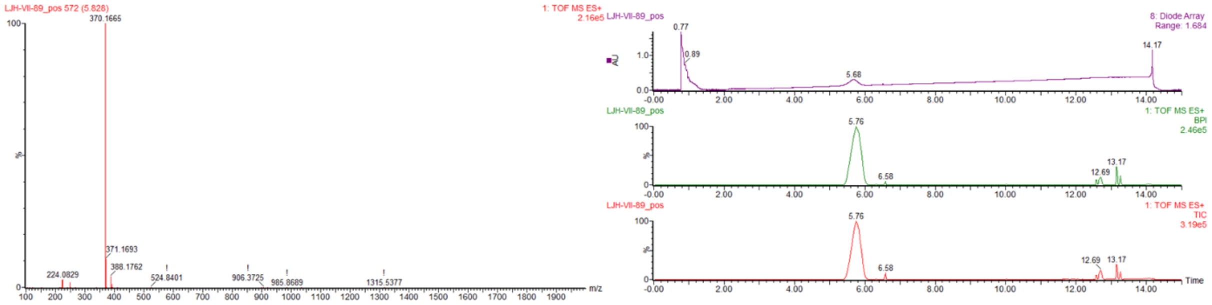

Figure S62. HRMS Analysis

Compound 4m

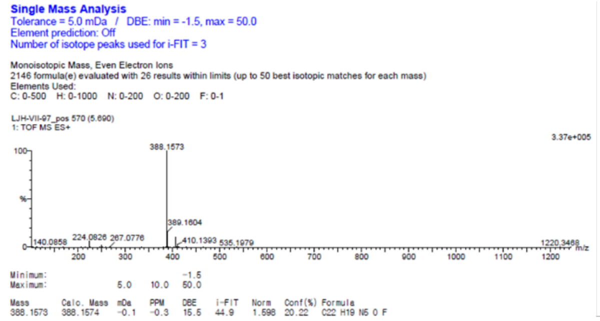

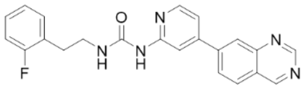

Chemical Formula: $C_{22}H_{18}FN_5O$
Exact Mass: 387.1495

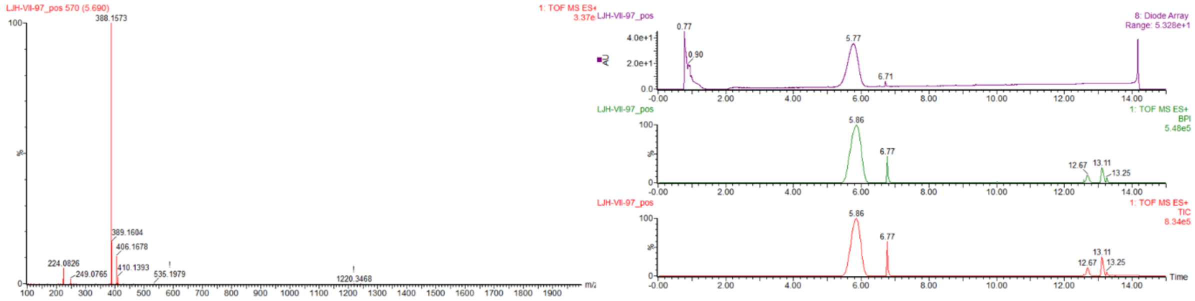

Compound 4n

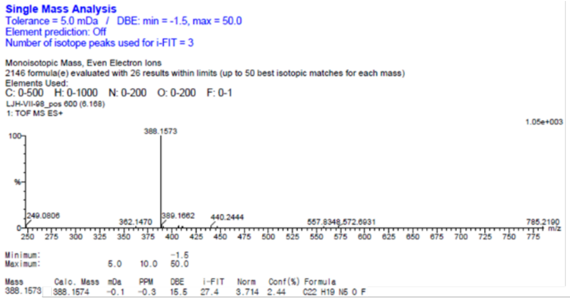

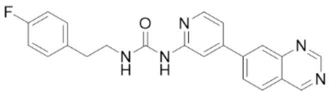

Chemical Formula: $C_{22}H_{18}FN_5O$
Exact Mass: 387.1495

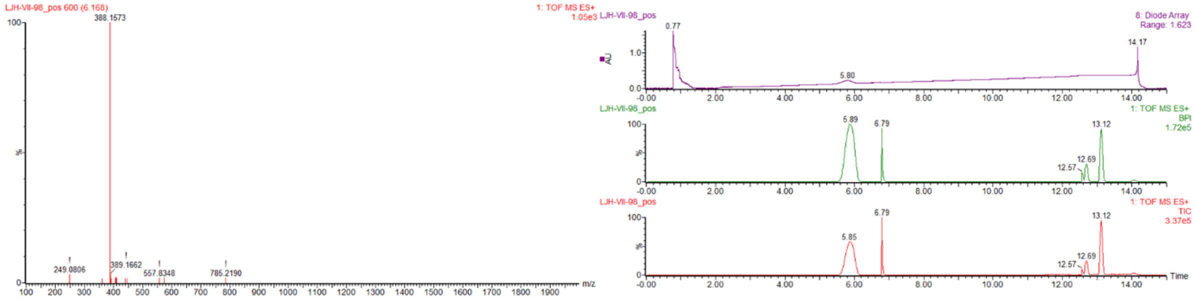

Figure S63. HRMS Analysis

Compound 8a

Single Mass Analysis
Tolerance = 5.0 mDa / DBE: min = -1.5, max = 50.0
Element prediction: Off
Number of isotope peaks used for i-FIT = 3

Monoisotopic Mass, Even Electron Ions
937 formula(e) evaluated with 13 results within limits (up to 50 best isotopic matches for each mass)
Elements Used:
C: 0-500 H: 0-1000 N: 0-200 O: 0-200
GS-VIII-61_pos 926 (9.258)
1: TOF MS ES+

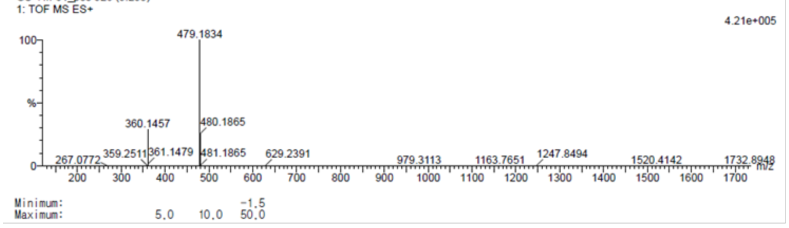

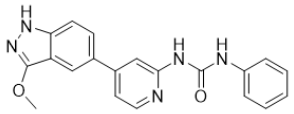

Chemical Formula: $C_{20}H_{17}N_5O_2$
Exact Mass: 359.1382

Mass	Calc. Mass	mDa	PPM	DBE	i-FIT	Norm	Conf(%)	Formula
360.1457	360.1460	-0.3	-0.8	14.5	36.8	2.769	6.27	C20 H18 N5 O2

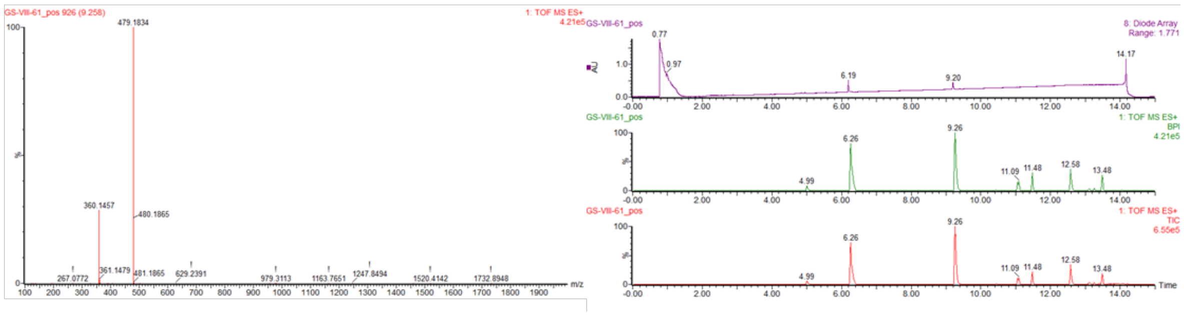

Compound 8b

Single Mass Analysis
Tolerance = 5.0 mDa / DBE: min = -1.5, max = 50.0
Element prediction: Off
Number of isotope peaks used for i-FIT = 3

Monoisotopic Mass, Even Electron Ions
1998 formula(e) evaluated with 27 results within limits (up to 50 best isotopic matches for each mass)
Elements Used:
C: 0-500 H: 0-1000 N: 0-200 O: 0-200 F: 0-1
GS-VIII-62_pos 692 (6.902)
1: TOF MS ES+

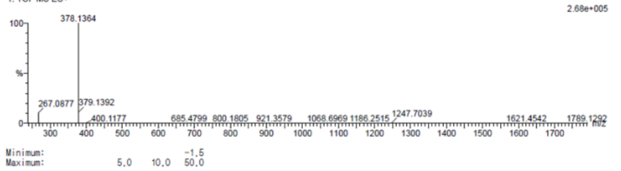

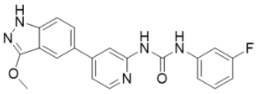

Chemical Formula: $C_{20}H_{16}FN_5O_2$
Exact Mass: 377.1288

Mass	Calc. Mass	mDa	PPM	DBE	i-FIT	Norm	Conf(%)	Formula
378.1364	378.1366	-0.2	-0.5	14.5	39.2	2.715	6.62	C20 H17 N5 O2 F

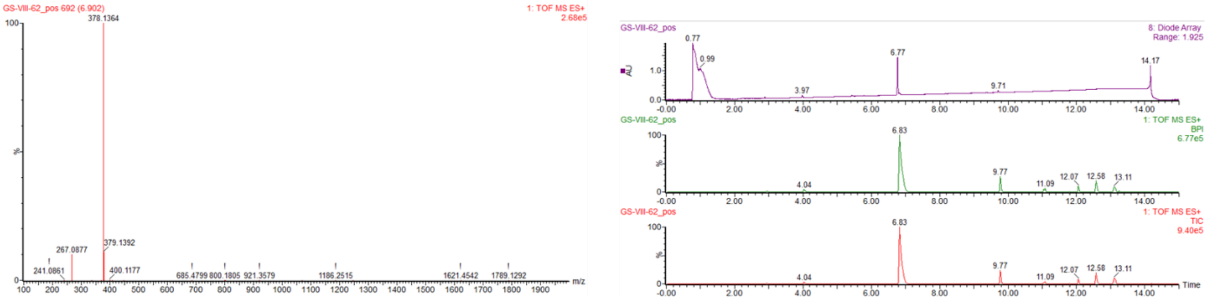

Figure S64. HRMS Analysis

Compound 8c

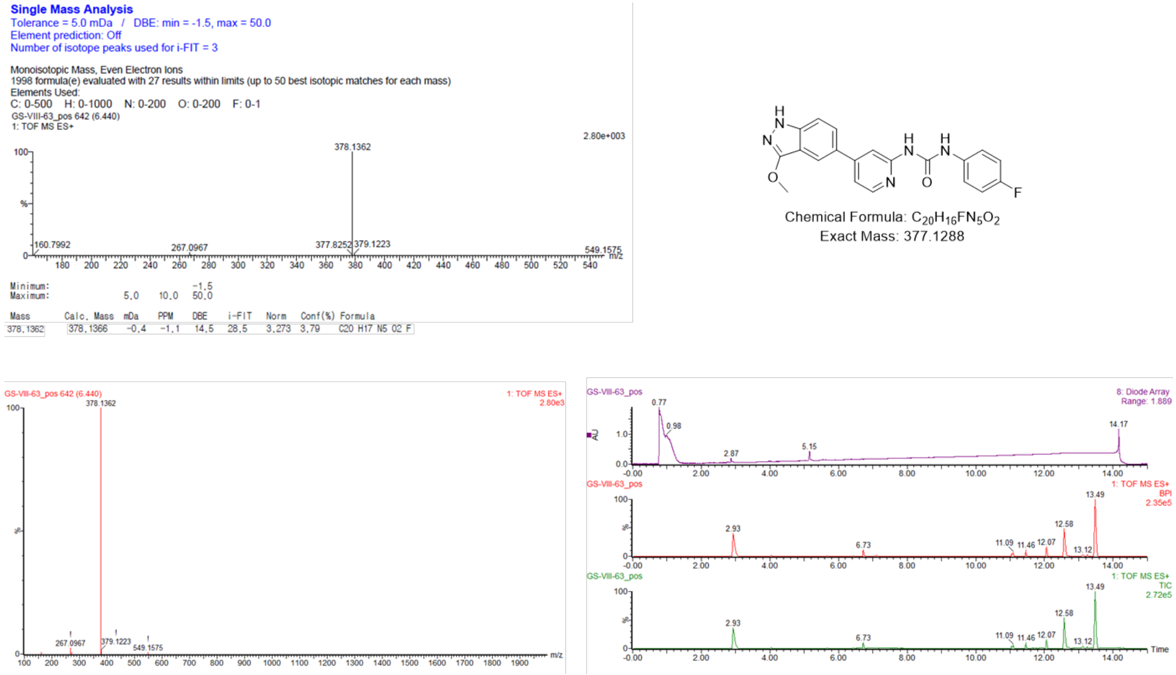

Compound 8d

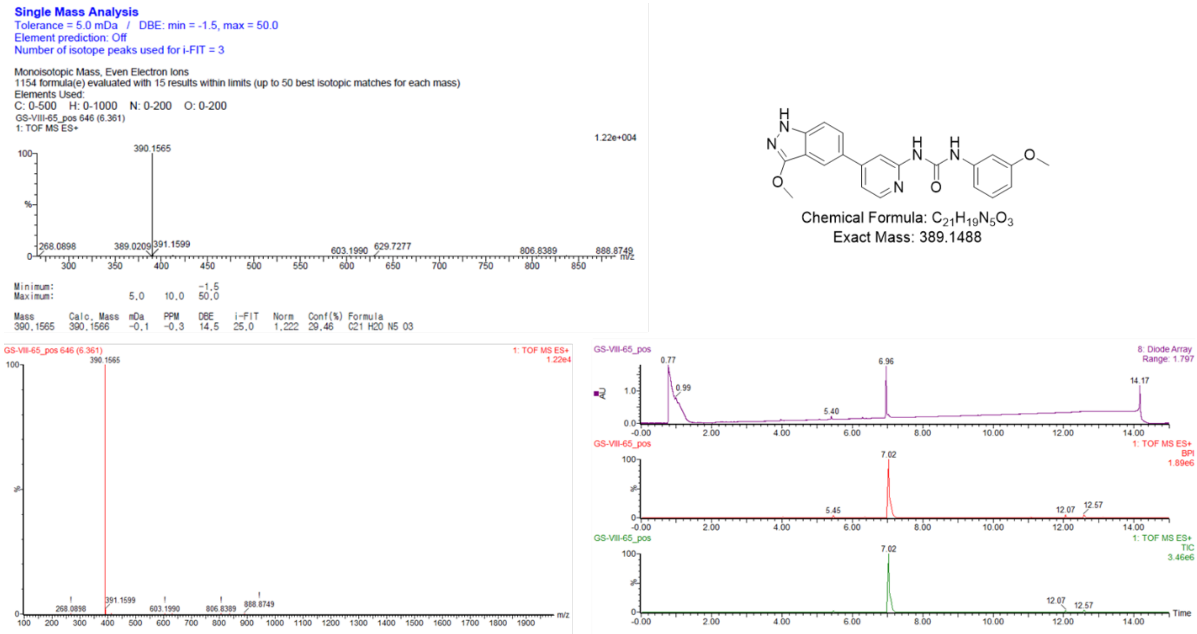

Figure S65. HRMS Analysis

Compound 8e

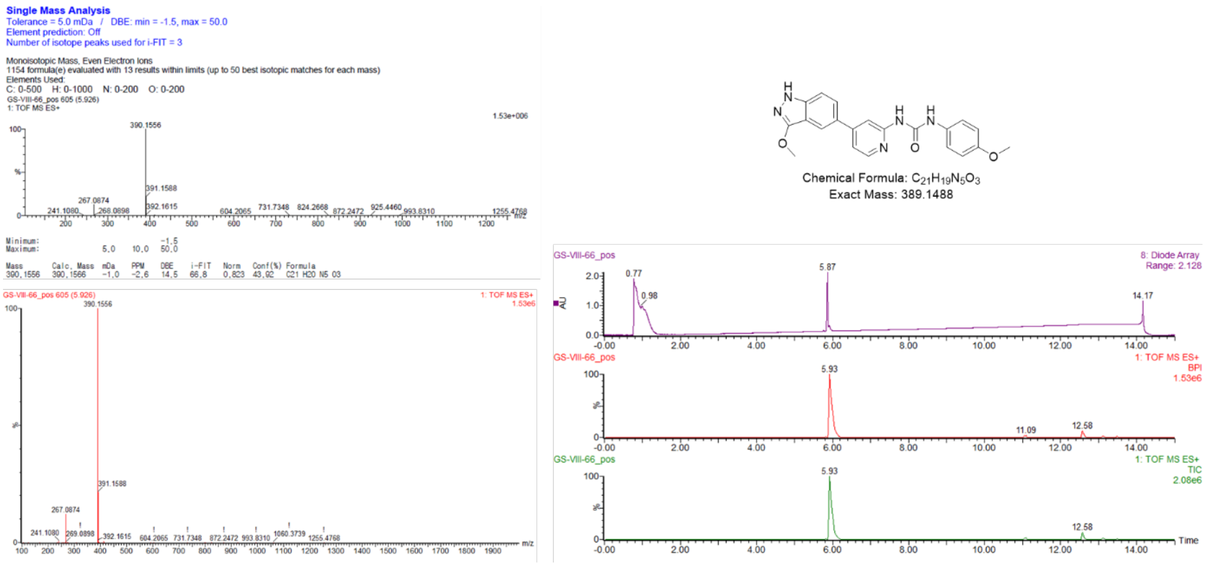

Compound 8f

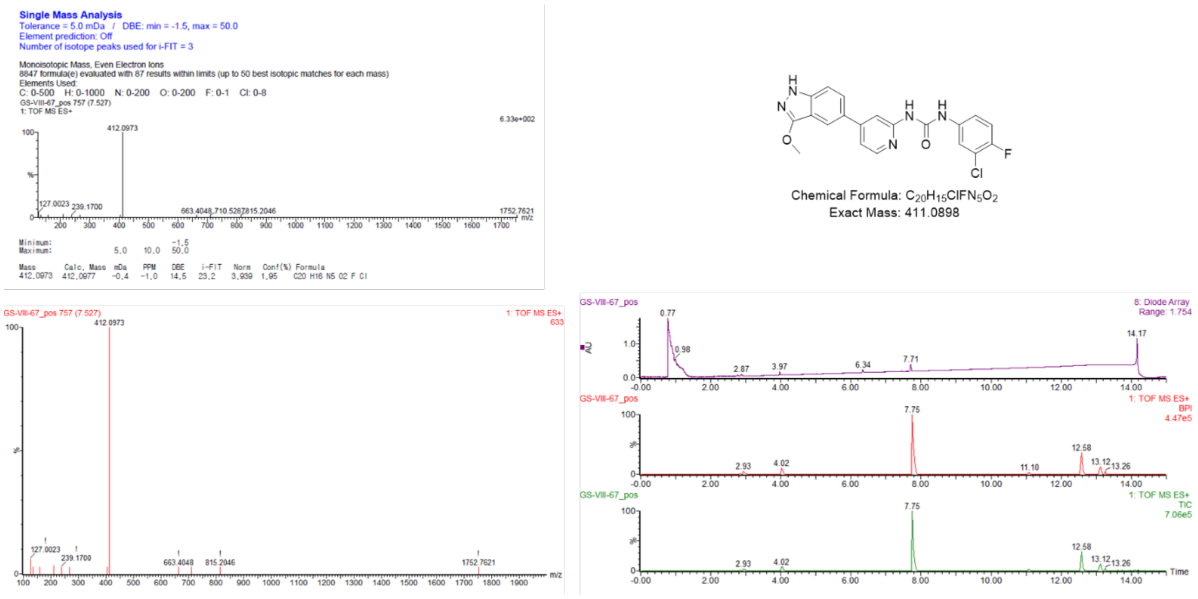

Figure S66. HRMS Analysis

Compound 8g

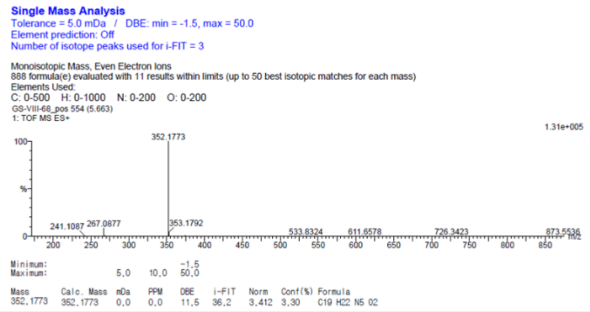

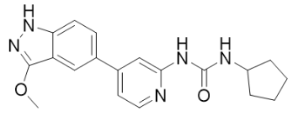

Chemical Formula: $C_{19}H_{21}N_5O_2$
Exact Mass: 351.1695

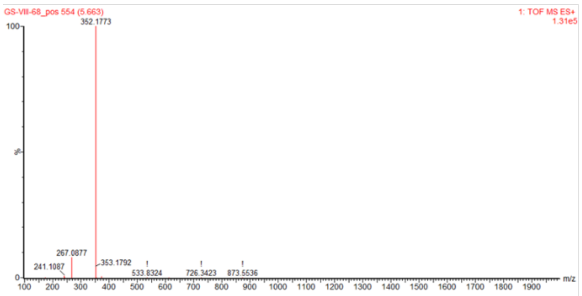

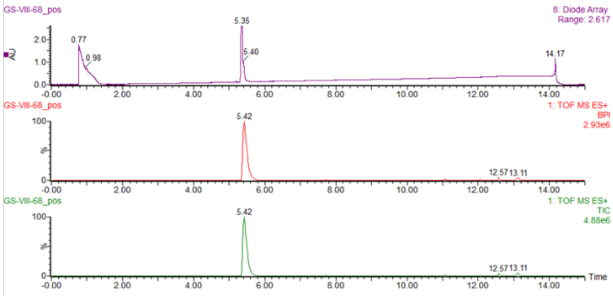

Compound 8h

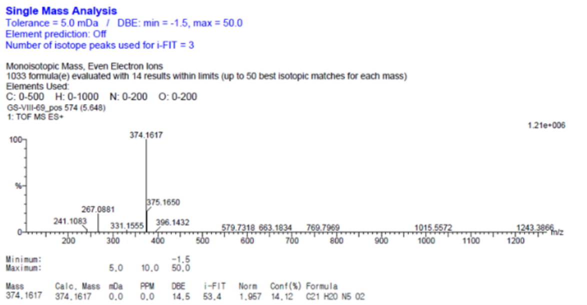

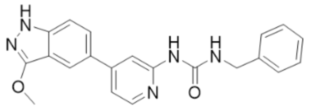

Chemical Formula: $C_{21}H_{19}N_5O_2$
Exact Mass: 373.1539

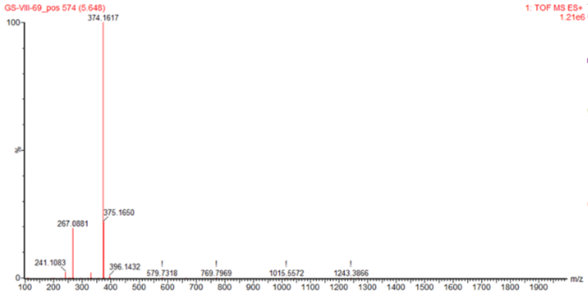

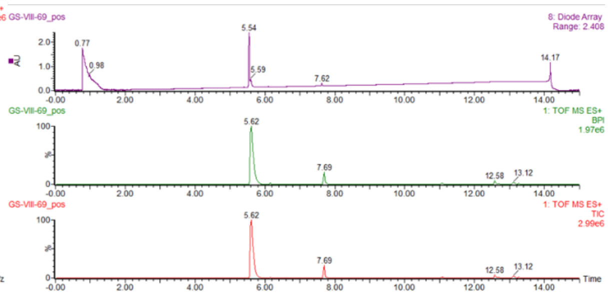

Figure S67. HRMS Analysis

Compound 8i

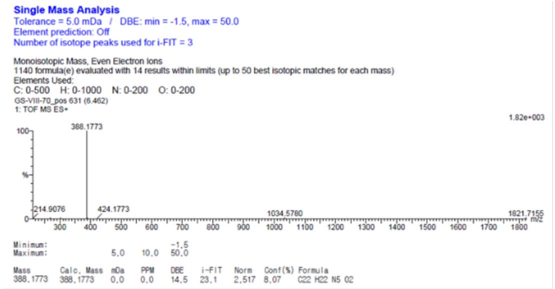

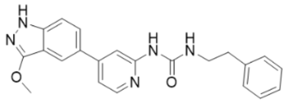

Chemical Formula: $C_{22}H_{21}N_5O_2$
Exact Mass: 387.1695

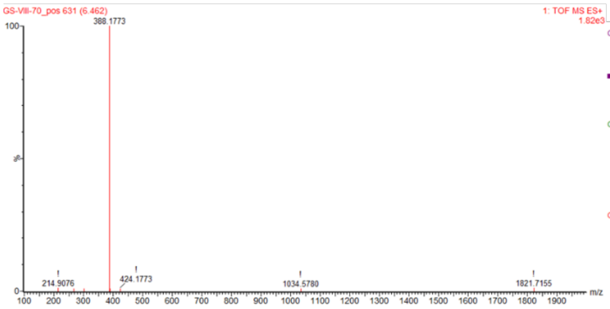

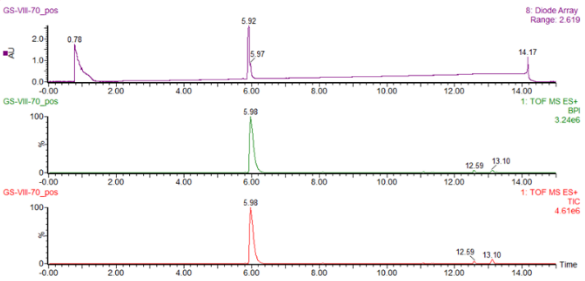

Compound 8k

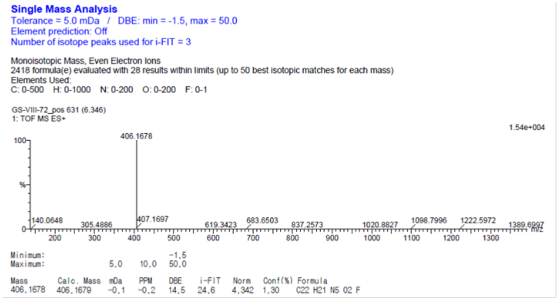

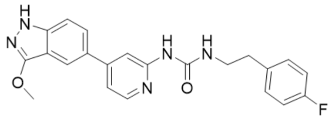

Chemical Formula: $C_{22}H_{20}FN_5O_2$
Exact Mass: 405.1601

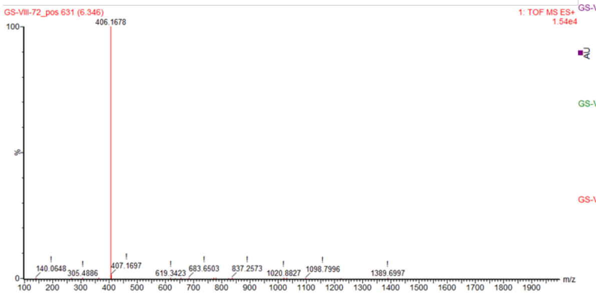

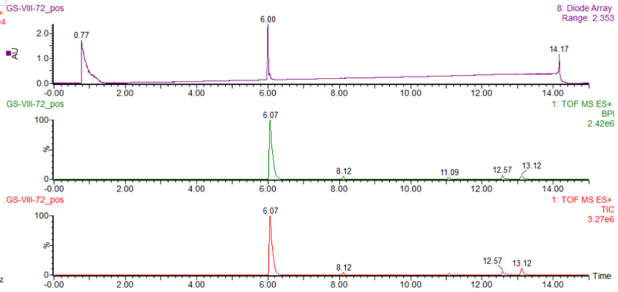

Figure S68. 2D structure of Irinotecan and Tivozanib

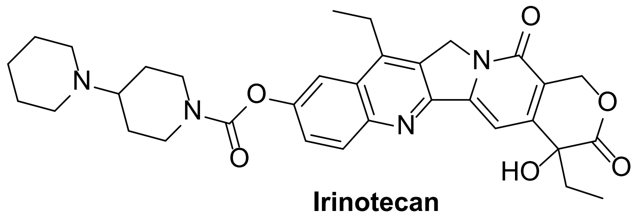

Irinotecan

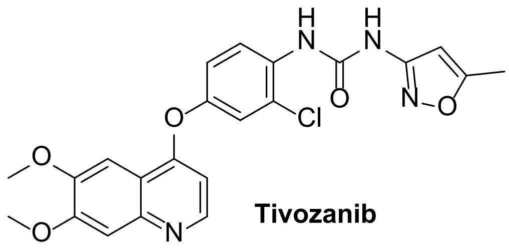

Tivozanib

Figure S69. 2D interaction diagram of Tivozanib

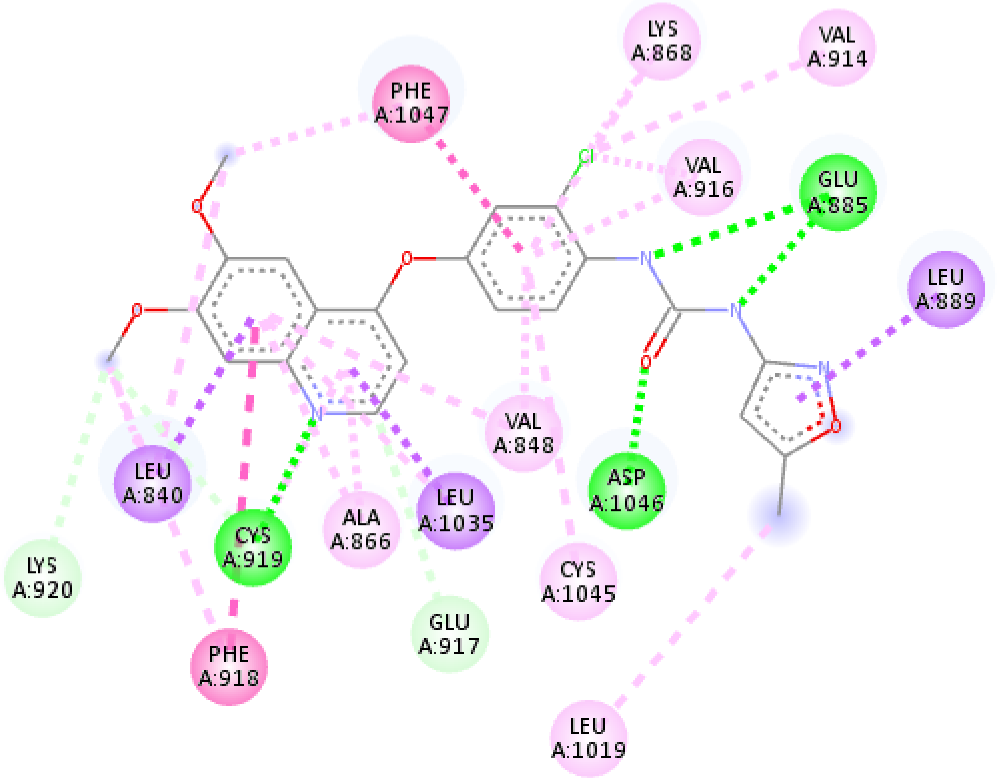

Figure S70. RMSD plots of the protein complexes of compounds 8a (orange), 8h (blue) and 8i (magenta)

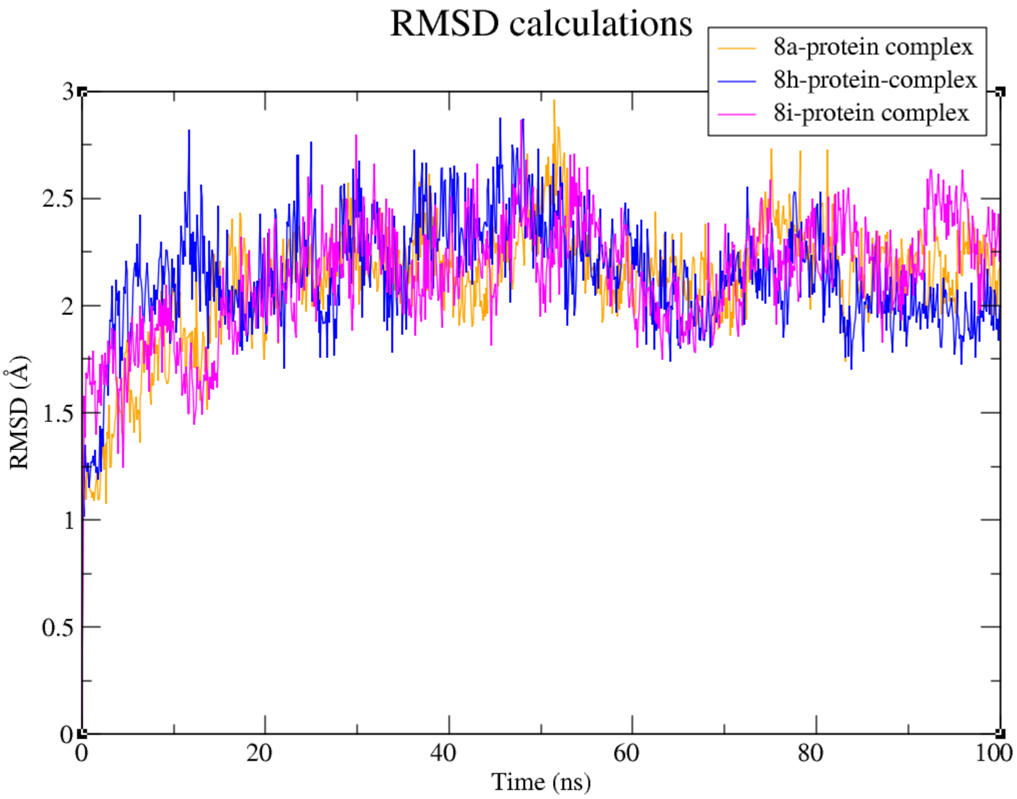

Figure S71. Protein-ligand contact diagrams for compounds 8a, 8h and 8i; green-hydrogen bonds, purple; hydrophobic interactions, bule-water bridges.

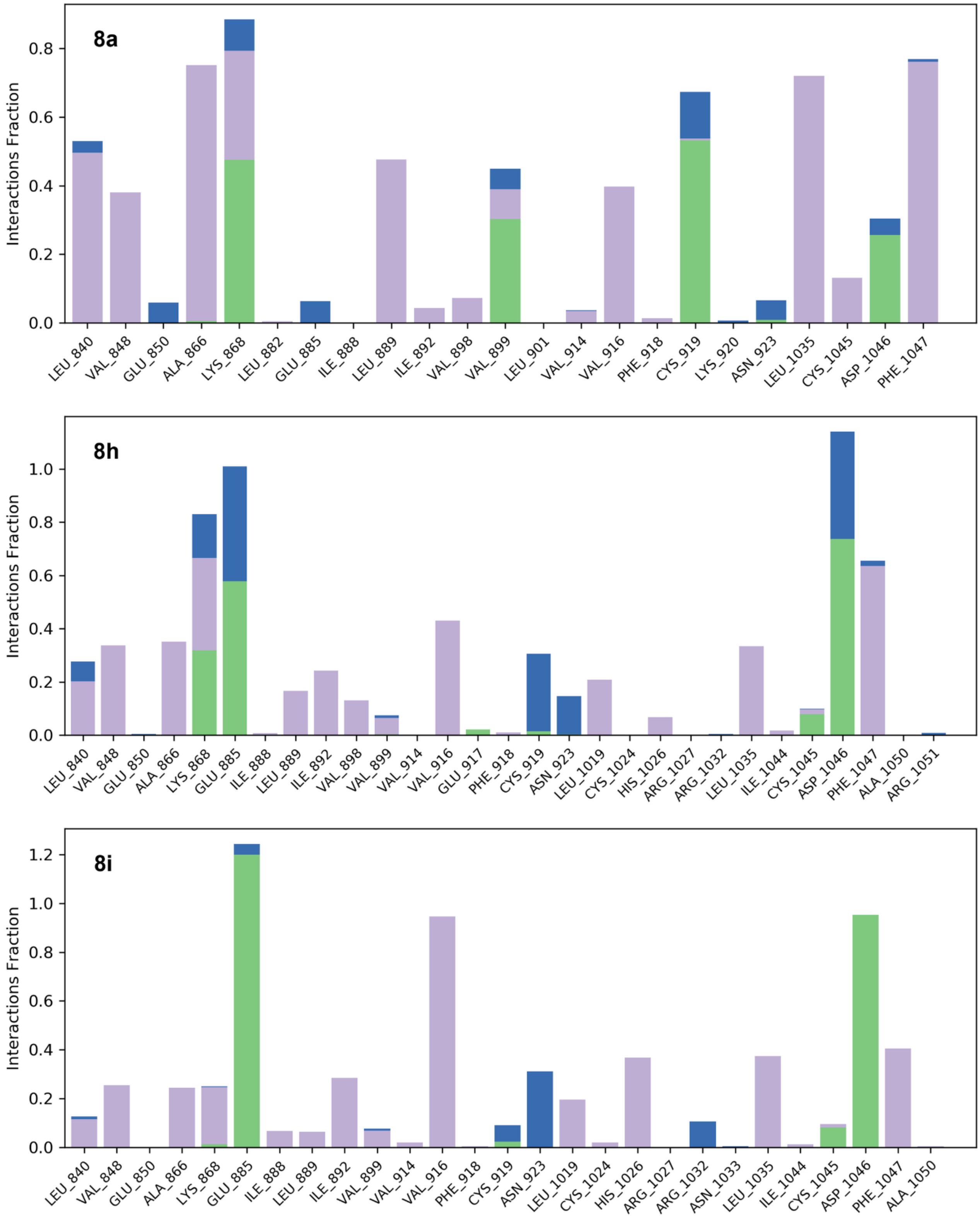

Supplement: Supplementary file 1 [file molecules-28-04952-s001.zip › molecules-2466018-supplementary.pdf]
